# On-Resin Synthesis and Late-Stage Functionalization of Macrocyclic Atosiban Mimetics via 5‑Iodo-1,4-triazoles

**DOI:** 10.1021/acs.orglett.5c03507

**Published:** 2025-09-26

**Authors:** Oscar A. Shepperson, Michael A. Malone, Kirsty I. M. Arnott, Ryan J. Brown, Andrew G. Jamieson

**Affiliations:** School of Chemistry, Advanced Research Centre, 3526 University of Glasgow, 11 Chapel Lane, Glasgow G11 6EW, United Kingdom

## Abstract

We report an on-resin strategy for synthesizing 5-iodo-1,4-disubstituted-1,2,3-triazole-containing macrocyclic peptides as multifunctional disulfide bridge mimetics. Optimized Cu­(I)-catalyzed azide-alkyne cycloaddition (CuAAC) and Suzuki–Miyaura conditions enabled late-stage arylation at the triazole 5-position. This approach offers a novel strategy for the fluorescent and biotin functionalization of peptides. Structural analysis revealed that the 5-iodo substituent influences the peptide conformation. These findings establish 5-iodo-1,4-triazoles as versatile, tunable motifs for macrocyclization and functionalization, expanding the chemical space accessible to macrocyclic peptide chemical biology tools and therapeutics.

## Graphical abstract

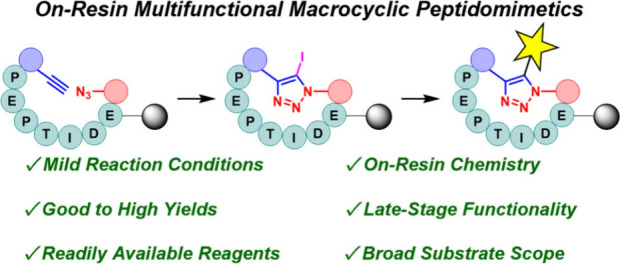

Macrocyclic peptides are emerging as powerful tools in chemical biology and promising leads in drug discovery, owing to their enhanced binding affinity, selectivity, and proteolytic stability.^1,2^ A diverse array of macrocyclization strategies has been developed, encompassing both native functionalities, such as amide, disulfide, thioether, and ester linkages, and non-native chemistries designed to expand structural diversity and improve pharmacological properties.^3,4^ Among these, the incorporation of 1,2,3-triazoles as macrocyclization motifs in peptides has garnered significant attention due to the efficiency, reliability, and modularity of the Cu­(I)-catalyzed azide-alkyne cycloaddition (CuAAC) reaction.^5,6^ Triazoles have thus been widely employed as versatile cyclization motifs in peptide and peptidomimetic chemistry. Beyond their synthetic utility, their true value lies in their function as bioisosteres of native peptide linkages. Notably, 1,4-disubstituted triazoles have been shown to effectively mimic trans-amide bonds, while 1,5-disubstituted triazoles resemble *cis*-amide geometries.^7^ 1,2,3-Triazole has also attracted significant attention as a cyclization motif that mimics a disulfide bridge. Previous studies, including our own, have shown that the Cα–Cα distance in a triazole formed between β-azidohomoalanine and propargyl glycine closely matches that of a native cysteine disulfide bridge.^8–10^

In the course of our studies on the use of triazoles as peptide disulfide bridge mimetics, we hypothesized that introducing a third substituent at the 5-position of the triazole could create a handle for late-stage diversification. This functionalization would enable the incorporation of chemical biology probes or, alternatively, provide a site for structural diversification to explore a new chemical space and potentially improve ligand selectivity and binding affinity. The synthesis and incorporation of 5-iodo-1,4-disubstituted-1,2,3-triazoles into small molecule imaging agents,^11^ nucleotides, and oligosaccharides have previously been reported; however, to our knowledge, no applications to peptide disulfide bridge mimetics have been reported.^12–14^ We therefore set out to develop a new class of peptidomimetics featuring a multifunctional triazole core that simultaneously acts as a disulfide bridge mimic and provides a handle for late-stage functional diversification.

Here, we report the development of an on-resin strategy for the synthesis of 5-iodo-1,4-disubstituted-1,2,3-triazole-containing macrocyclic peptides. The iodotriazole moiety enabled late-stage diversification via Suzuki–Miyaura cross-coupling, including arylation with an aniline derivative subsequently functionalized with fluorescein and biotin to generate chemical biology tool compounds.

To evaluate the feasibility of our proposed chemistry, we selected the synthetic peptide atosiban, a nonapeptide oxytocin receptor antagonist cyclized via a disulfide bridge between the side chain of Cys6 and an N-terminal 3-mercaptopropionic acid (Mpa) residue.^15^ Atosiban was chosen for its straightforward linear synthesis, well-defined disulfide macrocycle, and diverse side-chain functionalities, making it a good model for assessing the scope and versatility of our macrocyclization strategy.

Using automated Fmoc/^*t*^Bu-based solid-phase peptide synthesis (Fmoc-SPPS), we efficiently prepared linear precursor 1* (where * denotes the on-resin peptide), incorporating azidohomoalanine (Aha) at position six and pentynoic acid (PyA) at the N-terminus. Detailed synthetic protocols and characterization data are provided in the Supporting Information (SI Table S1, SI Figures S8–S25). Macrocyclization to generate the 1,4-triazole-containing atosiban mimetic **2** was achieved via copper-catalyzed azide–alkyne cycloaddition (CuAAC) under previously reported conditions (Scheme 1).^6,16^

**Scheme 1:**
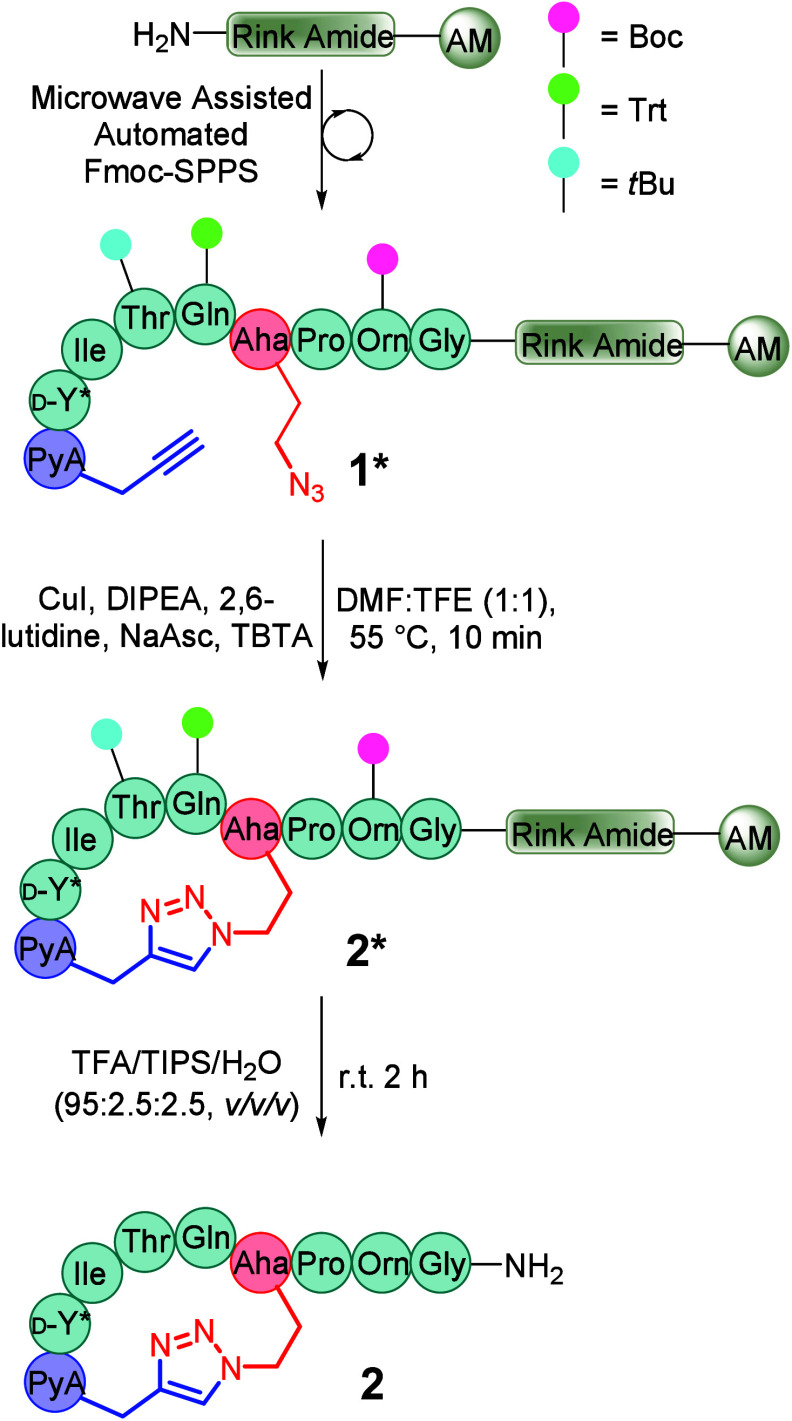
Solid-Phase Synthesis of 1,4-Triazole Atosiban Mimetic **2** [Fn sch1-fn2]

a
d-Tyr­(OEt) (d-Y*); pentynoic acid (PyA), blue; azidohomoalanine (Aha), red. *: denotes the on-resin peptide.

Next, we developed the chemistry required to produce a bifunctional triazole-cyclized analogue of **3**. As 5-iodo-1,4-triazole formation had solely been achieved using solution phase synthesis, we felt the need to investigate a variety of conditions to determine the optimal methodology required to yield the desired on-resin 5-iodo-1,4-triazole, 3*, in high conversion (Table 1).^13,17^

**Table 1:**
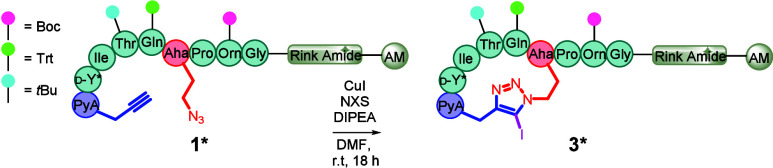
Reaction Conditions and Conversions for the On-Resin Formation of 5-Iodo-1,4-Triazole 3*

	Reaction Conditions				% Area Ratio^a^			
Entry	CuI Equiv.	NXS	Oxidant Equiv.	DIPEA Equiv.	**1**	Dimer [2M+3H]^3+^	**2**	**3**
1^b^	1.1	NBS	1.2	2	22	5	20	53
2^b^	1.1	NCS	1.2	2	57	2	7	34
3^b^	1.1	NIS	1.2	2	75	1	24	0
4	1.1	NBS	1.2	2	11	4	11	73
5	2.2	NBS	2	2	52	6	40	2
6	2.2	NBS	1.2	2	1	3	11	85
7^c^	2.2	NBS	1.2	2	3	4	11	82

a % area determined at 214 nm. *: Denotes the on-resin peptide.

b These reactions were carried out in the absence of light.

c Denotes large scale (0.3 mmol) reaction.

We first evaluated the choice of *N*-halosuccinimide (NXS) oxidant, identifying NBS was the most effective of the halogen series tested (Table 1, Entries 1–3). Reactions were initially conducted under light exclusion due to the reported light sensitivity of NXS reagents; however, performing the reaction without the removal of light resulted in improved conversion to the desired 5-iodo-1,4-triazole product 3* (Table 1, Entry 4). Increasing the NBS concentration from 1.2 to 2 equiv significantly reduced product formation (Table 1, Entry 5), whereas increasing CuI loading from 1.1 to 2 equiv led to an 85% conversion to 3* with minimal dehalogenated byproduct observed (Table 1, Entry 6). Reaction conversions were assessed by cleaving a small portion of the washed peptidyl resins after exposure to the specified conditions, with analysis performed using reverse-phase (RP)-HPLC and liquid chromatography–mass spectrometry (LCMS). The successful formation of the 5-iodo product **3** was confirmed by a change in hydrophobicity, indicated by a shift in retention time (*t*_R_ = 25.6 min to *t*_R_= 24.3 min), alongside an expected mass increase of *m*/*z* = 126, consistent with the substitution of a proton with iodine. With the optimal conditions for the on-resin synthesis of the 5-iodo-1,4-triazole peptidyl resin 3* established, we successfully scaled up the reaction 30-fold to 0.3 mmol without any loss in reaction efficiency or conversion, highlighting the amenability to large scale on-resin synthesis (Table 1, Entry 7).

As *in situ* iodination of tyrosine residues has been reported as an undesired side product when employing iodonating reagents, we verified selective iodination of our target site using ^1^H NMR.^18^ To confirm our modification, we employed 1,4-triazole **2** as a reference compound, allowing us to distinguish the presence or absence of a proton at the 5-position of the triazole (SI Figures S17 and S19). Of interest, the ^1^H NMR spectrum of **2** displayed a characteristic splitting pattern consistent with the presence of *cis/trans* isomers of Pro^7^ in an unassigned 2:1 ratio (identified as proline isomerism by variable temperature ^1^H NMR) (Figure 1A).

**Figure 1:**
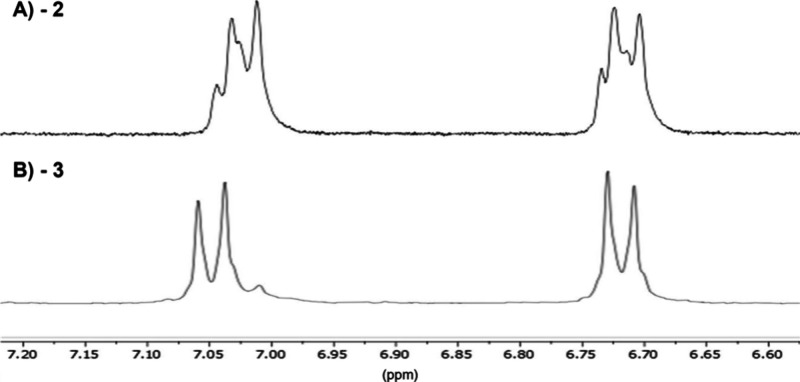
^1^H NMR comparison of aromatic region for peptides (A) **2** and (B) **3**.

This observation aligns with previous literature reports and is consistent with similar behavior observed in related neuropeptide nonapeptides.^15,19^ However, with the introduction of iodine at the 5-position of the triazole (**3**), no such splitting was observed, suggesting that iodination of the 5-position of the triazole may enforce a sterically enforced conformational lock on the peptide (Figure 1B) (Supporting Information Figures S17–S22).

With 5-iodo-1,4-triazole peptide 3* in hand, we sought to leverage the unique selective reactivity of the iodine on the heterocyclic triazole ring. We envisaged implementing Suzuki–Miyaura cross coupling conditions to facilitate a sp^2^-carbon–carbon bond forming reaction, resulting in novel, on-resin chemistry to create a highly diverse multifunctional moiety (SI Scheme S1).^20^ Initial screening investigated a range of nine different palladium catalysts in DMF at 80 °C. From our preliminary screen, three catalysts showed greater than 50% starting material consumption (SI Table S3). Most notably, Pd­(PPh_3_)_4_ showed full consumption of starting material and 11% product formation (SI Figures S1–S2). Total consumption of the starting material was indicative of the successful oxidative addition achieved with Pd­(PPh_3_)_4_. Optimizing temperature, base, and solvent conditions for transmetalation improved conversions, with significant increases (45% to 96%) observed upon reducing the temperature (80 to 60 °C) and increasing the number of boronic ester equivalents (5 to 150 equiv) (SI Scheme S1 and SI Table S3).

Despite on-resin peptide synthesis often employing a large excess of reagents to promote those principles outlined by Le Chatelier, we felt the use of such a large excess (150 equiv) of often costly boronic esters was not sufficiently suitable for SPPS.^21^ In order to reduce the required equivalents, we sought to further mitigate dehalogenation, employing 1,4-dioxane in place of DMF.^22^ Furthermore, we found that the relative local concentration of the boronic ester proved to be key to reaction conversion. By reducing solvent volume (from 7 to 2 mL) and employing resins of higher loading (TentaGel-S-NH_2_ [0.23 mmol/g] vs ChemMatrix [0.37 mmol/g] and AminoMethyl polystyrene [0.53 mmol/g]), we were able to significantly reduce boronic ester equivalents (150 to 10 equiv), while maintaining a reaction conversion of 84% (SI Figures S3 and S4, SI Table S4). We then successfully performed the Suzuki–Miyaura coupling of methyl 4-(4,4,5,5-tetramethyl-1,3,2-dioxaborolan-2-yl)­benzoate to on-resin peptide 4* on a 0.1 mmol scale using our standardized conditions (SI Figures S5–S6). Interestingly, the ^1^H NMR spectrum of 5-aryl-1,4-triazole **4** showed the characteristic *cis/trans* isomeric splitting pattern observed in 1,4-triazole **3** (SI Figures S21–S22). Thus, this highlighted that arylation of the triazole removed the aforementioned conformational lock observed with 5-iodo-1,4-triazole **3**.

Confident that on-resin Suzuki–Miyaura coupling to form 4* was sufficiently standardized, we set out to explore the substrate scope of the aryl boronic ester coupling partner (Table 2). The range of substitutions included electron donating (OMe, **5** and **6**) and electron withdrawing (F, **7** and **8**) substituents at the ortho and para positions. Notably, *p*-boronic esters (**5** and **8**) displayed higher conversions than their ortho counterparts (**6** and **8**). Additionally, high product conversion was observed for analogue **9** (which lacked substitution) and biphenyl derivative **10**. Finally, Suzuki–Miyaura cross coupling of 5-iodo-1,4-triazole 3* with 4-aminophenylboronic acid pinacol ester provided peptide **11**, bearing an amine functionality suitable for further functionalization (Table 2, Entry 8).

**Table 2:**
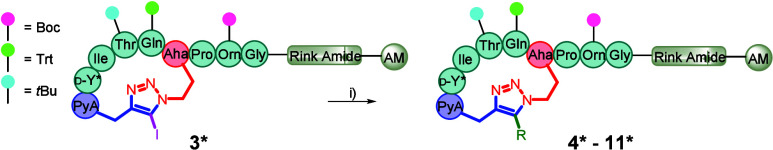

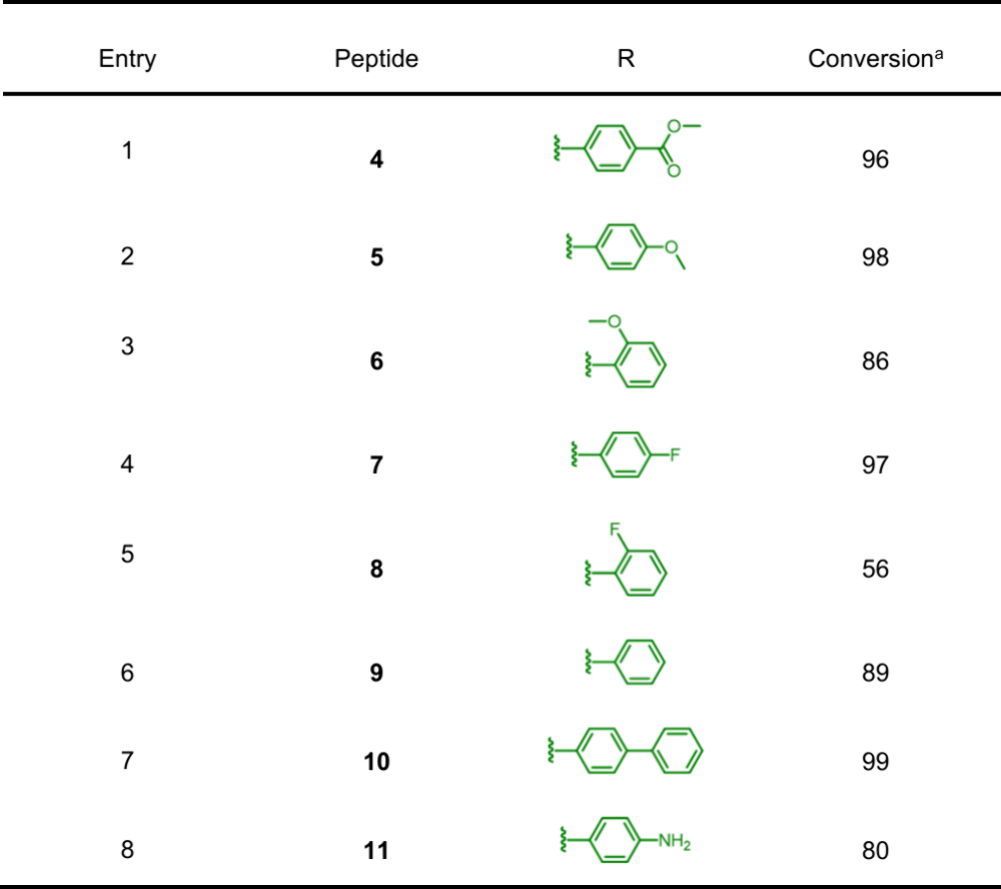
Scope of Aromatic Substitutions for Suzuki–Miyaura Couplings to 5-Iodo-1,4-triazole Peptide 4* ^a^

a % conversion was determined by observation of analytical RP-HPLC at 214 nm as well as LCMS (SI Figures S67–S80).

b Reagents and conditions: (i) boronic acid pinacol ester, K_3_PO_4_, 5% final volume H_2_O, 1,4-dioxane, 60 °C, 18 h, under argon. *: Denotes the on-resin peptide.

Fluorescent labeling is one of the most widely used peptide functionalizations, with tags such as fluorescein isothiocyanate (FITC) typically introduced via flexible N-terminal linkers (e.g., aminohexanoic acid) to prevent thiohydantoin formation and undesired cleavage of the N-terminal residue.^23^ The development of alternative labeling sites with amenable chemistries is therefore desirable. Peptide 11* was therefore functionalized on-resin via direct conjugate addition with FITC, eliminating the need for a linker and affording the more accessible fluorescent peptide **12** (Scheme 2).

**Scheme 2:**
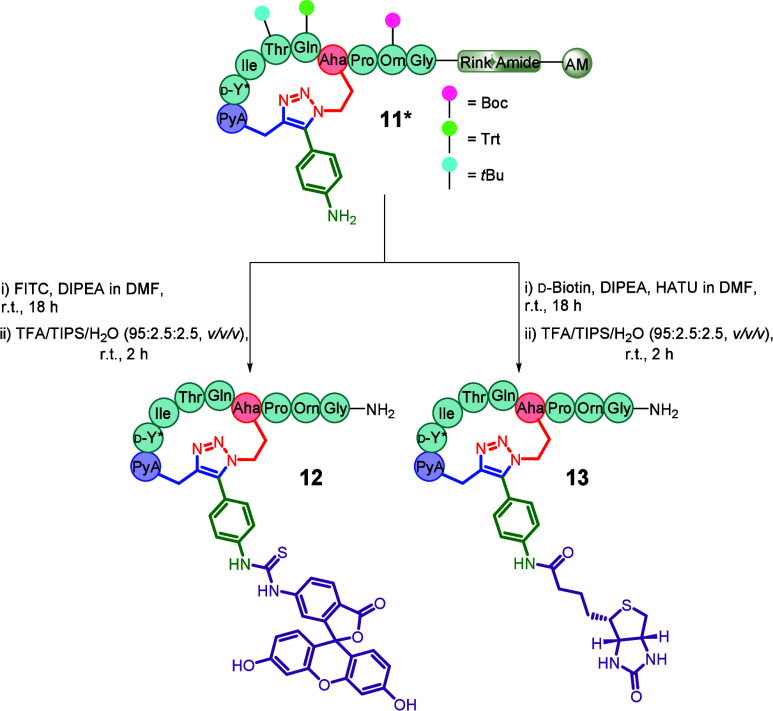
Functionalization of On-Resin Peptide 11* to Yield Biological Tool Compounds **12** and **13**

The biotin–streptavidin interaction is widely employed in protein purification and pull-down assays; therefore, biotinylated analogue **13** was synthesized for these applications (Scheme 2).

In conclusion, we developed a robust on-resin methodology for the synthesis of 5-iodo-1,4-disubstituted-1,2,3-triazole-containing macrocyclic peptides as multifunctional disulfide bridge mimetics. Optimized on-resin 5-iodo-1,4-triazole formation and Suzuki–Miyaura cross-coupling enabled late-stage diversification at the triazole 5-position with a wide range of aryl boronic esters under mild conditions. This strategy was applied to generate novel peptide conjugates bearing functional groups for imaging via a unique bifunctional disulfide bridge mimetic. Notably, structural analysis revealed conformational control imparted by the iodo substituent, which could be modulated through arylation. Together, these findings establish 5-iodo-1,4-triazoles as versatile bioisosteric disulfide bond surrogates with tunable reactivity, opening new avenues for the design of functionalized macrocyclic peptidomimetics in chemical biology and drug discovery.

## Supplementary Material



## Data Availability

The data underlying this study is available in the published article, in its Supporting Information, and openly in DRYAD at 10.5061/dryad.djh9w0wd0.

## Notes

Detailed experimental procedures, characterization, and spectral data for all compounds (PDF)
