# Supplementary material for: On-Resin Synthesis and Late-Stage Functionalization of Macrocyclic Atosiban Mimetics via 5‑Iodo-1,4-triazoles
Source: Org Lett. 2025 Sep 26;27(40):11249–53. doi: 10.1021/acs.orglett.5c03507 (PMC12519475; doi:10.1021/acs.orglett.5c03507)
Supplement: Supplementary file 1 [file ol5c03507_si_001.pdf]

On-resin Synthesis and Late-Stage Functionalization of Macrocyclic Atosiban Mimetics via 5-Iodo-1,4-Triazoles

Oscar A. Shepperson,‡ Michael A. Malone,‡ Kirsty I.M. Arnott, Ryan J. Brown, and Andrew G. Jamieson*.

[‡] These authors contributed equally.

Corresponding Author

***Andrew G. Jamieson** – *School of Chemistry, Advanced Research Centre, University of Glasgow, 11 Chapel Lane, Glasgow, G11 6EW*; orcid.org/0000-0003-1726-7353; Email: Andrew.jamieson.2@glasgow.ac.uk

Authors

Oscar A. Shepperson – *School of Chemistry, Advanced Research Centre, University of Glasgow, 11 Chapel Lane, Glasgow, G11 6EW*; orcid.org/0000-0001-7190-8786
Michael A. Malone – *School of Chemistry, Advanced Research Centre, University of Glasgow, 11 Chapel Lane, Glasgow, G11 6EW*; orcid.org/0009-0007-1476-0659
Kirsty I. M. Arnott – *School of Chemistry, Advanced Research Centre, University of Glasgow, 11 Chapel Lane, Glasgow, G11 6EW*; orcid.org/0009-0004-4527-3683
Ryan J. Brown – *School of Chemistry, Advanced Research Centre, University of Glasgow, 11 Chapel Lane, Glasgow, G11 6EW*; orcid.org/0009-0002-8584-5792

Supporting Information

Contents

Supporting Information....2
General Information and Protocols....4
Reagents and Instrumentation....4
General Protocol 1 for the Automated Fmoc-SPPS....6
General Protocol 2 for On-resin Disubstituted 1,4-triazole Formation by Copper-catalyzed Azide-Alkyne Cycloaddition (CuAAC)....6
General Protocol 3 for Synthesis of 5-Iodo-1,4-Triazole 3* presented in Supporting Information Tables S2.....6
General Protocol 4 for Suzuki Functionalization of 4* presented in Supporting Information Tables S3 – S4....7
General Protocol 5 for Resin Cleavage and Global Deprotection....7
Supporting Information Figures and Schemes....8
Experimental....12
Table of Peptides....12
Peptide Synthesis....13
Synthesis of Disubstituted 1,4-triazole Atosiban (2)....13
Synthesis of Trisubstituted 5-iodo-1,4-triaozle Atosiban (3)....14
Synthesis of Atosiban Suzuki-Miyaura Benzylmethylester Functionalized Peptide (4)....15
Synthesis of Atosiban Suzuki-Miyaura FITC Functionalized peptide (**12**)....16
Synthesis of Atosiban Suzuki-Miyaura Biotin Functionalized peptide (**13**)....17
Characterization....18
Appendix....32
Supporting Information Table S2 of 5-Iodo-1,4-Triazole Forming Reactions....32
HPLC Traces....33
LCMS....37
Supporting Information Tables S3 and S4 of Suzuki-Miyaura Functionalization of 5-Iodo-1,4-Triazole....39
HPLC Traces....41
LCMS....55
Diversification of Aromatic Functionalities....56
HPLC Traces....56
LCMS....60
References....63

General Information and Protocols

Reagents and Instrumentation

All reagents were purchased from commercial sources and used without further purification unless otherwise stated. Standard Fmoc-protected amino acids were purchased from CEM Corporation and Pepceuticals, unless specifically stated differently below. Side chain protecting groups of N^{α}-Fmoc amino acids were as follows; Fmoc-Asn(Trt)-OH (Trt = triphenylmethane), Fmoc-Asp(*t*Bu)-OH (*t*Bu = *tert*-butyl), Fmoc-Arg(Pbf)-OH (Pbf = 2,2,4,6,7-pentamethyldihydrobenzofuran-5- sulfonyl), Fmoc-Cys(Trt), Fmoc-Gln(Trt)-OH, Fmoc-Lys(Boc)-OH (Boc = *tert*-butyloxycarbonyl) and Fmoc-Thr(*t*Bu)-OH.

N,N-Dimethylformamide (DMF) and diethyl ether (Et_2O) were purchased from Rathburn. Triisopropylsilane (TIPS), N-chlorosuccinimide (NCS), [(2-di-*tert*-butylphosphino-2′,4′,6′-triisopropyl-1,1′-biphenyl)-2-(2′-amino-1,1′-biphenyl)] palladium(II) methanesulfonate (*t*BuXPhos), di-*tert*-butyl dicarbonate (Boc_2O) were purchased from Sigma-Aldrich, Merck. *N,N*-diisopropylethylamine (DIPEA), dioxane*,* 1-[Bis(dimethylamino)methylene]-1H-1,2,3-triazolo[4,5-b]pyridinium 3-oxid hexafluorophosphate (HATU), Chloro(2-dicyclohexylphosphino-2′,6′-diisopropoxy-1,1′-biphenyl)[2-(2′-amino-1,1′-biphenyl)] palladium(II) (RuPhos), (2-dicyclohexylphosphino-2′,6′-dimethoxybiphenyl) [2-(2′-amino-1,1′-biphenyl)] palladium(II) methanesulfonate (SPhos), chloro(2-dicyclohexylphosphino-2′,4′,6′-triisopropyl-1,1′-biphenyl)[2-(2′-amino-1,1′-biphenyl)] palladium(II) (XPhos), palladium-tetrakis(triphenylphosphine) ($Pd(PPh_3)_4$), palladium acetate ($Pd(OAc)_2$), palladium(II)bis(triphenylphosphine) dichloride ($PdCl_2(PPh_3)_2$), [1,1′-bis(diphenylphosphino)ferrocene]dichloro-palladium(II) ($Pd(dppf)Cl_2$), [1,1′-bis(diphenylphosphino)ferrocene]dichloro-palladium(II) · dichloromethane (1:1) ($Pd(dppf)Cl_2$ CH_2Cl_2), potassium phosphate trihydrate ($K_3PO_4.3H_2O$), *N,N,N*-tributyl-1-butanaminium bromide (TBAB), Tris[(1-benzyl-1*H*-1,2,3-triazol-4-yl)methyl]amine (TBTA), *N*-iodosuccinimide (NIS), Copper (I) iodide (CuI), methanol-$_{d4}$ (CD_3OD), 2,2,2-trifluoroethanol (TFE), Fmoc-Aha-OH, Fmoc-Orn(Boc)-OH, Fmoc-Tyr(OEt)-OH, Fmoc-Pra-OH, pentynoic acid (PyA), fluorescein-5-isothiocyanate (FITC), Fmoc-D-Tyr(*t*Bu)-OH, margaric acid, D-biotin, Fmoc-β-alanine-OH, dithiothreitol (DTT), glutathione (GSH), iodoacetamide (IA), 3-mercaptopropionic acid (Mpa), and all boronic pinacol esters were purchased from Fluorochem. Morpholine was purchased from Alfa Aesar. Dichloromethane (CH_2Cl_2) was purchased from VWR. Acetonitrile (MeCN), Trifluoroacetic acid (TFA), piperidine and formic acid (FA) were purchased from Fisher Scientific. ChemMatrix© Rink-Amide resin was purchased from Biotage. TentaGel®-S Rink-Amide resin was purchased from Rapp Polymere. Aminomethyl Rink-Amide resin was purchased from Activotec. 3-[2-(2-carboxyethoxy)ethoxy]propanoic acid (Bis-PEG2-acid) and tri-*tert*-butyl 1,4,7,10-*tetra*-azacyclododecane-1,4,7,10-tetraacetate (DOTA) were purchased from AK Scientific.

Dry solvents were purified using a PureSolv 500 MD solvent purification system.

Analytical reverse-phase high-performance liquid chromatography (RP-HPLC) was performed on a Shimadzu RP-HPLC system with Shimadzu LC-20AT pumps, a Shimadzu SIL20A autosampler and a Shimadzu SPD-20A UV-vis detector using a Phenomenex Aeris Peptide XB-C18 (100Å, 5 µm, 150 × 4.6 mm). Compounds were eluted with linear gradients at column-dependent flow rates (1 mL/min for the Aeris), where buffer A = 0.1% TFA in H_2O and buffer B = 0.1% TFA in MeCN. Data is reported as column retention time (t_R) in minutes (mins). Crude peptides were purified by preparative RP-HPLC using an Agilent Technologies 1260 Infinity II Preparative LC System (monitoring at 214 nm and 280 nm), with a Phenomenex Gemini column 5 µm C18 column (100 Å, 250 x 21.2 mm). Peptides were eluted on linear gradients (10 mL/min) as determined by analytical RP-HPLC. The solvents employed were buffer; A = H_2O + 0.1% TFA, and buffer; B = MeCN + 0.1% TFA.

Liquid chromatography-mass spectrometry (LCMS) was performed on a Thermo Scientific LCQ Fleet Ion Trap Mass Spectrometer using positive mode electrospray ionization (ESI^+). Where buffer A = 0.1% TFA in 95% H_2O/5% MeCN and buffer B = 0.1% TFA in 95% MeCN/5% H_2O, a linear gradient of 5% – 95%B over 20 min with a flow rate of 1 mL/min was used with a Phenomenex Aeris Peptide XB-C18 (100 Å, 5 µm, 150 mm x 4.6 mm).

Reactions where heating was required employed a hot plate unless defined otherwise. Where microwave heating was required, it was completed with a CEM Explorer 12 Hybrid Microwave.

Nuclear magnetic resonance experiments were performed with a Bruker AVANCE 400 MHz (^1H) spectrometer. Chemical shifts are recorded in parts per million (ppm) and were referenced to the residual solvent peak for CD_3OD: $\delta = 4.78$ ppm for ^1H spectra.

Infrared (IR) Spectroscopy was recorded using a Shimadzu FTIR-8400S.

General Protocol 1 for the Automated Fmoc-SPPS

Peptides were synthesized batchwise as required on an Automated Biotage Initiator+ Alstra microwave synthesizer on a 0.1 mmol or 0.3 mmol scale. The peptides were synthesized using Rink-Amide functionalized TentaGel-S®-NH_2 (0.23 mmol/g loading, 0.435/1.304 g), ChemMatrix© (0.37 mmol/g loading, 0.270/0.810 g) or Aminomethyl resin (0.53 mmol/g loading, 0.189/0.566 g). Peptides were elongated in cycles of amino acid coupling followed by Fmoc removal. Fmoc-protected amino acid (5 equiv., 0.5 M in DMF) coupling was achieved by treatment with HATU (5 equiv., 0.5 M in DMF) and DIPEA (5 equiv., 0.5 M in DMF) at 75 °C for 10 mins. Fmoc removal was achieved by treatment with 20% morpholine + 5% formic acid in DMF (8 mL, *v/v/v*) at 75 °C for 1 min followed by 75 °C for 5 mins. The resin was washed with DMF following Fmoc removals (4 x 8 mL), and after coupling (2 x 8 mL).

General Protocol 2 for On-resin Disubstituted 1,4-triazole Formation by Copper-catalyzed Azide-Alkyne Cycloaddition (CuAAC)

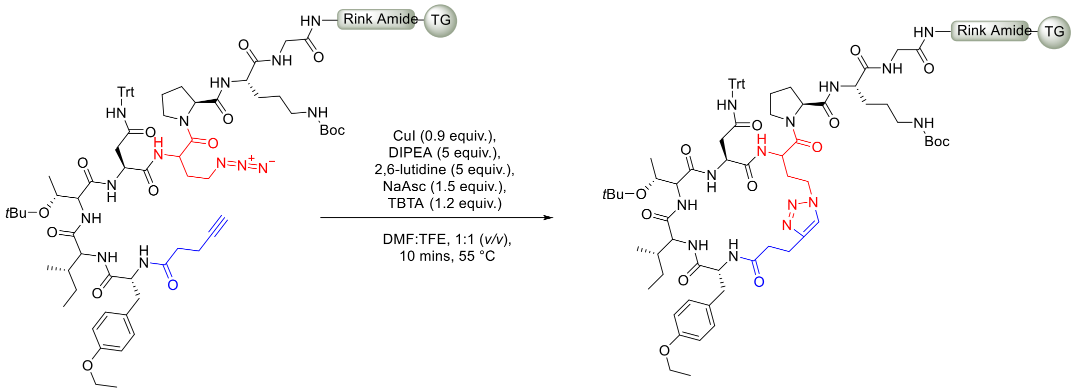

The peptidyl resin (1 equiv.) was swollen in DMF/TFE, 1:1, (*v/v*). To this, copper iodide (0.9 equiv.), DIPEA (5 equiv.), 2,6-lutidine (5 equiv.), NaAsc (1.5 equiv.) and TBTA (1.2 equiv.) were added and the reaction heated (10 mins, 55 °C, µW). Upon reaction completion, the resin was washed with DMF (3 x 5 mL) followed by CH_2Cl_2 (3 x 5 mL) and dried under vacuum.[1]

General Protocol 3 for Synthesis of 5-Iodo-1,4-Triazole 3* presented in Supporting Information Tables S2.

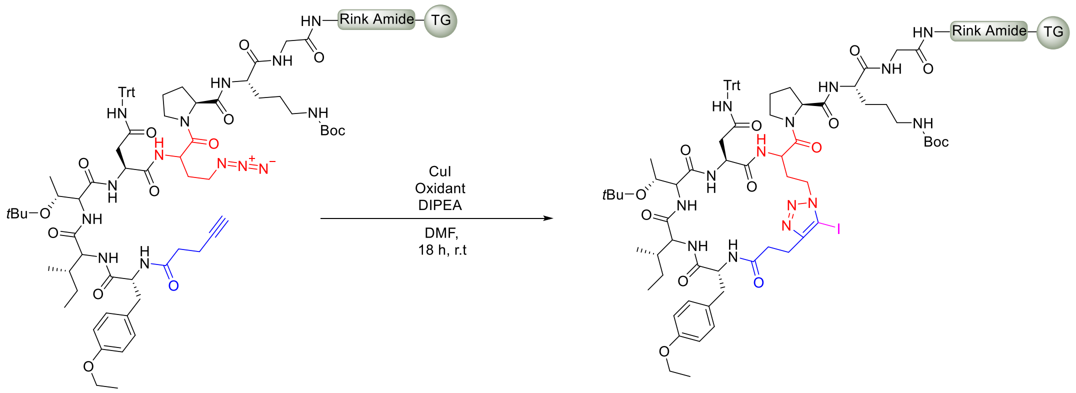

The respective peptidyl resin was swollen in DMF (10 mL final volume per 0.1 mmol resin) with DIPEA for 5 min prior to the addition of any further reagents. CuI in DMF (400 µL) and oxidant in DMF (400 µL) were solubilized separately and combined immediately prior to

addition to the peptidyl resin. The reaction was left, with mixing, overnight at r.t. Upon reaction completion, the peptidyl resin was washed with DMF (3 x 5 mL), 20% piperidine + 5% formic acid in DMF (2 x 5 mL, *v/v/v*), DMF (3 x 5 mL), CH_2Cl_2 (3 x 5 mL), and dried under vacuum.[2] Reaction progression was monitored by cleavage of a small portion of the peptidyl resin and analyzed by RP-HPLC and LCMS. Specific reagents and modified molar equivalents are specified in **Supporting Information Table S2**. The major products identified during the optimization process were; the linear peptide (**1**), the disubstituted 1,4-triazole (**2**), and the trisubstituted 5-iodo-1,4-triazole (**3**).[3]

General Protocol 4 for Suzuki Functionalization of 4* presented in Supporting Information Tables S3 – S4

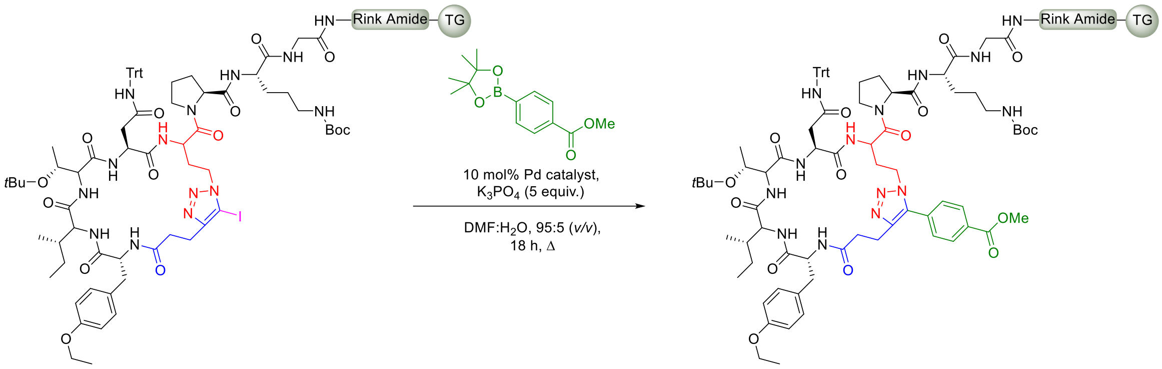

The respective peptidyl resin was swollen in either DMF or 1,4-dioxane (5 mL per 0.1 mmol resin) for 5 minutes prior to the addition of any further reagents. Following resin swelling, $K_3PO_4.3H_2O$ (5 equiv.) in H_2O (5% *v/v* of total final volume) and 4-methoxycarbonylphenylboronic acid pinacol ester (**Supporting Information Tables S3 and S4)** in either DMF or 1,4-dioxane (0.1 – 2 mL) were added to the solution. The resin containing solution was then degassed with argon (10 mins) prior to reaction initiation by addition of the respective Pd cat. (10 mol%) at 60 °C for 18 hours. Upon reaction completion, the peptidyl resin was washed with DMF (3 x 5 mL), 20% piperidine + 5% formic acid in DMF (2 x 5 mL, *v/v/v*), DMF (3 x 5 mL), CH_2Cl_2 (3 x 5 mL), and dried under vacuum. Reaction progression was monitored by cleavage of a small portion of the peptidyl resin and analyzed by RP-HPLC and LCMS. Specific reagents and modified molar equivalents are specified in the article (**Supporting Information Tables S3 and S4**). The major products identified during the optimization process were; the trisubstituted 5-iodo-1,4-triazole (**3**), the trisubstituted 5-4-methoxycarbonylphenyl-1,4-triazole (**4**) and, the disubstituted 1,4-triazole (**2**).

General Protocol 5 for Resin Cleavage and Global Deprotection

The resin-bound peptide was treated with a cleavage cocktail of TFA/H_2O/TIPS (95/2.5/2.5, *v/v/v*, 10 mL per 0.1 mmol resin) and agitated (1 – 2 h, r.t.). The cleavage solution was separated from the resin and its volume reduced under a flow of N_2. Ice cold Et_2O (15 mL per 0.1 mmol resin) was used to precipitate the peptide. The precipitate was isolated by centrifugation, washed once more with ice cold Et_2O, dissolved in MeCN/MQ H_2O (2:8, *v/v*) and analyzed by RP-HPLC and LCMS. Following determination of the desired peptidyl products, the solutions were frozen and lyophilized.

Supporting Information Figures and Schemes

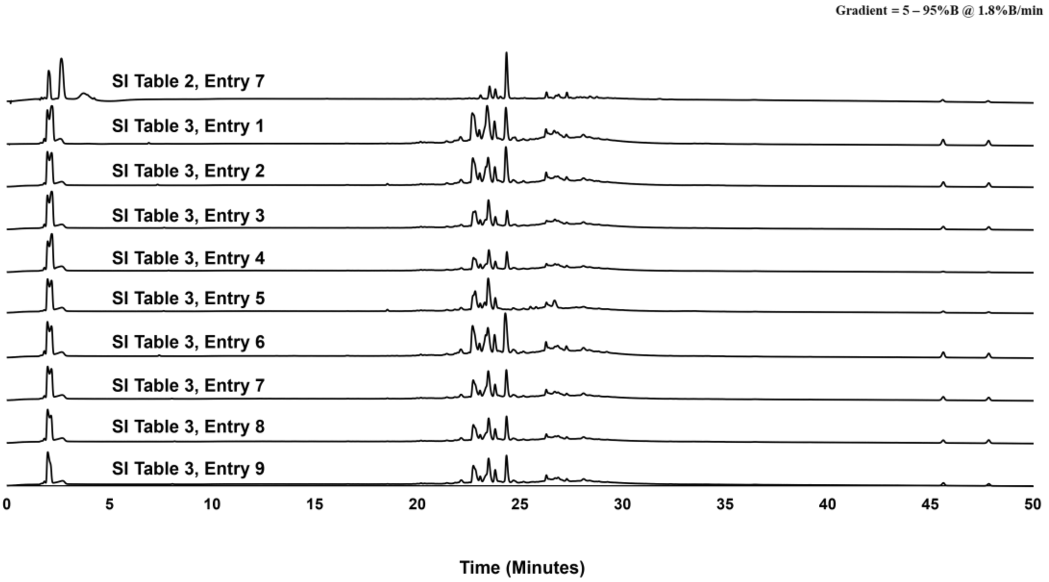

Supporting Information Figure S1. Analytical RP-HPLC chromatograms (214 nm) of catalyst screen (**Supporting Information Table S3**, entries 1 – 9). Aeris Peptide XB-C18 (100 Å, 5 µm, 150 mm x 4.6 mm), linear gradient 5% – 95%B over 50 min (*ca.* 1.8%B/min) at 1 mL/min.

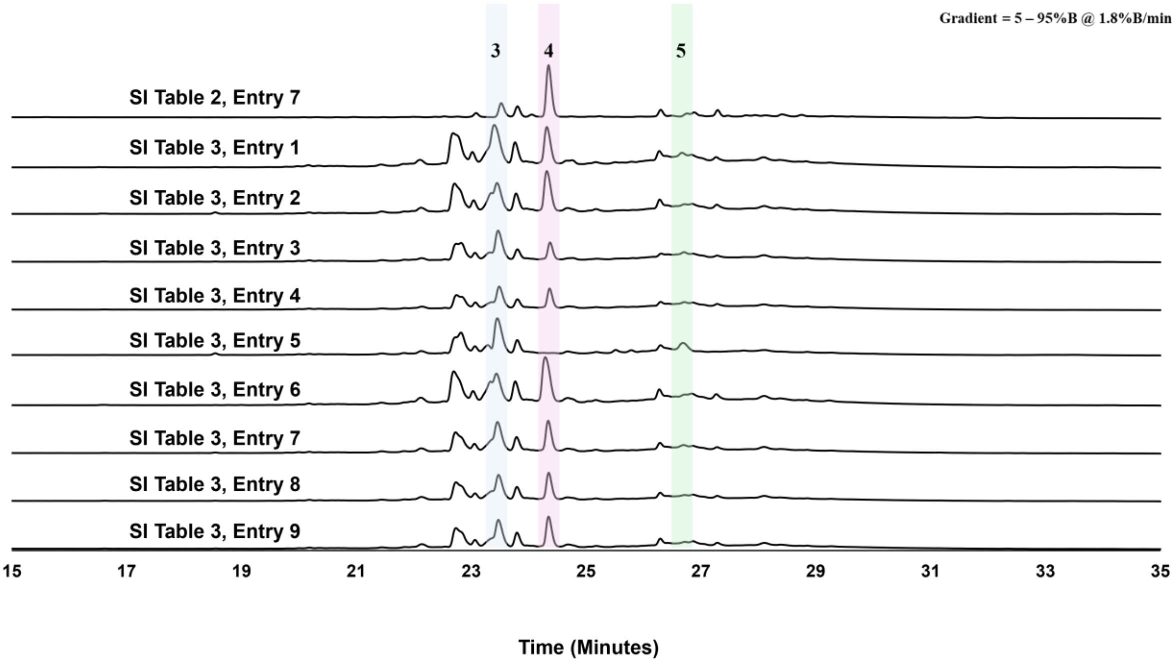

Supporting Information Figure S2. Zoomed analytical RP-HPLC chromatograms (214 nm) of catalyst screen (**Supporting Information Table S3**, entries 1 – 9). Aeris Peptide XB-C18 (100 Å, 5 µm, 150 mm x 4.6 mm), linear gradient 5% – 95%B over 50 min (*ca.* 1.8%B/min) at 1 mL/min.

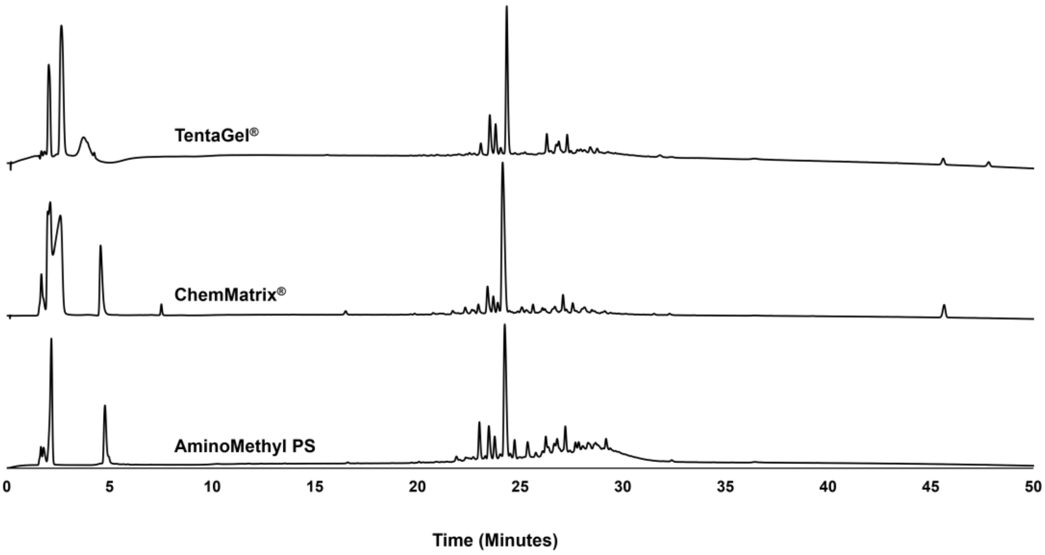

Supporting Information Figure S3. Analytical RP-HPLC chromatograms (214 nm) of variable resins employed for the linear synthesis and cyclisation to yield **3**. Aeris Peptide XB-C18 (100 Å, 5 µm, 150 mm x 4.6 mm), linear gradient 5% – 95%B over 50 min (*ca.* 1.8%B/min) at 1 mL/min.

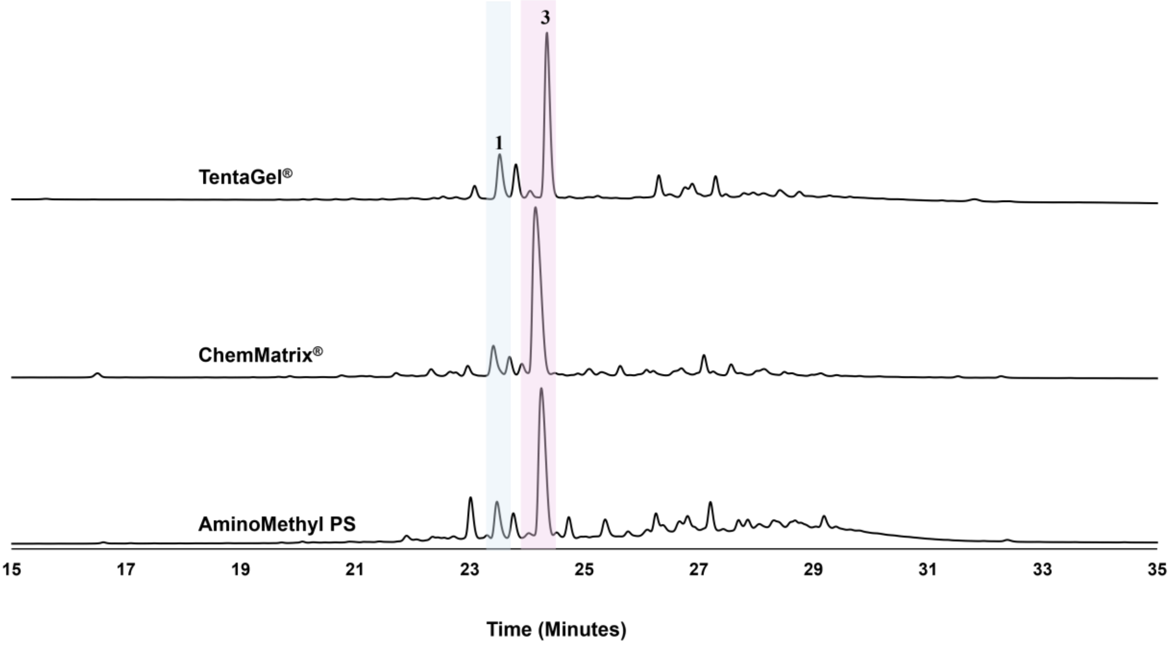

Supporting Information Figure S4. Zoomed analytical RP-HPLC chromatograms (214 nm) of variable resins employed for the linear synthesis and cyclisation to yield **3**. Aeris Peptide XB-C18 (100 Å, 5 µm, 150 mm x 4.6 mm), linear gradient 5% – 95%B over 50 min (*ca.* 1.8%B/min) at 1 mL/min.

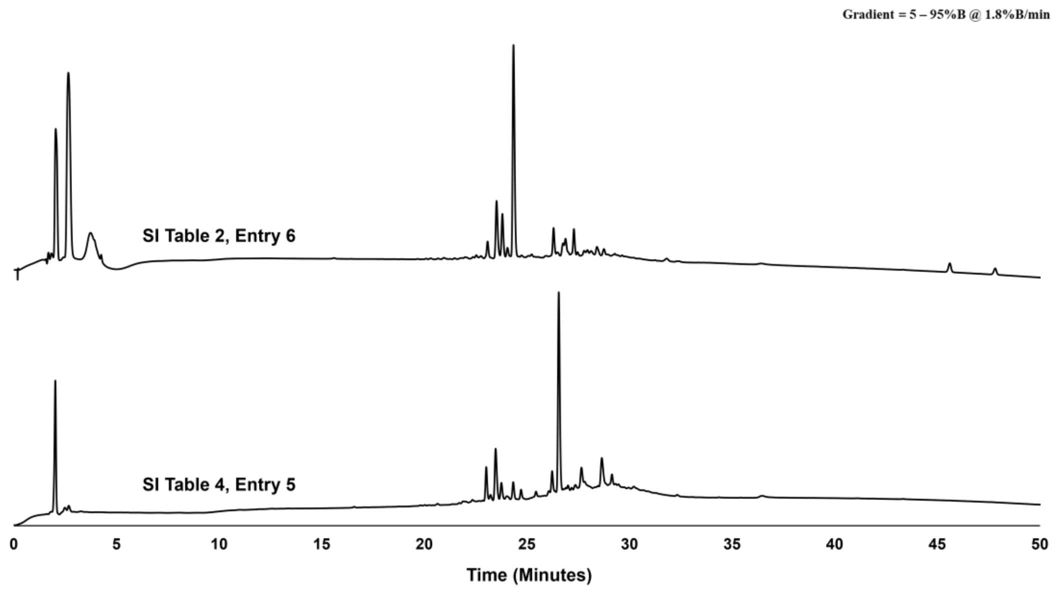

Supporting Information Figure S5. Analytical RP-HPLC chromatograms (214 nm) of optimized Suzuki conditions. Aeris Peptide XB-C18 (100 Å, 5 µm, 150 mm x 4.6 mm), linear gradient 5% – 95%B over 50 min (*ca.* 1.8%B/min) at 1 mL/min.

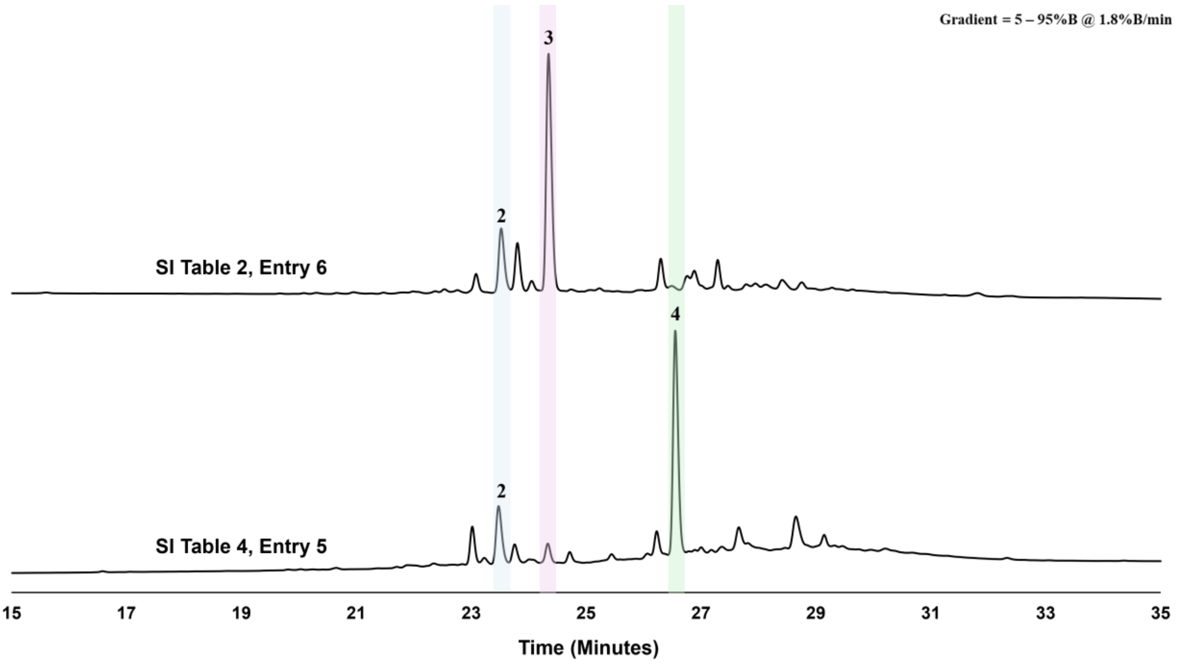

Supporting Information Figure S6. Zoomed analytical RP-HPLC chromatograms (214 nm) of optimized Suzuki conditions. Aeris Peptide XB-C18 (100 Å, 5 µm, 150 mm x 4.6 mm), linear gradient 5% – 95%B over 50 min (*ca.* 1.8%B/min) at 1 mL/min.

Supporting Information Scheme S1. Summary of the development of conditions to perform a Suzuki-Miyaura coupling to the on-resin 5-iodo-1,4-triazole containing macrocyclic peptide **4***. Key factors influencing reaction success and avoiding dehalogenation are highlighted at relevant points of the reaction cycle. *denotes the on-resin peptide.

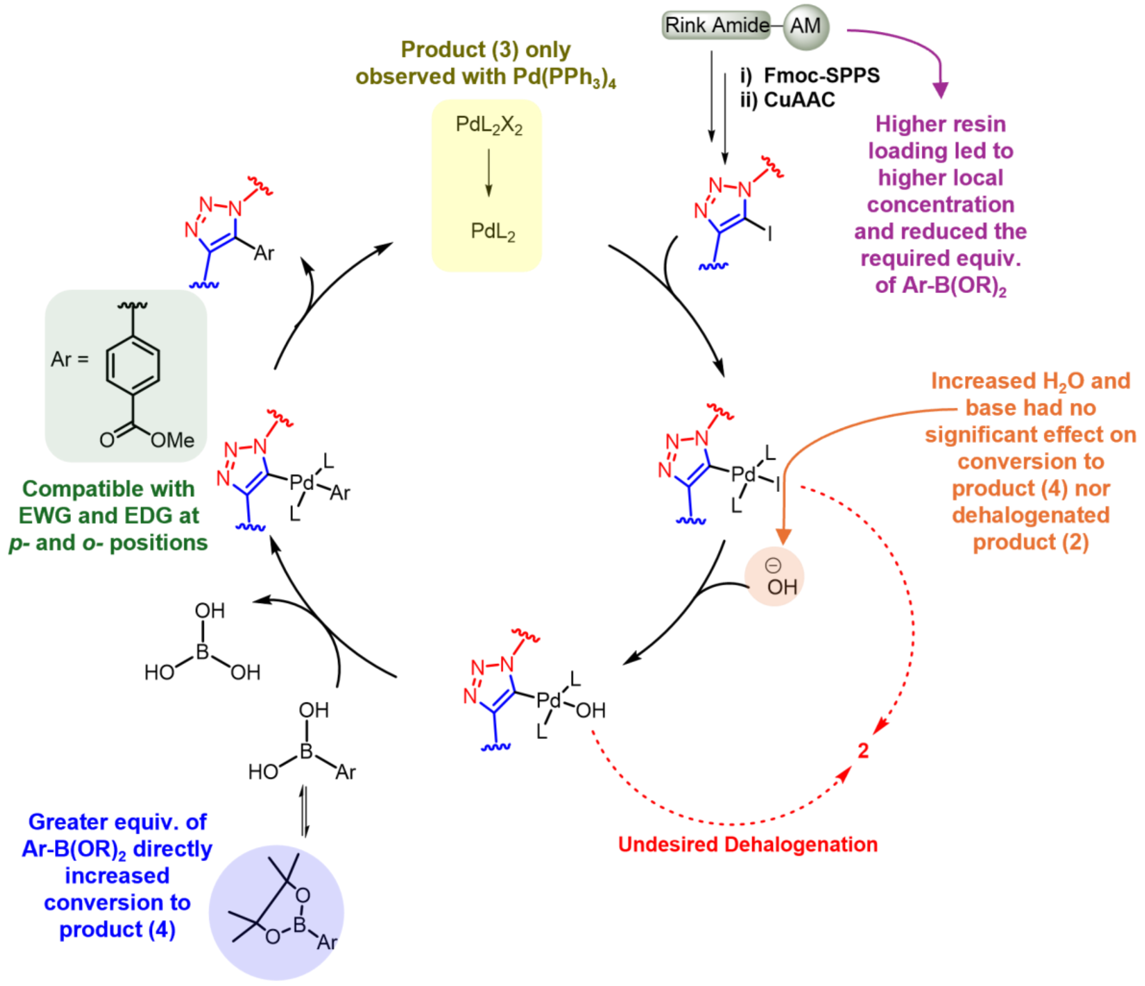

Experimental

Table of Peptides

Supporting Information Table S1. Name, sequence, % yield, % purity, *m/z* and retention time of peptides. Abbreviations: Aha =Azidohomoalanine, Mpa = 3-Mercaptopropionic acid, NH_2 = C-terminal amide, Orn = Ornithine, PyA = Pentynoic Acid and y-(OEt) = D-Tyrosine(OEt). Detailed characterization data for peptides can be found in **Supporting Information Figures S7 – S25**.

Peptide	Sequence	Bridge – 5'-Functionality	Yield (%)	% Purity	Calculated *m/z*	Observed *m/z*	t_R (mins)
**2**	*(PyA)(y-OEt)ITN(Aha)P(Orn)G-NH$_2$*	Triazole – H	9	98	1011.15	1010.77 ± 0.45	23.1
**3**	*(PyA)(y-OEt)ITN(Aha)P(Orn)G-NH$_2$*	Triazole – I	7	98	1137.05	1136.81 ± 0.19	24.0
**4**	*(PyA)(y-OEt)ITN(Aha)P(Orn)G-NH$_2$*	Triazole – Benzylmethylester	4	98	1145.29	1145.57 ± 0.50	25.6
**12**	*(PyA)(y-OEt)ITN(Aha)P(Orn)G-NH$_2$*	Triazole – Aniline – FITC	3	97	1491.65	1491.66 ± 0.20	28.8
**13**	*(PyA)(y-OEt)ITN(Aha)P(Orn)G-NH$_2$*	Triazole – Aniline – Biotin	2	95	1328.56	1328.68 ± 0.37	25.6

Peptide Synthesis

Synthesis of Disubstituted 1,4-triazole Atosiban (2)

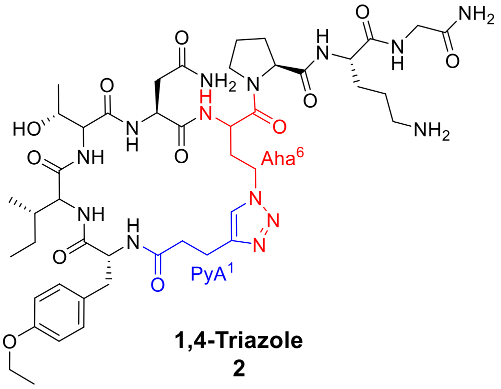

1,4-Triazole
2

The linear precursor for peptide **1** was synthesized by automated Fmoc-SPPS as described in **General Protocol 1** (0.1 mmol) with PyA and Aha incorporated at positions 1 and 6 respectively. Following synthesis of the linear peptide, the peptidyl resin was subjected to disubstituted copper-catalyzed azide-alkyne cycloaddition (CuAAC) conditions, as described in **General Protocol 2**. The final cyclized peptide was liberated from the resin and its protecting groups simultaneously removed under the conditions described in **General Protocol 5** to afford the crude cyclized peptide, **2** (90 mg, 90% yield [based on initial resin loading]) at approximately 59% purity.

Crude peptide **2** (90 mg), was solubilized in a solution of 0.1% TFA in MeCN:MQ H_2O (2:8; *v/v*) at a concentration of ~45 mg/mL and purified by preparative RP-HPLC (1 x 2000 µL injection) employing a gradient of 15% – 60%B over 45 min (*ca.* 1%B/min) at a flow rate of 10 mL/min. Fractions were analyzed by RP-HPLC and LCMS for compound identification and lyophilized to afford the compound, **2**, as a white amorphous powder (9.4 mg, 10% recovery [based on crude yield], 98% purity, 9% overall yield).

LCMS: Mass calculated for [$C_{46}H_{70}N_{14}O_{12}$ + H] 1011.15; deconvoluted mass observed 1010.77 ± 0.45. Charge states; 506.54 $[M+2H]^{2+}$, 1011.45 $[M+H]^{+}$.

RP-HPLC: t_R = 23.1 min. Phenomenex Aeris Peptide XB-C18 (100 Å, 5 µm, 150 mm x 4.6 mm), linear gradient 5% – 95%B over 60 min (*ca.* 1.8%B/min) at 1 mL/min.

IR ν_{max} (neat): 3357, 2921, 2851, 1658, 1632, 1469, 1411, 706 cm^{-1}.

Synthesis of Trisubstituted 5-iodo-1,4-triaozle Atosiban (3)

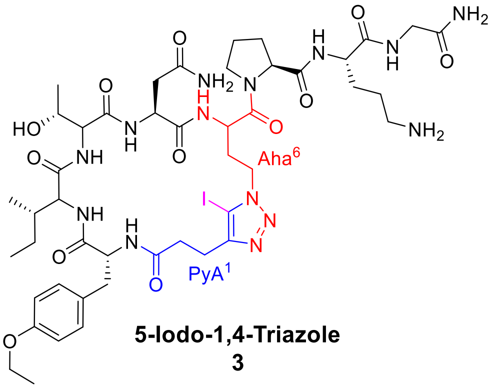

5-Iodo-1,4-Triazole
3

The linear precursor for peptide **1** was synthesized by automated Fmoc-SPPS as described in **General Protocol 1** (0.1 mmol) with PyA and Aha incorporated at positions 1 and 6 respectively. Following synthesis of the linear peptide, the peptidyl resin was subjected to trisubstituted copper-catalyzed azide-alkyne cycloaddition (CuAAC) conditions, as described in **General Protocol 3** (**Supporting Information Table S2**, entry 6). The final cyclized peptide was liberated from the resin and its protecting groups removed simultaneously under the conditions described in **General Protocol 5** to afford the crude cyclized peptide, **3** (45 mg, 40% yield [based on initial resin loading]) at approximately 64% purity.

Crude peptide (**3**) (45 mg), was solubilized in a solution of 0.1% TFA in MeCN:MQ H_2O (2:8, *v/v*) at a concentration of 22.5 mg/mL and purified by preparative RP-HPLC (1 x 2000 µL injection) employing a gradient of 15% – 60%B over 45 min (*ca.* 1%B/min) at a flow rate of 10 mL/min. Fractions were analyzed by RP-HPLC and LCMS for compound identification and lyophilized to afford the compound, **3**, as a white amorphous powder (8.3 mg, 18% recovery [based on crude yield], 98% purity, 7% overall yield).

LCMS: Mass calculated for [$C_{46}H_{69}IN_{14}O_{12}$ + H] 1137.05; deconvoluted mass observed 1136.81 ± 0.19. Charge states; 569.47 $[M+2H]^{2+}$, 1137.67 $[M+H]^{+}$.

RP-HPLC: t_R = 24.0 min. Phenomenex Aeris Peptide XB-C18 (100 Å, 5 µm, 150 mm x 4.6 mm), linear gradient 5% – 95%B over 60 min (*ca.* 1.8%B/min) at 1 mL/min.

IR ν_{max} (neat): 3360, 2980, 2922, 1632, 1383, 1252, 1151, 952 cm^{-1}.

Synthesis of Atosiban Suzuki-Miyaura Benzylmethylester Functionalized Peptide (4)

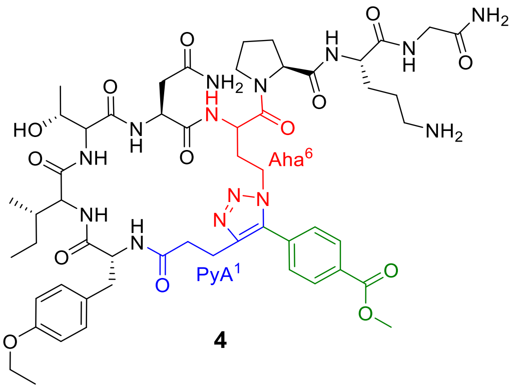

4

The linear precursor for peptide **1** was synthesized by automated Fmoc-SPPS as described in **General Protocol 1** (0.1 mmol) with PyA and Aha incorporated at positions 1 and 6 respectively. Following synthesis of the linear peptide, the peptidyl resin was subjected to trisubstituted copper-catalyzed azide-alkyne cycloaddition (CuAAC) conditions, as described in **General Protocol 3** (**Supporting Information Table S2**, entry 6), following which the resin underwent standardized Suzuki-Miyaura conditions as described in **General Protocol 4** (**Supporting Information Table S4**, Entry 5). The final cyclized peptide was liberated from the resin and its protecting groups removed simultaneously under the conditions described in **General Protocol 5** to afford the crude cyclized peptide, **4** (103 mg, 90% yield [based on initial resin loading]) at approximately 50% purity.

Crude peptide (**4**) (103 mg), was solubilized in a solution of 0.1% TFA in MeCN:MQ H_2O (2:8, *v/v*) at a concentration of ~25 mg/mL and purified by preparative RP-HPLC (2 x 2000 µL injection) employing a gradient of 15% – 60%B over 45 min (*ca.* 1%B/min) at a flow rate of 10 mL/min. Fractions were analyzed by RP-HPLC and LCMS for compound identification and lyophilized to afford the compound, **4**, as a white amorphous powder (4.5 mg, 4% recovery [based on crude yield], 98% purity, 4% overall yield).

LCMS: Mass calculated for [$C_{54}H_{76}N_{14}O_{14}$ + H] 1145.29; deconvoluted mass observed 1145.57 ± 0.50. Charge states; 573.98 $[M+2H]^{2+}$, 1146.22 $[M+H]^{+}$.

RP-HPLC: t_R = 25.6 min. Phenomenex Aeris Peptide XB-C18 (100 Å, 5 µm, 150 mm x 4.6 mm), linear gradient 5% – 95%B over 60 min (*ca.* 1.8%B/min) at 1 mL/min.

Synthesis of Atosiban Suzuki-Miyaura FITC Functionalized peptide (**12**)

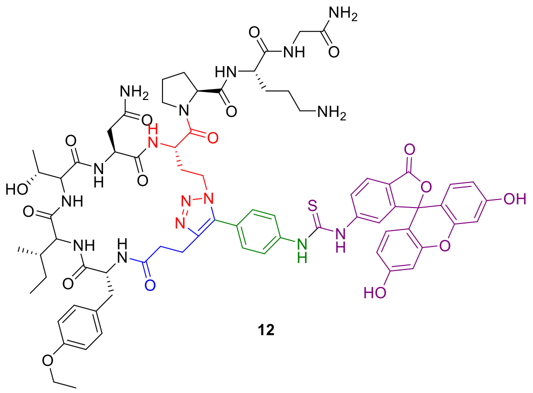

12

The linear precursor for peptide **1** was synthesized by automated Fmoc-SPPS as described in **General Protocol 1** (0.1 mmol). Following resin elongation, the peptide underwent a copper mediated reaction as reported in **General Protocol 3** (**Supporting Information Table S2**, entry 6). Following capping the peptidyl resin underwent a Pd catalyzed Suzuki-Miyaura reaction as detailed by **General Protocol 4** (**Supporting Information Table S4**, Entry 5). on-resin with FITC (116.7 mg, 0.3 mmol, 3 equiv.), DIPEA (104.5 µL, 0.6 mmol, 6 equiv.) and DMF (5 mL for 0.1 mmol of resin) at room temperature overnight excluding light. Following reaction completion, the peptidyl resin was washed with DMF (2 x 5 mL), CH_2Cl_2 (3 x 5 mL), and dried under vacuum. The final cyclized peptide was liberated from the resin and its protecting groups simultaneously removed under the conditions described in **General Protocol 5** to afford the crude cyclized peptide, **12** (109 mg, 73% yield [based on initial resin loading]) at approximately 45% purity.

Crude peptide **12** (109 mg), was solubilized in 0.1% TFA in MeCN:MQ H_2O (2:8, *v/v*) at a concentration of ~28 mg/mL and purified by preparative RP-HPLC (2 x 2500 µL injection) employing a gradient of 15% – 60%B over 45 min (*ca.* 1%B/min) at a flow rate of 10 mL/min. Fractions were analyzed by RP-HPLC and LCMS for compound identification and lyophilized to afford the compound, **12**, as a yellow amorphous powder (4.1 mg, 4% recovery [based on crude yield], 97% purity, 3% overall yield).

LCMS: Mass calculated for [$C_{73}H_{86}N_{16}O_{17}S$ + H] 1491.65; deconvoluted mass observed: 1491.66 ± 0.20. Charge states; 746.90 $[M+2H]^{2+}$, 1492.52 $[M+H]^{+}$.

RP-HPLC: t_R = 28.8 min. Phenomenex Aeris Peptide XB-C18 (100 Å, 5 µm, 150 mm x 4.6 mm), linear gradient 5% – 95%B over 50 min (*ca.* 1.8%B/min) at 1 mL/min.

Synthesis of Atosiban Suzuki-Miyaura Biotin Functionalized peptide (**13**)

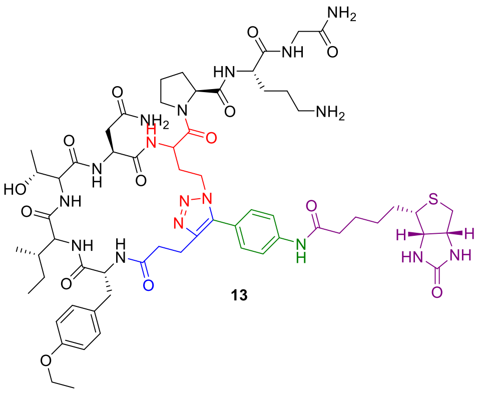

13

The linear precursor for peptide **1** was synthesized by automated Fmoc-SPPS as described in **General Protocol 1** (0.15 mmol). Following resin elongation, the peptide underwent a copper mediated reaction as reported in **General Protocol 3** (**Supporting Information Table S2**, entry 6). Following capping the peptidyl resin underwent a Pd catalyzed Suzuki-Miyaura reaction as detailed by **General Protocol 4** (**Supporting Information Table S4**, Entry 5). To the resultant peptide was coupled D-biotin (97.7 mg, 0.4 mmol, 4 equiv.) in the presence of HATU (144.4 mg, 0.38 mmol, 3.8 equiv.), and DIPEA (174.8 µL, 1.0 mmol, 10 equiv.) in DMF at r.t. for 18 hours. The resin was washed with DMF (3 x 5 mL) and CH_2Cl_2 (3 x 5 mL) and dried under vacuum. The final cyclized peptide was liberated from the resin and its protecting groups simultaneously removed under the conditions described in **General Protocol 5** to afford the crude cyclized peptide, **13** (138 mg, 69% yield [based on initial resin loading]) at approximately 47% purity.

Crude peptide **13** (138 mg), was solubilized in 0.1% TFA in MeCN:MQ H_2O (2:8, *v/v*) at a concentration of ~35 mg/mL and purified by preparative RP-HPLC (2 x 2000 µL injection) employing a gradient of 15% – 60% B over 45 min (*ca.* 1%B/min) at a flow rate of 10 mL/min. Fractions were analyzed by RP-HPLC and LCMS for compound identification and lyophilized to afford the compound, **13**, as a yellow amorphous powder (4.5 mg, 3% recovery [based on crude yield], 95% purity, 2% overall yield).

LCMS: Mass calculated for [$C_{62}H_{89}N_{17}O_{14}S$ + H] 1328.56 deconvoluted mass observed: 1328.68 ± 0.37. Charge states; 665.46 $[M+2H]^{2+}$, 1329.42 $[M+H]^{+}$.

RP-HPLC: t_R = 25.6 min. Phenomenex Aeris Peptide XB-C18 (100 Å, 5 µm, 150 mm x 4.6 mm), linear gradient 5% – 95%B over 50 min (*ca.* 1.8%B/min) at 1 mL/min.

Characterization

Reverse Phase – High Performance Liquid Chromatography (RP-HPLC)

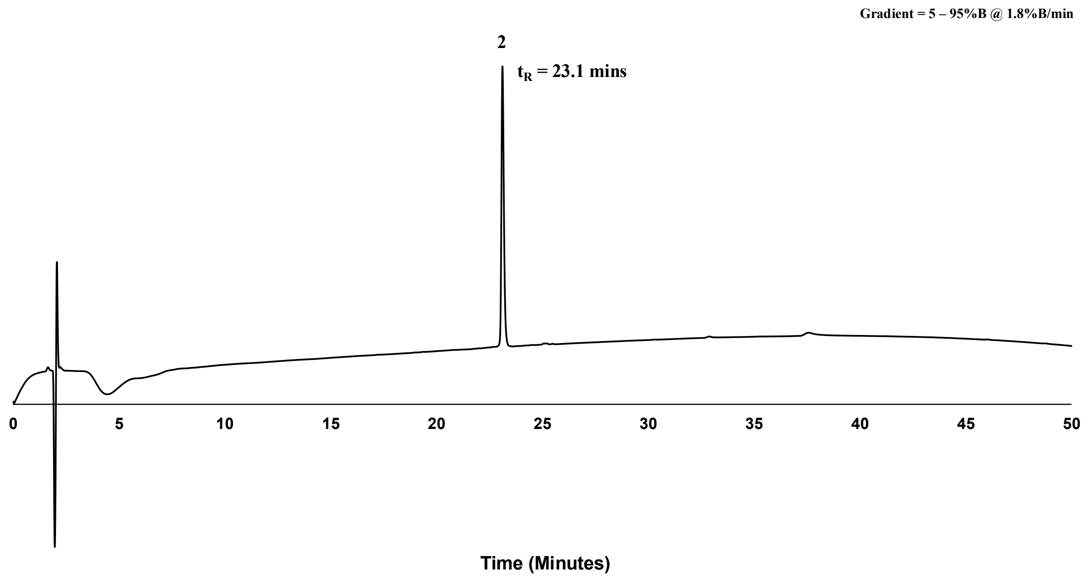

Supporting Information Figure S7. Analytical RP-HPLC chromatogram (214 nm) of purified peptide, **2** (*ca.* 98% as analyzed by peak area. Phenomenex Aeris Peptide XB-C18 (100 Å, 5 µm, 150 mm x 4.6 mm), linear gradient 5% – 95%B over 50 min (*ca.* 1.8%B/min) at 1 mL/min. t_R = 23.1 mins.

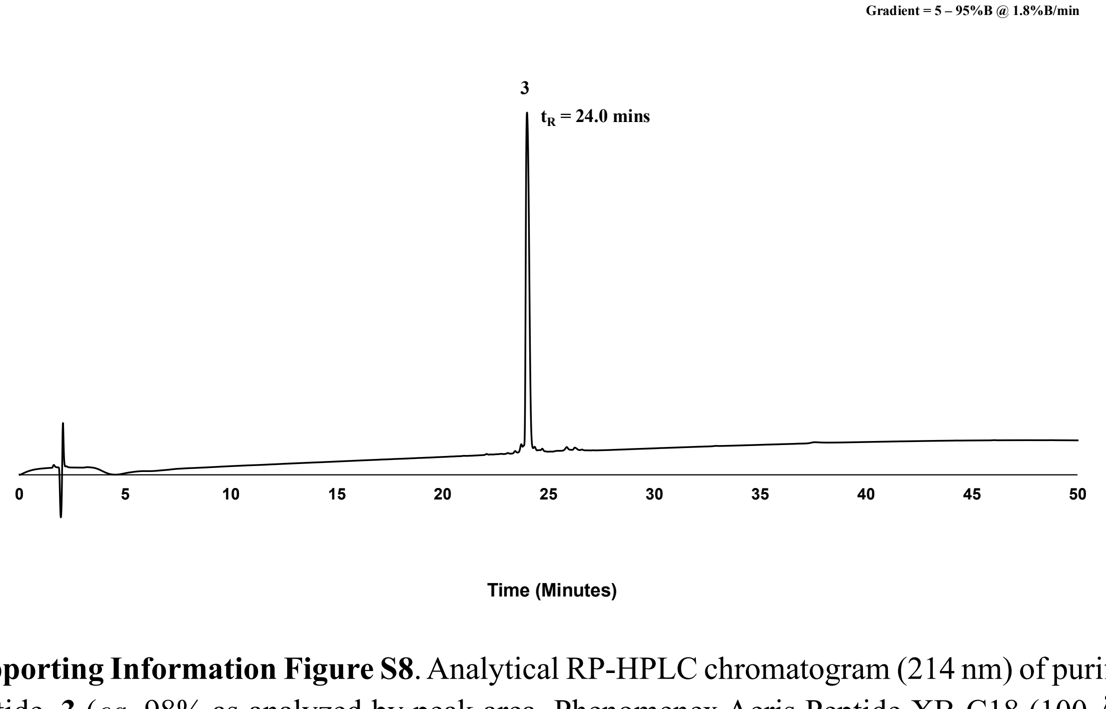

Supporting Information Figure S8. Analytical RP-HPLC chromatogram (214 nm) of purified peptide, **3** (*ca.* 98% as analyzed by peak area. Phenomenex Aeris Peptide XB-C18 (100 Å, 5 µm, 150 mm x 4.6 mm), linear gradient 5% – 95%B over 50 min (*ca.* 1.8%B/min) at 1 mL/min. t_R = 24.0 mins.

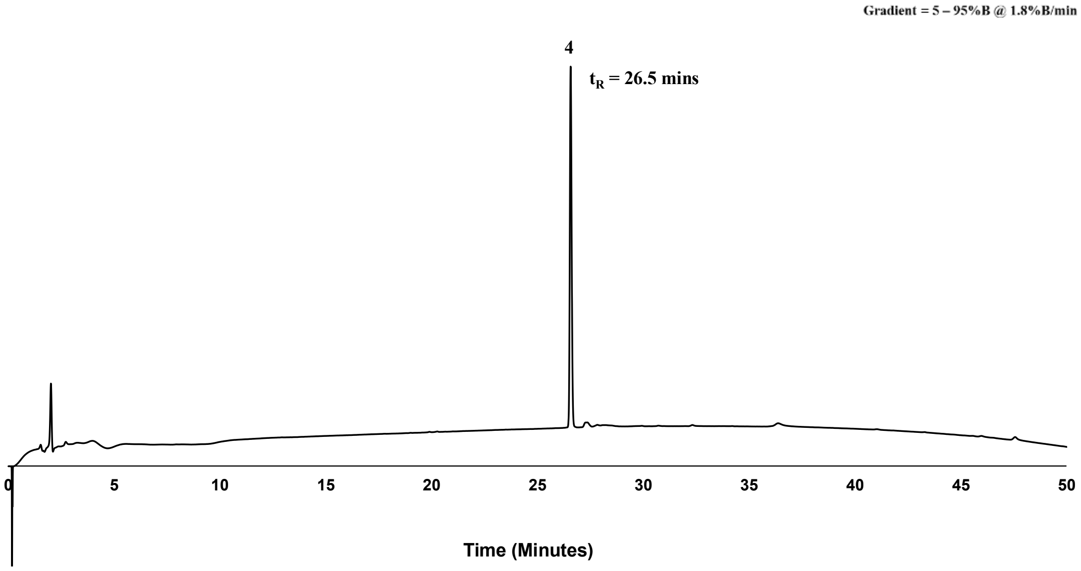

Supporting Information Figure S9. Analytical RP-HPLC chromatogram (214 nm) of purified peptide, **4** (*ca.* 98% as analyzed by peak area. Phenomenex Aeris Peptide XB-C18 (100 Å, 5 µm, 150 mm x 4.6 mm), linear gradient 5% – 95%B over 50 min (*ca.* 1.8%B/min) at 1 mL/min. t_R = 25.6 mins.

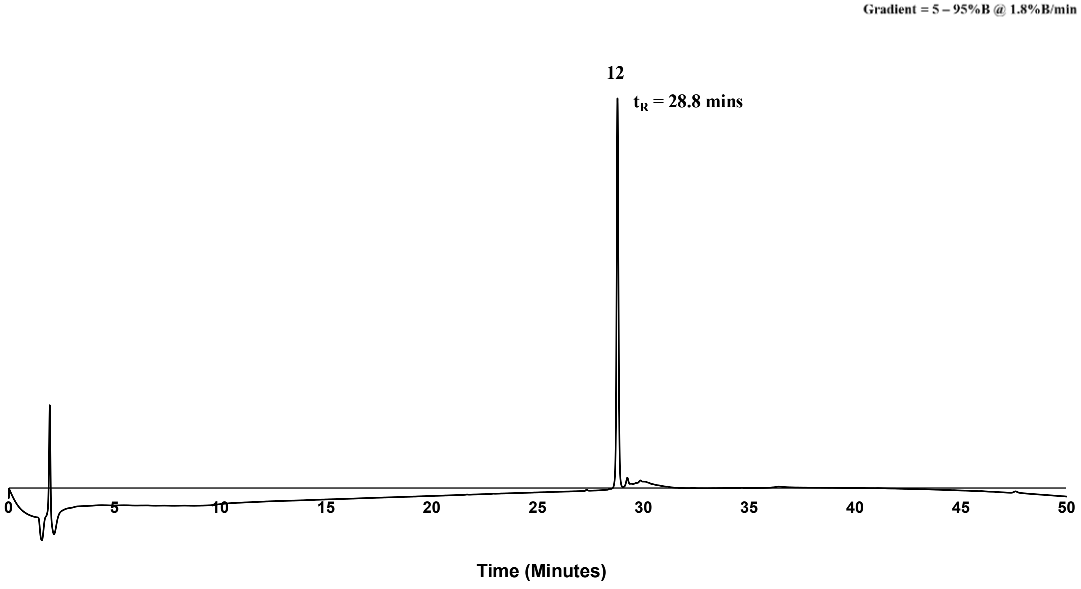

Supporting Information Figure S10. Analytical RP-HPLC chromatogram (214 nm) of purified peptide, **12** (*ca.* 97% as analyzed by peak area. Phenomenex Aeris Peptide XB-C18 (100 Å, 5 µm, 150 mm x 4.6 mm), linear gradient 5% – 95%B over 50 min (*ca.* 1.8%B/min) at 1 mL/min. t_R = 28.8 mins.

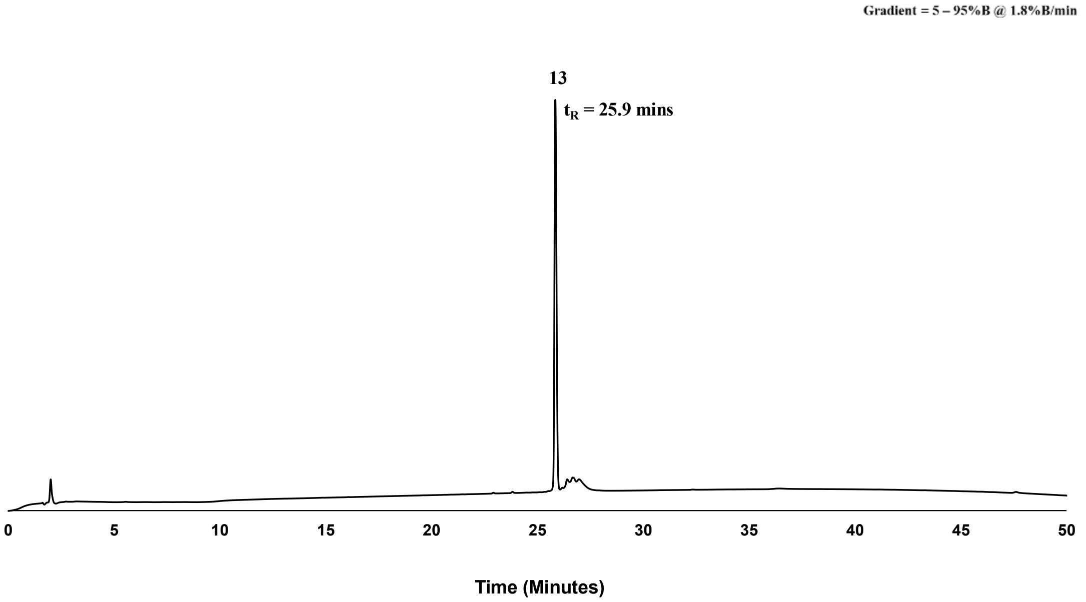

Supporting Information Figure S11. Analytical RP-HPLC chromatogram (214 nm) of purified peptide, 18 (*ca.* 95% as analyzed by peak area. Phenomenex Aeris Peptide XB-C18 (100 Å, 5 µm, 150 mm x 4.6 mm), linear gradient 5% – 95%B over 50 min (*ca.* 1.8%B/min) at 1 mL/min. t_R = 25.9 mins.

Liquid Chromatography Mass Spectrometry (LCMS)

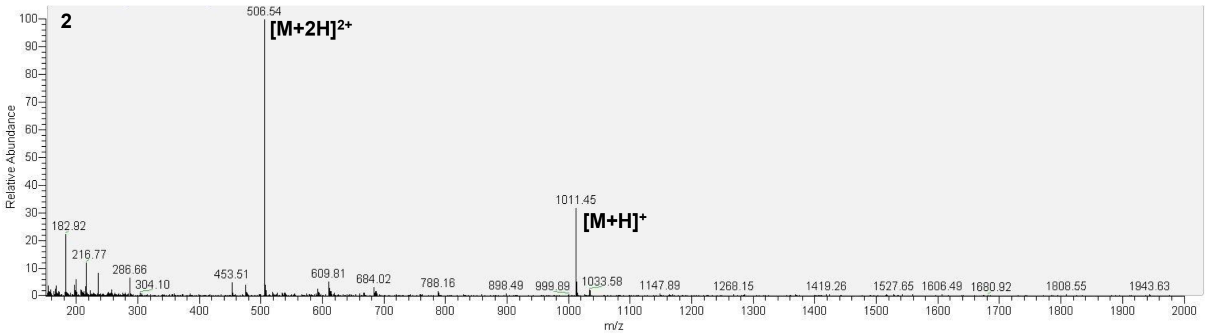

Supporting Information Figure S12. LCMS of purified peptide, **2**, mass calculated for [$C_{46}H_{70}N_{14}O_{12}$ + H] 1011.15; deconvoluted mass observed 1010.77 ± 0.45. Charge states; 506.54 $[M+2H]^{2+}$, 1011.45 $[M+H]^{+}$.

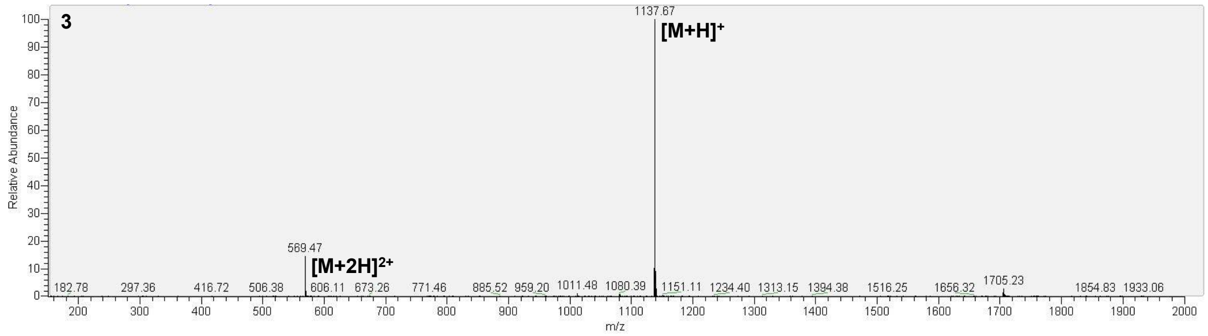

Supporting Information Figure S13. LCMS of purified peptide, **3**, mass calculated for [$C_{46}H_{69}IN_{14}O_{12}$ + H] 1137.05; deconvoluted mass observed 1136.81 ± 0.19. Charge states; 569.47 $[M+2H]^{2+}$, 1137.67 $[M+H]^{+}$.

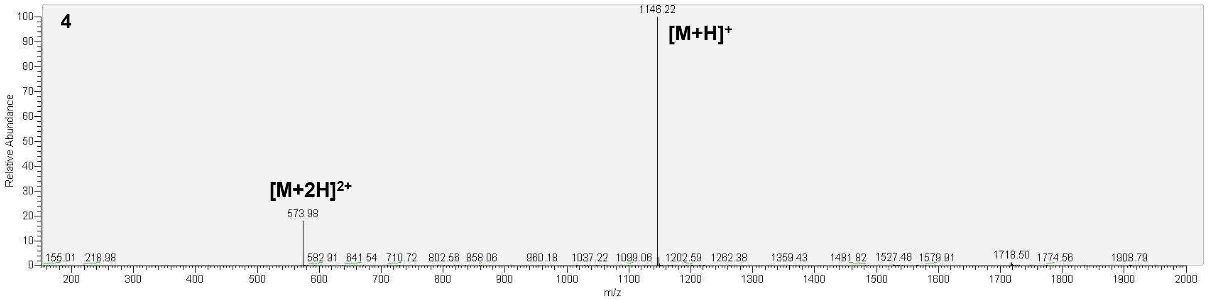

Supporting Information Figure S14. LCMS of purified peptide, **4**, mass calculated for [$C_{54}H_{76}N_{14}O_{14}$ + H] 1145.29; deconvoluted mass observed 1145.57 ± 0.50. Charge states; 573.98 $[M+2H]^{2+}$, 1146.22 $[M+H]^{+}$.

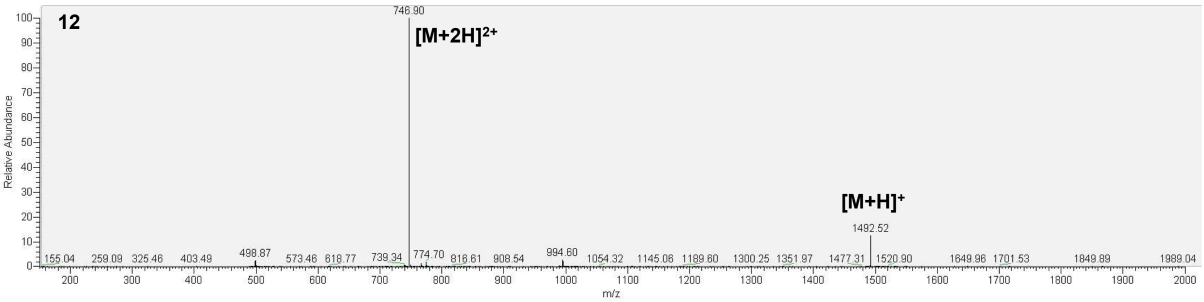

Supporting Information Figure S15. LCMS of purified peptide, **12**, mass calculated for [$C_{73}H_{86}N_{16}O_{17}S$ + H] 1491.65; deconvoluted mass observed: 1491.66 ± 0.20. Charge states; 746.90 $[M+2H]^{2+}$, 1492.52 $[M+H]^{+}$.

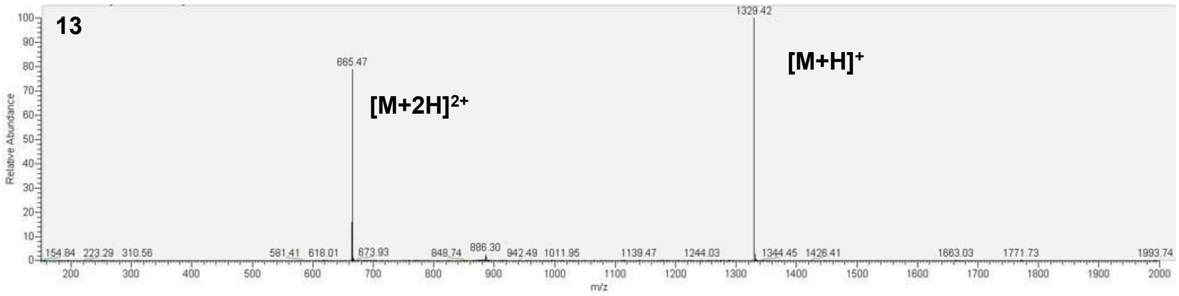

Supporting Information Figure S16. LCMS of purified peptide, **18**, mass calculated for [$C_{62}H_{89}N_{17}O_{14}S$ + H] 1328.56 deconvoluted mass observed: 1328.68 ± 0.37. Charge states; 665.46 $[M+2H]^{2+}$, 1329.42 $[M+H]^{+}$.

[1]H Nuclear Magnetic Resonance (^{1}H NMR)

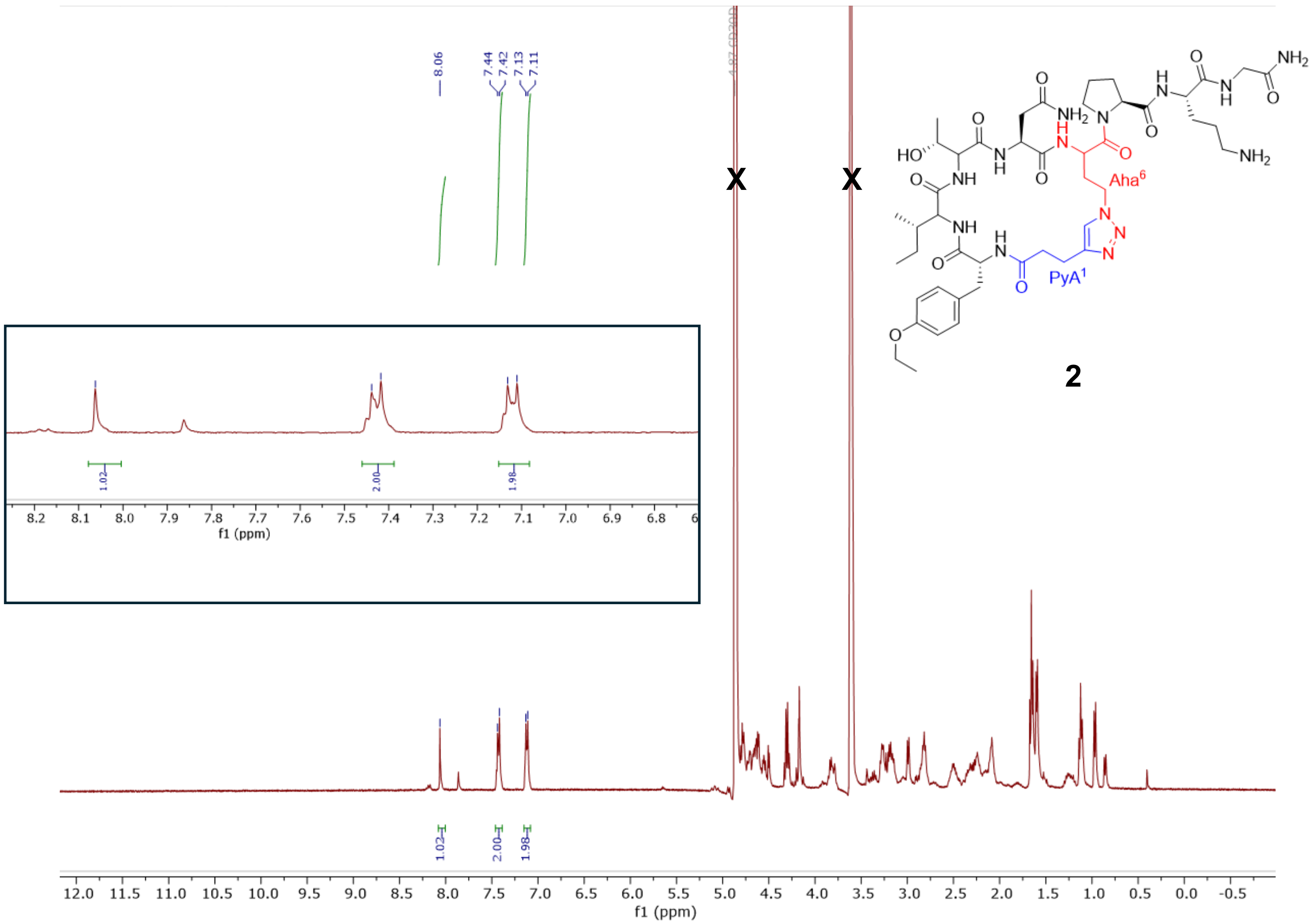

Supporting Information Figure S17. ^{1}H NMR (400 MHz, 298 K, CD_3OD) spectra of the 1,4-triazole mimetic of atosiban (**2**) with amide region of interest inset. Spectrum referenced to CD_3OD 4.78 ppm. X's denotes solvent peaks for CD_3OD and H_2O.

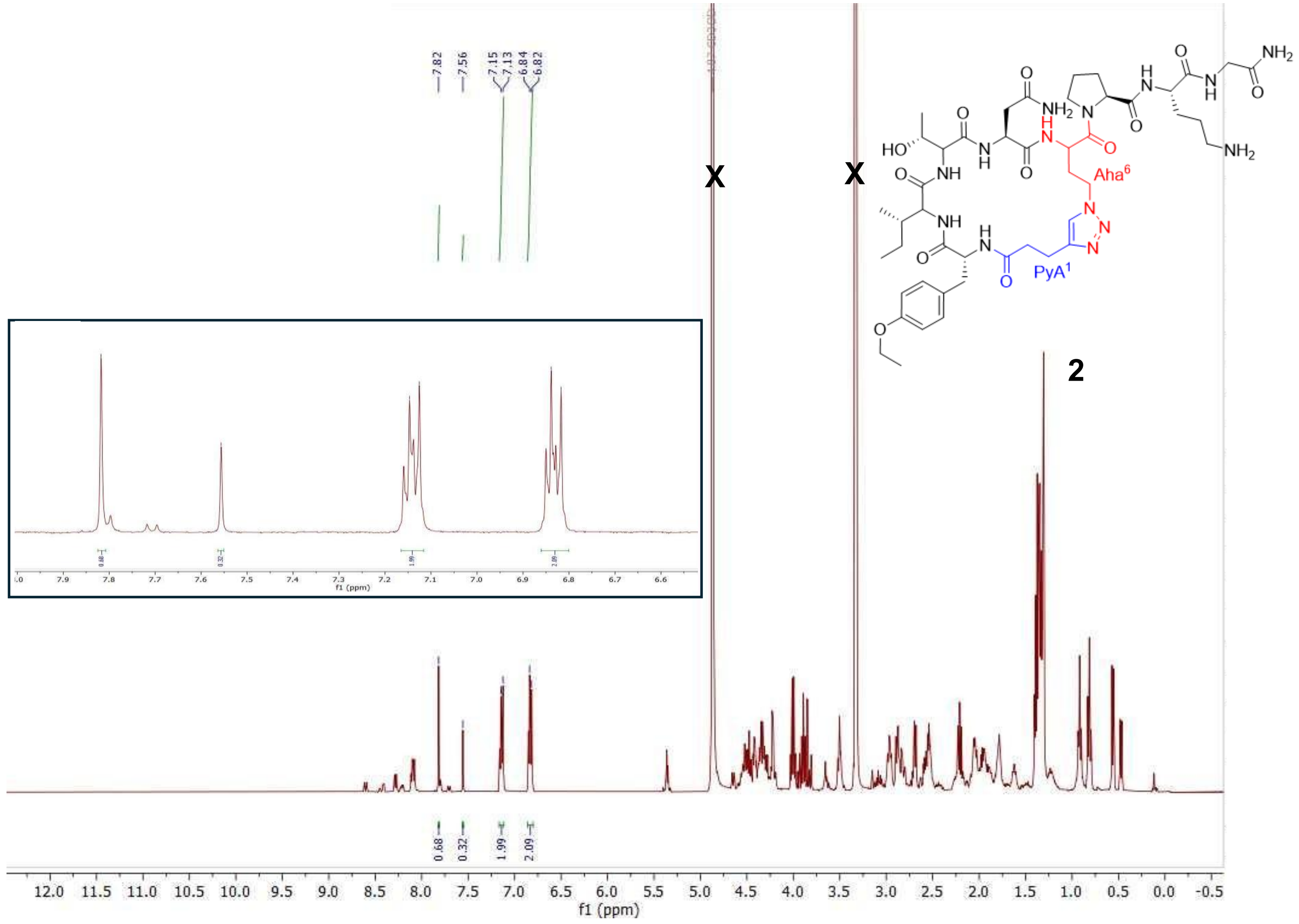

Supporting Information Figure S18. ^{1}H NMR (400 MHz, 323 K, CD_3OD) spectra of the 1,4-triazole mimetic of atosiban (**2**) with amide region of interest inset. Spectrum referenced to CD_3OD 4.78 ppm. X's denotes solvent peaks for CD_3OD and H_2O.

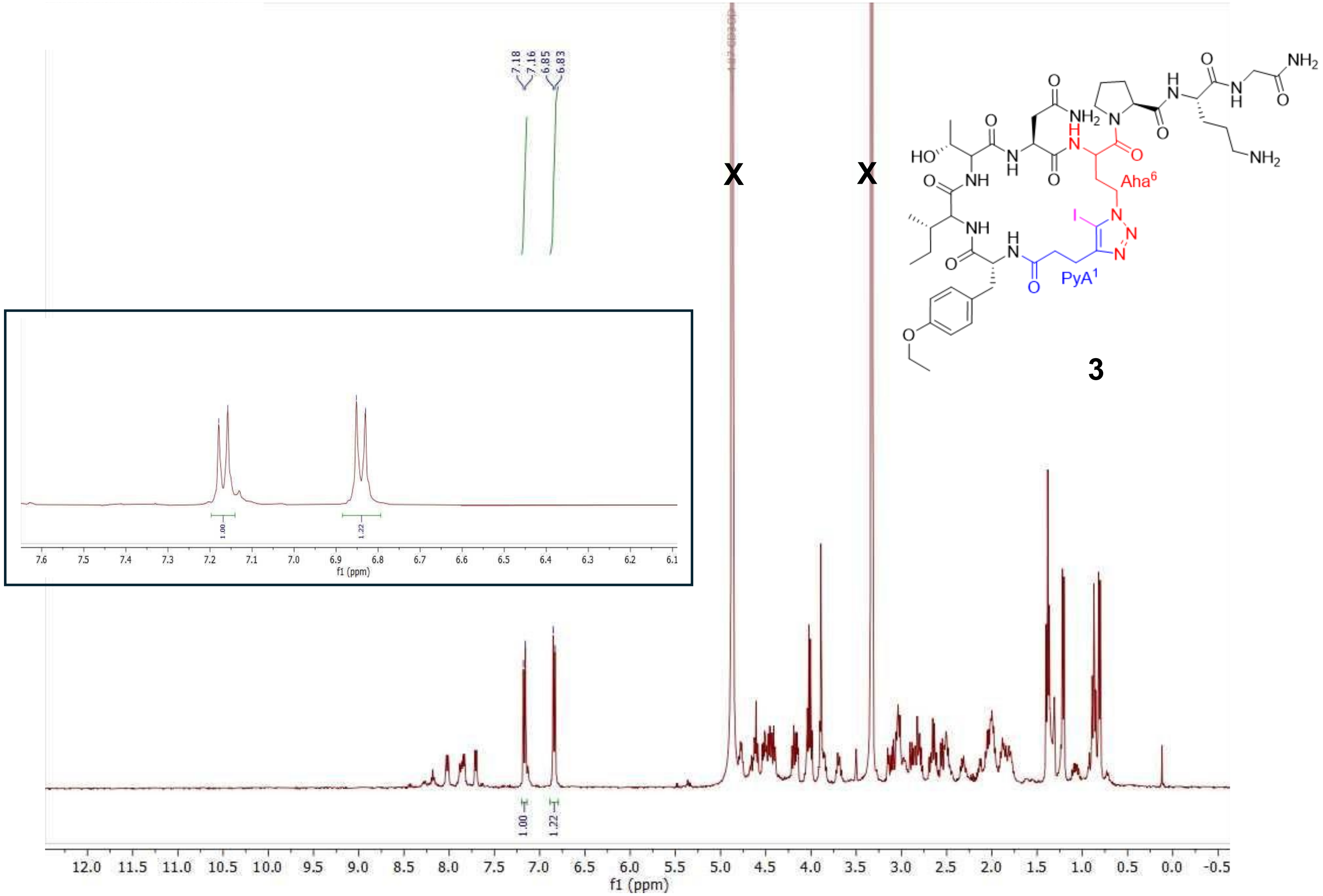

Supporting Information Figure S19. ^{1}H NMR (400 MHz, 298 K, CD_3OD) spectra of the 5-iodo-1,4-triazole mimetic of atosiban (**3**) with amide region of interest inset. Spectrum referenced to CD_3OD 4.78 ppm. X's denotes solvent peaks for CD_3OD and H_2O.

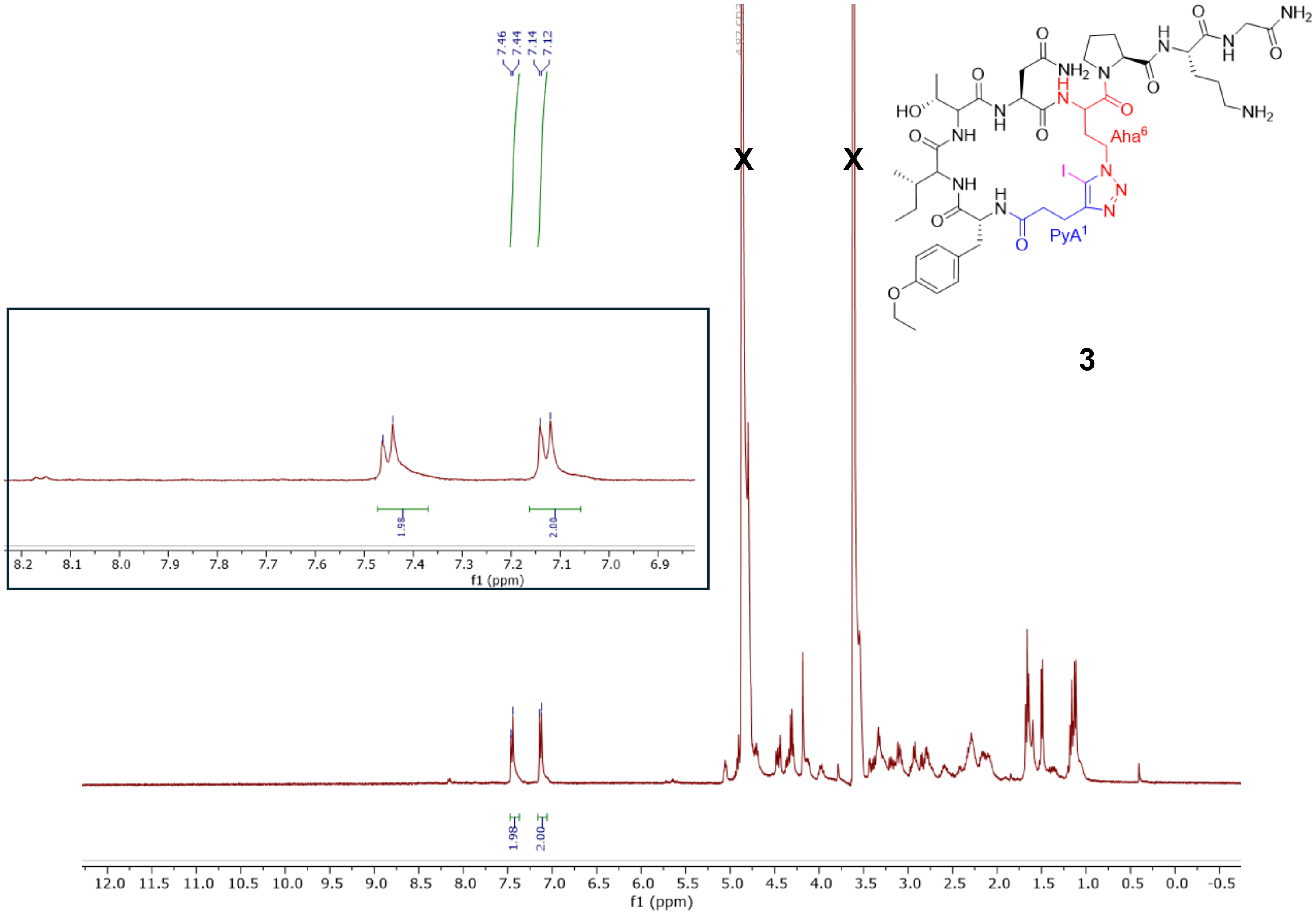

Supporting Information Figure S20. ^{1}H NMR (400 MHz, 323 K, CD_3OD) spectra of the 5-iodo-1,4-triazole mimetic of atosiban (**3**) with region of interest inset. Spectrum referenced to CD_3OD at 4.78 ppm. X's denotes solvent peaks for CD_3OD and H_2O.

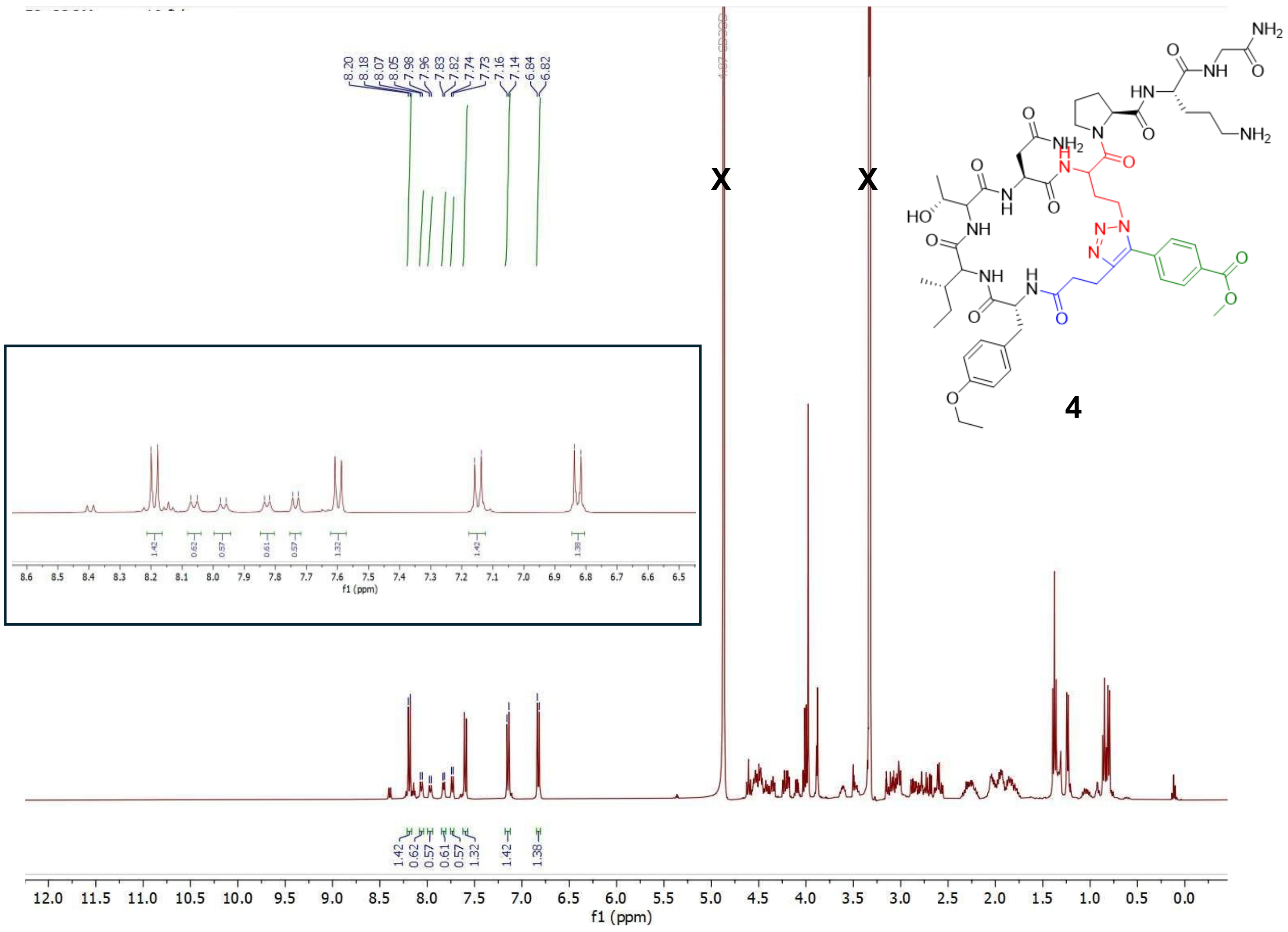

Supporting Information Figure S21. ^{1}H NMR (400 MHz, 298 K, CD_3OD) spectra of the benzyl methyl ester Suzuki functionalized peptide (**4**) with amide region of interest inset. Spectrum referenced to CD_3OD 4.78 ppm. X's denotes solvent peaks for CD_3OD and H_2O.

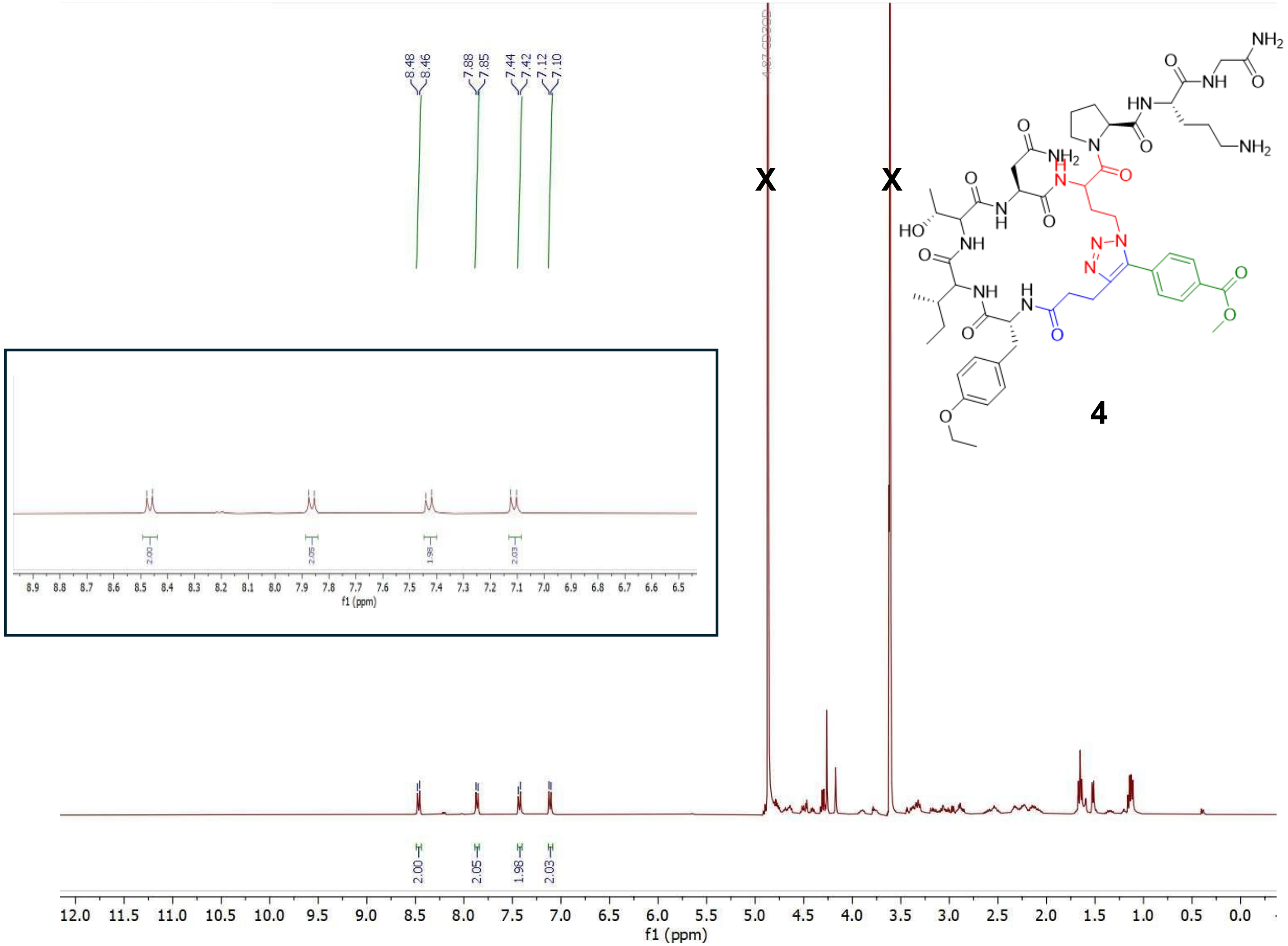

Supporting Information Figure S22. ^{1}H NMR (400 MHz, 323 K, CD_3OD) spectra of the benzyl methyl ester Suzuki functionalized peptide (**4**) with amide region of interest inset. Spectrum referenced to CD_3OD 4.78 ppm. X's denotes solvent peaks for CD_3OD and H_2O.

Infrared (IR) Spectroscopy

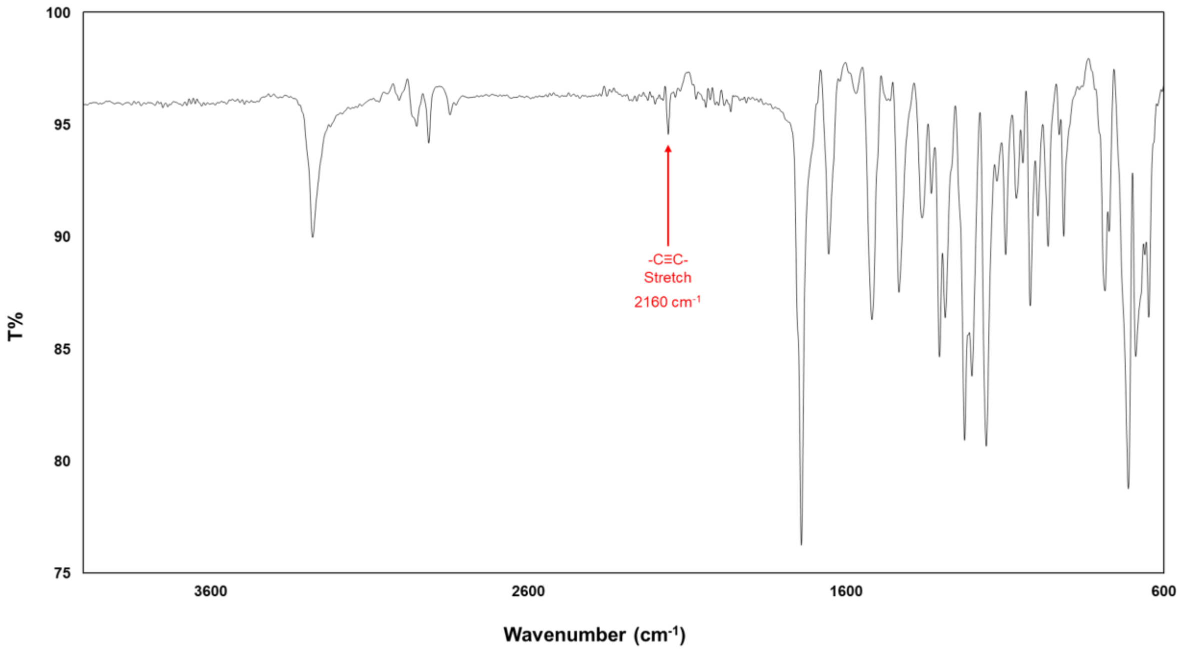

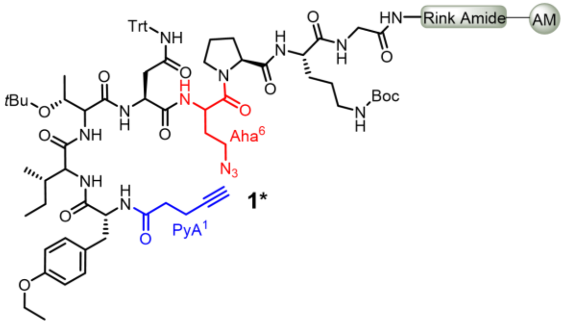

1*

Supporting Information Figure S23. IR of on-resin linear peptide **1***.

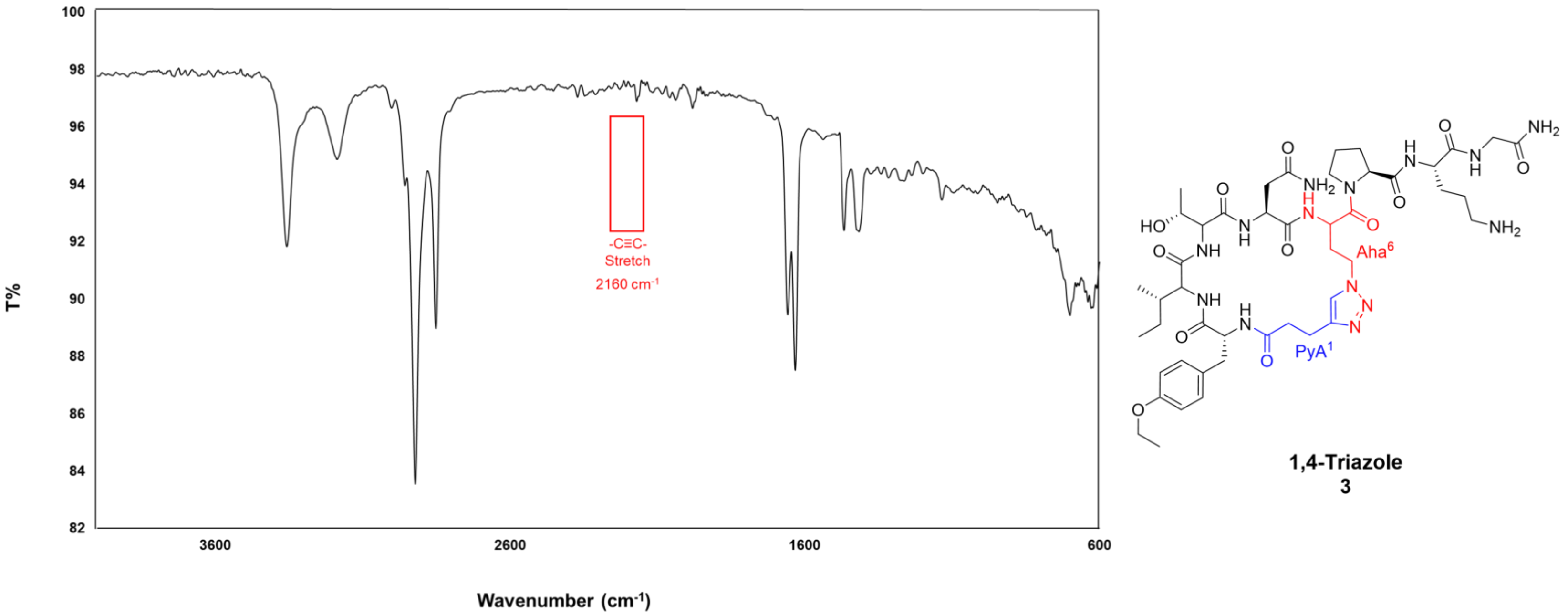

1,4-Triazole
3

Supporting Information Figure S24. IR of purified compound **2**.

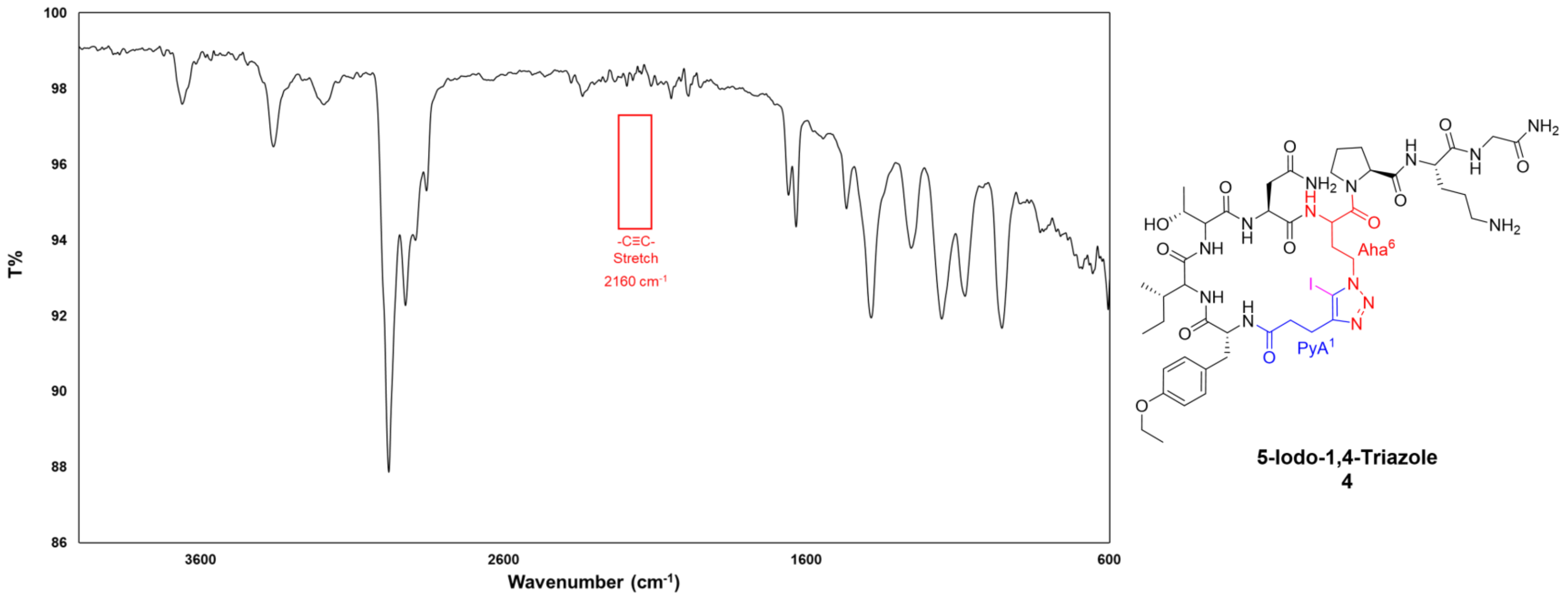

Supporting Information Figure S25. IR of purified compound **3**.

Appendix

Supporting Information Table S2 of 5-Iodo-1,4-Triazole Forming Reactions

RP-HPLC traces for each respective table entry and those masses employed to identify the products of **Supporting Information Table S2** can be found in **Supporting Information Figures S26 – S36**.

Supporting Information Table S2. Attempted reaction conditions and conversion rates for on-resin formation of 5-iodo-1,4-triazole.

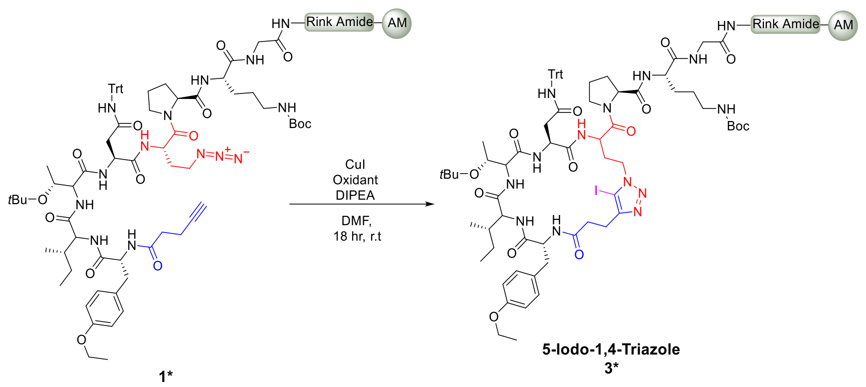

	Reaction Conditions[a]				% area[b]			
Entry	CuI Equiv.	Oxidant	Oxidant Equiv.	DIPEA Equiv.	**1**	Dimer [2M+3H]$^{3+}$	**2**	**3**
1[c]	1.1	*N*-bromosuccinimide	1.2	2	22	5	20	53
2[c]	1.1	*N*-bromosuccinimide	1.2	2	57	2	7	34
3[c]	1.1	*N*-chlorosuccinimide	1.2	2	75	1	24	0
4	1.1	*N*-iodosuccinimide	1.2	2	11	5	11	73
5	1.1	*N*-bromosuccinimide	2	2	52	6	40	2
6	2.2	*N*-bromosuccinimide	1.2	2	1	3	11	85
7[d]	2.2	*N*-bromosuccinimide	1.2	2	3	2	11	82

[a] The peptidyl resin (0.01 mmol, TentaGel-S®-Rink-Amide: 0.23 mmol/g) was swollen in DMF (10 mL final volume per 0.1 mmol resin) with DIPEA for 5 mins prior to the addition of any further reagents. CuI in DMF (400 µL) and oxidant in DMF (400 µL) were solubilized separately and combined immediately prior to addition to the peptidyl resin. The reaction was left, overnight at r.t. Upon reaction completion, the peptidyl resin was washed with DMF (3 x 5 mL), 20% piperidine + 5% formic acid in DMF (2 x 5 mL, *v/v/v*), DMF (3 x 5 mL), CH_2Cl_2 (3 x 5 mL), and dried under vacuum.[2] Reaction progression was monitored by cleavage of a small portion of the peptidyl resin and analyzed by RP-HPLC and LCMS. [b] % area determined at 214 nm and rounded to the nearest integer. Peak ratios reported for starting material (**1**), dimeric iodo-triazole products, dehalogenated triazole (**2**), and 5-iodo-1,4-triazole (**3**). [c] These reactions were carried out in the absence of light. [d] denotes large scale (0.3 mmol) reaction. * denotes the on-resin peptide.

HPLC Traces

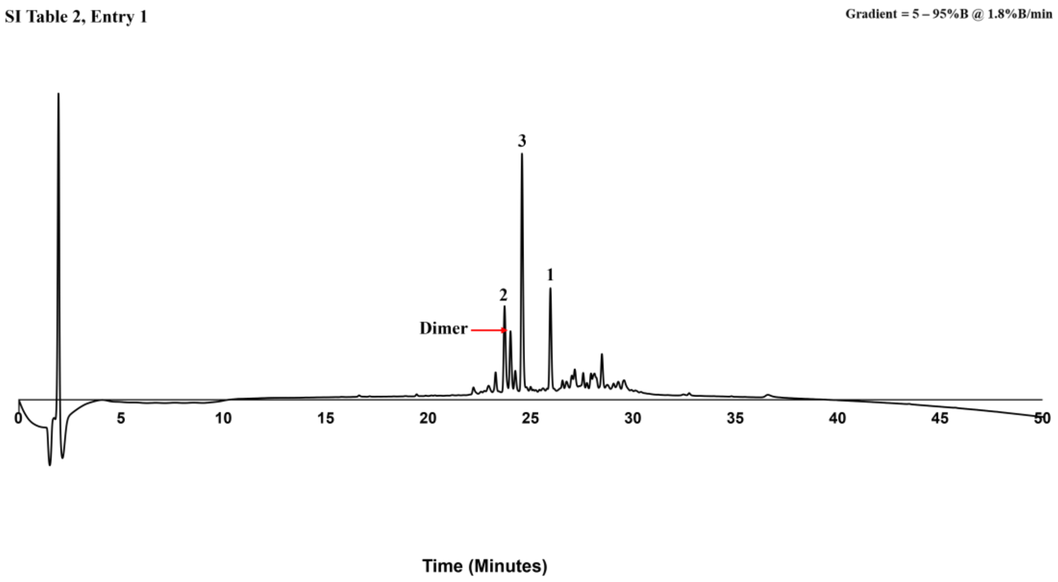

Supporting Information Figure S26. Analytical RP-HPLC chromatogram (214 nm) of crude peptide liberated from the peptidyl resin following treatment with conditions outlined in **Supporting Information Table S2, Entry 1**. Aeris Peptide XB-C18 (100 Å, 5 µm, 150 mm x 4.6 mm), linear gradient 5% – 95%B over 50 min (*ca.* 1.8%B/min) at 1 mL/min.

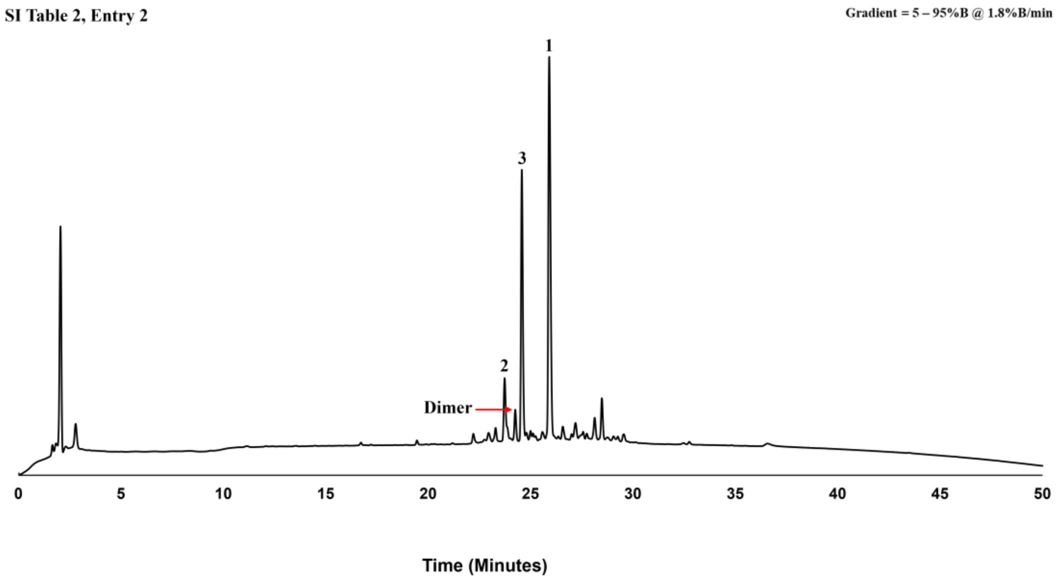

Supporting Information Figure S27. Analytical RP-HPLC chromatogram (214 nm) of crude peptide liberated from the peptidyl resin following treatment with conditions outlined in **Supporting Information Table S2, Entry 2**. Aeris Peptide XB-C18 (100 Å, 5 µm, 150 mm x 4.6 mm), linear gradient 5% – 95%B over 50 min (*ca.* 1.8%B/min) at 1 mL/min.

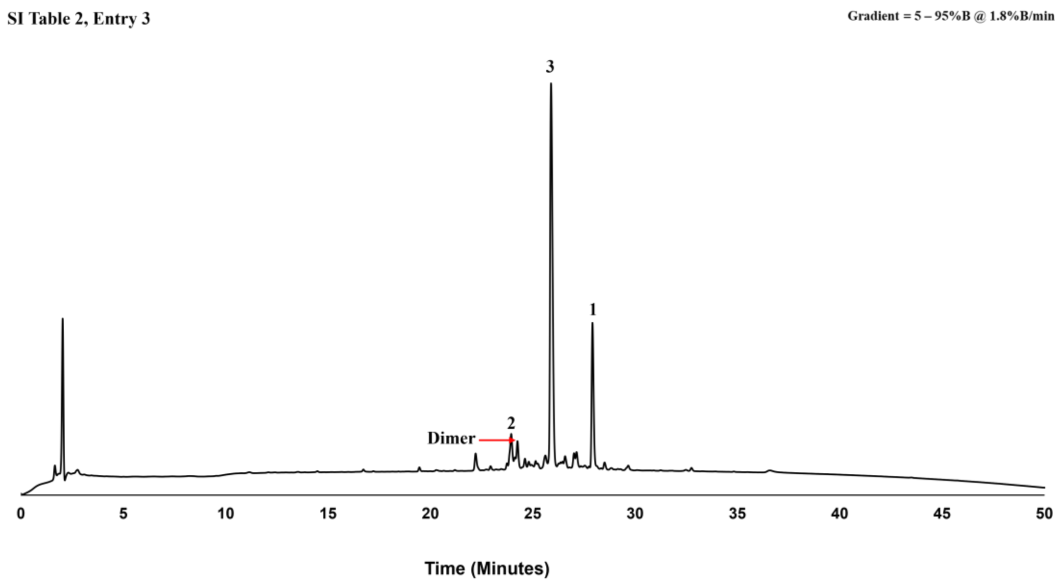

Supporting Information Figure S28. Analytical RP-HPLC chromatogram (214 nm) of crude peptide liberated from the peptidyl resin following treatment with conditions outlined in **Supporting Information Table S2, Entry 3**. Aeris Peptide XB-C18 (100 Å, 5 µm, 150 mm x 4.6 mm), linear gradient 5% – 95%B over 50 min (*ca.* 1.8%B/min) at 1 mL/min.

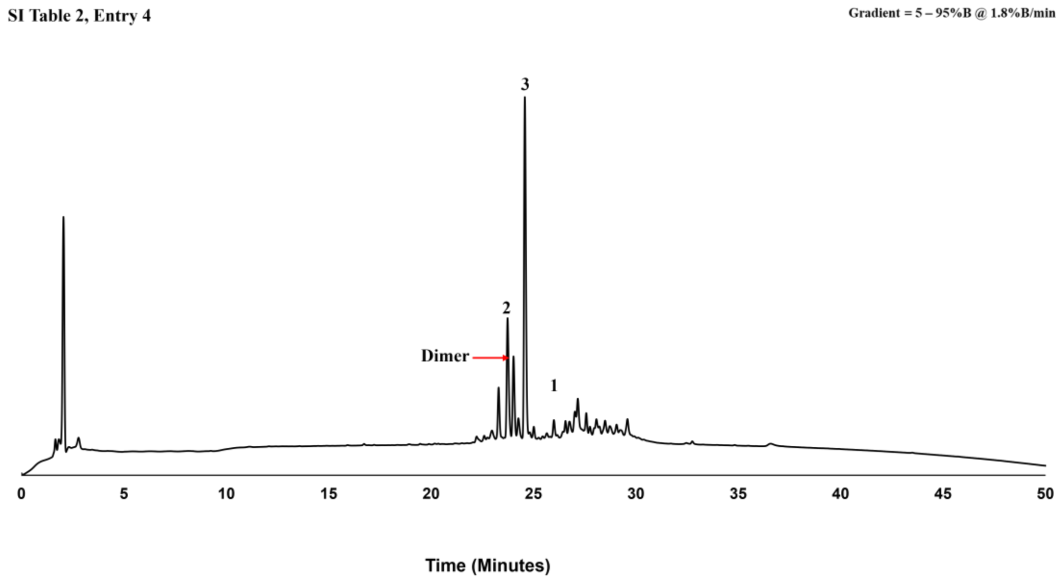

Supporting Information Figure S29. Analytical RP-HPLC chromatogram (214 nm) of crude peptide liberated from the peptidyl resin following treatment with conditions outlined in **Supporting Information Table S2, Entry 4**. Aeris Peptide XB-C18 (100 Å, 5 µm, 150 mm x 4.6 mm), linear gradient 5% – 95%B over 50 min (*ca.* 1.8%B/min) at 1 mL/min.

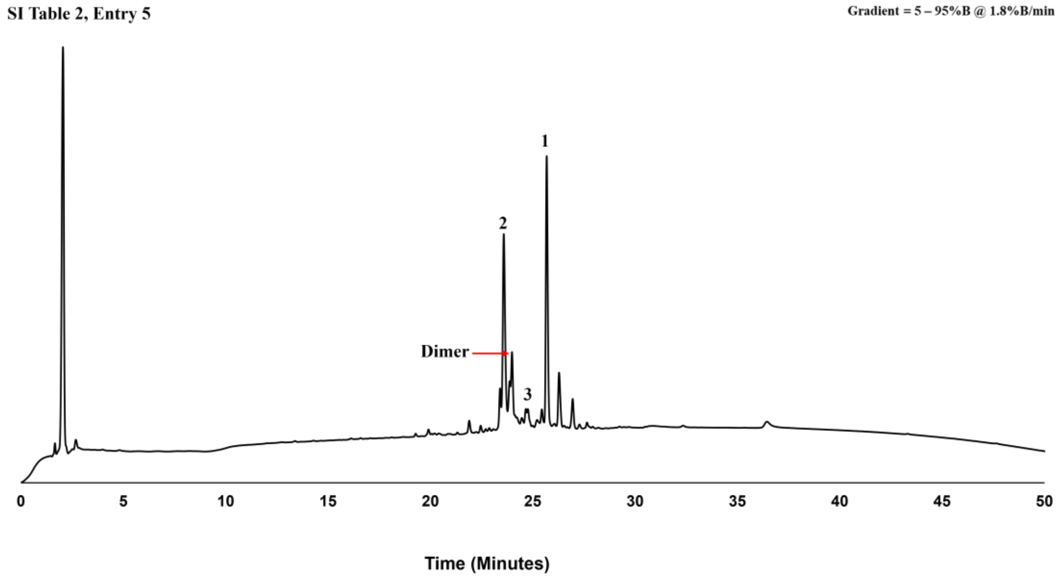

Supporting Information Figure S30. Analytical RP-HPLC chromatogram (214 nm) of crude peptide liberated from the peptidyl resin following treatment with conditions outlined in **Supporting Information Table S2, Entry 5**. Aeris Peptide XB-C18 (100 Å, 5 µm, 150 mm x 4.6 mm), linear gradient 5% – 95%B over 50 min (*ca.* 1.8%B/min) at 1 mL/min.

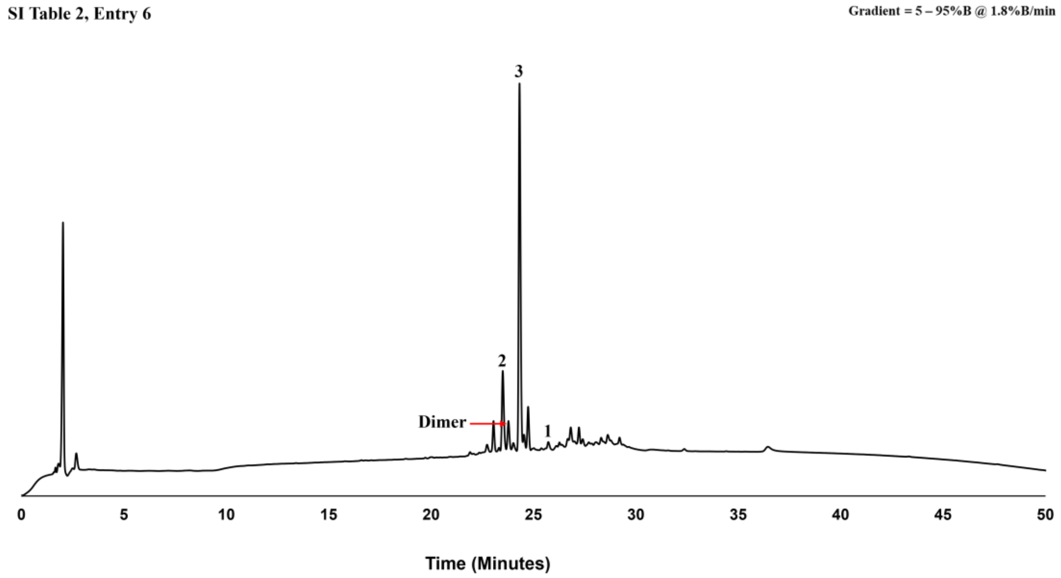

Supporting Information Figure S31. Analytical RP-HPLC chromatogram (214 nm) of crude peptide liberated from the peptidyl resin following treatment with conditions outlined in **Supporting Information Table S2, Entry 6**. Aeris Peptide XB-C18 (100 Å, 5 µm, 150 mm x 4.6 mm), linear gradient 5% – 95%B over 50 min (*ca.* 1.8%B/min) at 1 mL/min.

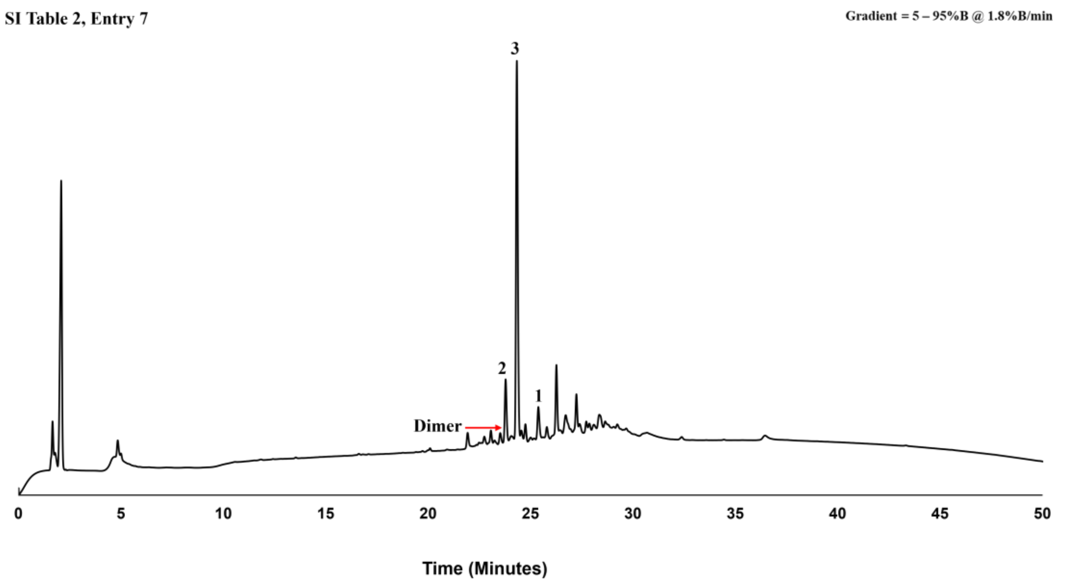

Supporting Information Figure S32. Analytical RP-HPLC chromatogram (214 nm) of crude peptide liberated from the peptidyl resin following treatment with conditions outlined in **Supporting Information Table S2, Entry 7**. Aeris Peptide XB-C18 (100 Å, 5 µm, 150 mm x 4.6 mm), linear gradient 5% – 95%B over 50 min (*ca.* 1.8%B/min) at 1 mL/min.

LCMS

Peaks identified from LCMS of peptidyl peaks found in analytical RP-HPLC traces of **Supporting Information Table S2, Entry 8**. Peak masses were consistent across all **Supporting Information Table S2** entries at the respective retention times.

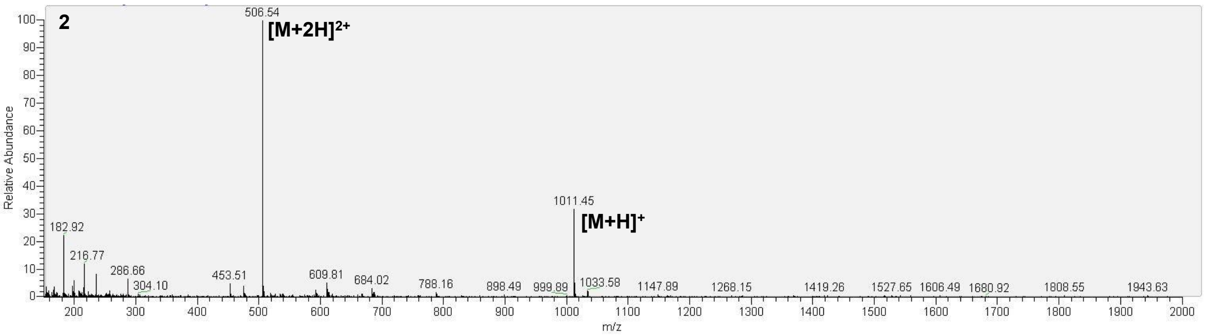

Supporting Information Figure S33. LCMS of peptidyl product at t_R = 23.6 min, following resin cleavage and global deprotection of peptidyl resin **3***, corresponding to undesired peptide product **2**. Mass calculated for [$C_{46}H_{70}N_{14}O_{12}$ + H] 1011.15; deconvoluted mass observed: 1010.76 ± 0.37. Charge states; 506.51 $[M+2H]^{2+}$, 1011.49 $[M+H]^{+}$.

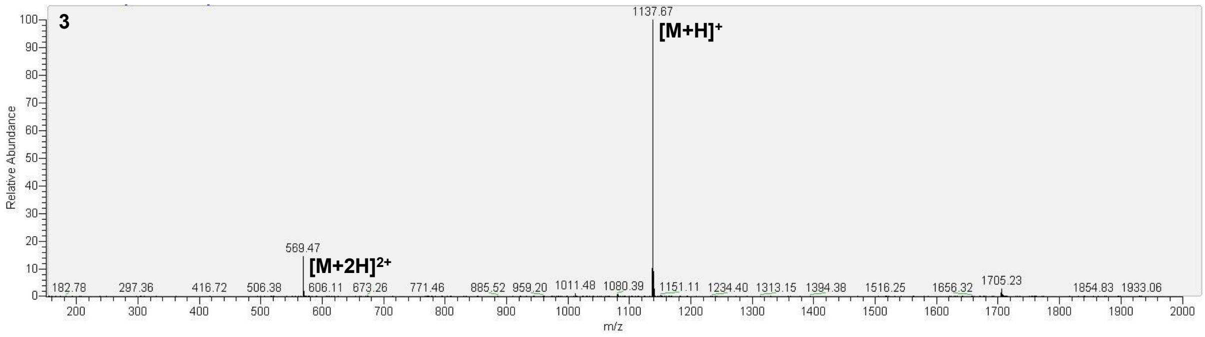

Supporting Information Figure S34. LCMS of peptidyl product at t_R = 24.3 min, following resin cleavage and global deprotection of peptidyl resin **3***, corresponding to desired peptide product **3**. Mass calculated for [$C_{46}H_{69}IN_{14}O_{12}$ + H] 1137.05; deconvoluted mass observed: 1137.01 ± 0.41. Charge states; 569.65 $[M+2H]^{2+}$, 1137.72 $[M+H]^{+}$.

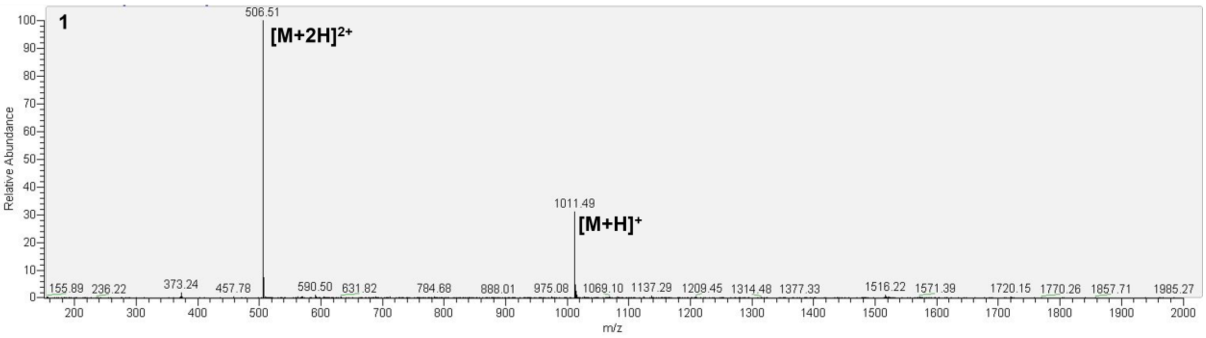

Supporting Information Figure S35. LCMS of peptidyl product at t_R = 25.6 min, following resin cleavage and global deprotection of peptidyl resin **3***, corresponding to undesired peptide product **1**. Mass calculated for [$C_{46}H_{70}N_{14}O_{12}$ + H] 1011.15; deconvoluted mass observed: 1011.07 ± 0.39. Charge states; 506.67 $[M+2H]^{2+}$, 1011.79 $[M+H]^{+}$.

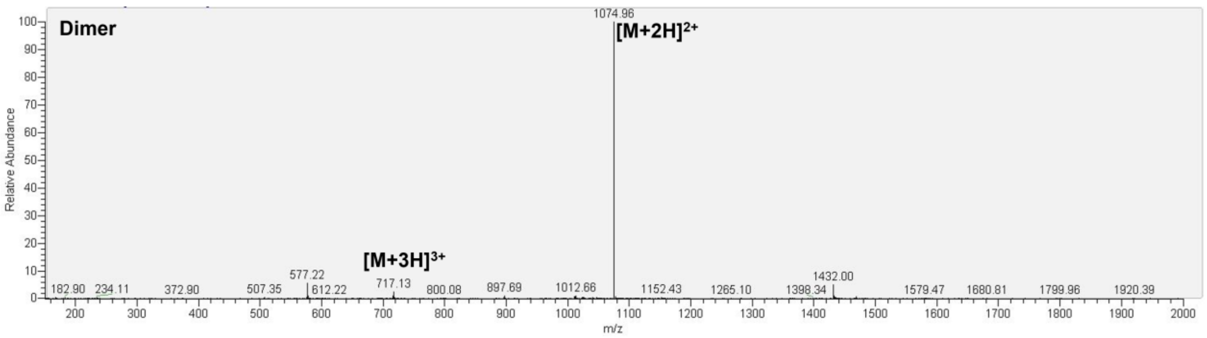

Supporting Information Figure S36. LCMS of peptidyl product at t_R = 23.9 min, following resin cleavage and global deprotection of peptidyl resin 3*, corresponding to undesired peptide product of covalent dimer. Mass calculated for $[C_{92}H_{139}IN_{28}O_{24} + H]$ 2148.20; deconvoluted mass observed: 2148.16 ± 0.33. Charge states; 717.13 $[M+3H]^{3+}$, 1074.96 $[M+2H]^{2+}$.

Supporting Information Tables S3 and S4 of Suzuki-Miyaura Functionalization of 5-Iodo-1,4-Triazole

RP-HPLC traces for each respective table entry and those masses employed to identify the products of **Supporting Information Tables S3 and S4** can be found in **Supporting Information Figures S37 – S66**.

Supporting Information Table S3. Further attempted reaction conditions and conversion rates for on-resin Suzuki-Miyaura coupling to 5-iodo-1,4-triazole.

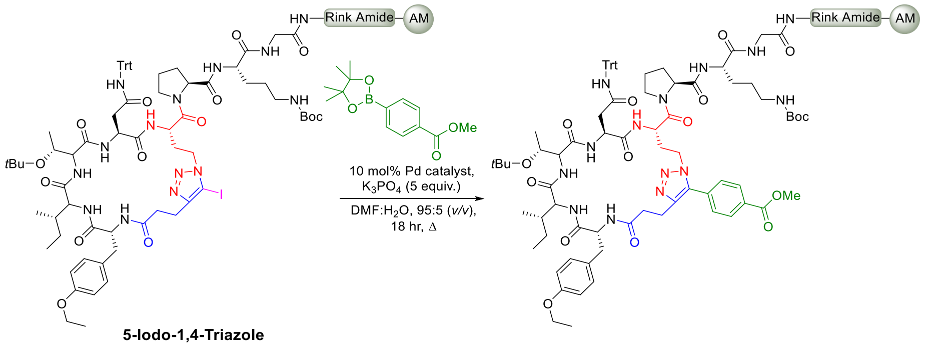

5-Iodo-1,4-Triazole
3*

4*

	Reaction Conditions[a]					% Area[b]			
Entry	Catalyst (10 mol%)	K_3PO_4 equiv.	Solvent	Boronic Ester Equiv.	Temp. °C	SM (**3**)	DH (**2**)	P (**4**)	O
1	Pd(II)(RuPhos)	5	DMF	5	80	21	40	0	39
2	Pd(II)(*t*BuPhos)	5	DMF	5	80	30	33	0	37
3	Pd(II)(SPhos)	5	DMF	5	80	16	44	0	40
4	Pd(II)(XPhos)	5	DMF	5	80	25	42	0	33
5	$Pd(0)(PPh_3)_4$	5	DMF	5	80	0	54	11	35
6	$Pd(II)(OAc)_2$	5	DMF	5	80	38	31	0	31
7	$Pd(II)(PPh_3)_2Cl_2$	5	DMF	5	80	29	42	0	29
8	$Pd(II)(dppf)Cl_2$	5	DMF	5	80	29	40	0	31
9	$Pd(II)(dppf)Cl_2·CH_2Cl_2$	5	DMF	5	80	30	40	0	30
10	$Pd(0)(PPh_3)_4$	5	DMF	5	60	3	60	37	0
11	$Pd(0)(PPh_3)_4$	5	DMF	5	40	24	45	7	24
12	$Pd(0)(PPh_3)_4$	5	DMF	5	25	44	25	6	25
13	$Pd(0)(PPh_3)_4$	10	DMF	5	60	20	72	8	0
14[c]	$Pd(0)(PPh_3)_4$	5	DMF	5	60	41	48	11	0
15	$Pd(0)(PPh_3)_4$	5	DMF	10	60	4	65	31	0
16	$Pd(0)(PPh_3)_4$	10	DMF	10	60	19	69	12	0
17	$Pd(0)(PPh_3)_4$	5	DMF	25	60	0	25	75	0

18	$Pd(0)(PPh_3)_4$	5	DMF	50	60	2	31	67	0
19	$Pd(0)(PPh_3)_4$	5	DMF	100	60	2	17	81	0
20	$Pd(0)(PPh_3)_4$	5	DMF	150	60	0	15	85	0
21	$Pd(0)(PPh_3)_4$	5	Dioxane	50	60	4	24	72	0
22	$Pd(0)(PPh_3)_4$	5	Dioxane	10	60	10	23	67	0

[a] The peptidyl resin (0.01 mmol, TentaGel-S®-Rink-Amide: 0.23 mmol/g) was swollen in the respective solvent (5 mL per 0.1 mmol resin) for 5 minutes prior to the addition of any further reagents. Following resin swelling, K_3PO_4 (5 equiv.) in H_2O (5% *v/v* of total final volume (350 µL) and 4-methoxycarbonylphenylboronic acid pinacol ester in the respective solvent (1.15 mL) were added to the solution. The resin containing solution was then degassed with argon (10 mins) prior to reaction initiation by addition of the respective Pd cat. (10 mol%) in the respective solvent (500 µL). Upon reaction completion, the peptidyl resin was washed with DMF (3 x 5 mL), 20% piperidine + 5% formic acid in DMF (2 x 5 mL, *v/v/v*), DMF (3 x 5 mL), CH_2Cl_2 (3 x 5 mL), and dried under vacuum. Reaction progression was monitored by cleavage of a small portion of the peptidyl resin and analyzed by RP-HPLC and LCMS. [b] % area determined at 214 nm and rounded to the nearest integer. Peak ratios reported for starting material (SM, **3**), dehalogenated triazole (DH, **2**), product (P, **4**) and, other unidentifiable product >5% total peak area (O). [c] H_2O volume used was 2.5% final total volume (175 µL, *v/v*). * denotes the on-resin peptide.

Supporting Information Table S4. Final reaction conditions and conversion rates for on-resin Suzuki-Miyaura coupling to 5-iodo-1,4-triazole.

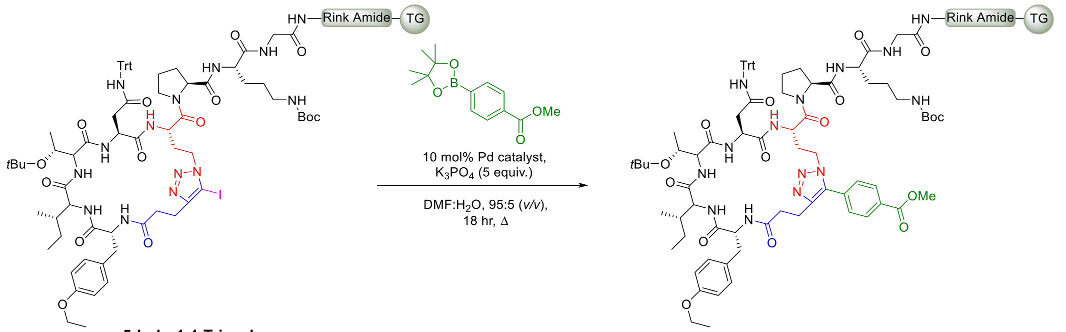

5-Iodo-1,4-Triazole
3*

4*

Reaction Conditions[a]				% Area[b]			
Entry	Resin	Solvent	Volume	SM (**3**)	DH (**2**)	P (**4**)	O
1	ChemMatrix	DMF	7 mL	12	48	40	0
2	ChemMatrix	Dioxane	7 mL	7	23	70	0
3	ChemMatrix	DMF	2 mL	0	41	52	7
4	ChemMatrix	Dioxane	2 mL	0	24	76	0
5	AminoMethyl	Dioxane	2 mL	6	21	73	0

[a] The peptidyl resin (0.01 mmol, ChemMatrix®: 0.37 mmol/g, or AminoMethyl polystyrene: 0.53 mmol/g) was swollen in the respective solvent (1 or 5 mL per 0.1 mmol resin) for 5 minutes prior to the addition of any further reagents. Following resin swelling, K_3PO_4 (5 equiv.) in H_2O (5% *v/v* of total final volume (100 or 350 µL) and 4-methoxycarbonylphenylboronic acid pinacol ester (10 equiv.) in respective solvent (400 or 1150 µL) were added to the solution. The resin containing solution was then degassed with argon (10 mins) prior to reaction initiation by addition of the respective Pd cat. (10 mol%) in the respective solvent (500 µL). Upon reaction completion, the peptidyl resin was washed with DMF (3 x 5 mL), 20% piperidine + 5% formic acid in DMF (2 x 5 mL, *v/v/v*), DMF (3 x 5 mL), CH_2Cl_2 (3 x 5 mL), and dried under vacuum. Reaction progression was monitored by cleavage of a small portion of the peptidyl resin and analyzed by RP-HPLC and LCMS. [b] % area determined at 214 nm and rounded to the nearest integer. Peak ratios reported for starting material (SM, **3**), dehalogenated triazole (DH, **2**), product (P, **4**) and, other unidentifiable product >5% total peak area (O). * denotes the on-resin peptide.

HPLC Traces

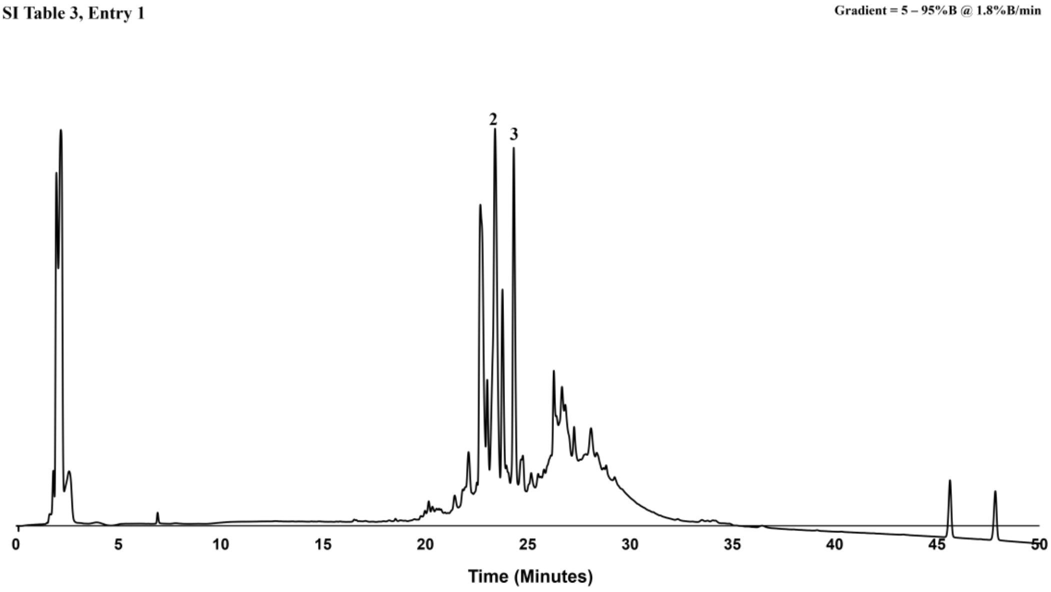

Supporting Information Figure S37. Analytical RP-HPLC chromatogram (214 nm) of crude peptide liberated from the peptidyl resin following treatment with conditions outlined in **Supporting Information Table S3, Entry 1**. Aeris Peptide XB-C18 (100 Å, 5 µm, 150 mm x 4.6 mm), linear gradient 5% – 95%B over 50 min (*ca.* 1.8%B/min) at 1 mL/min.

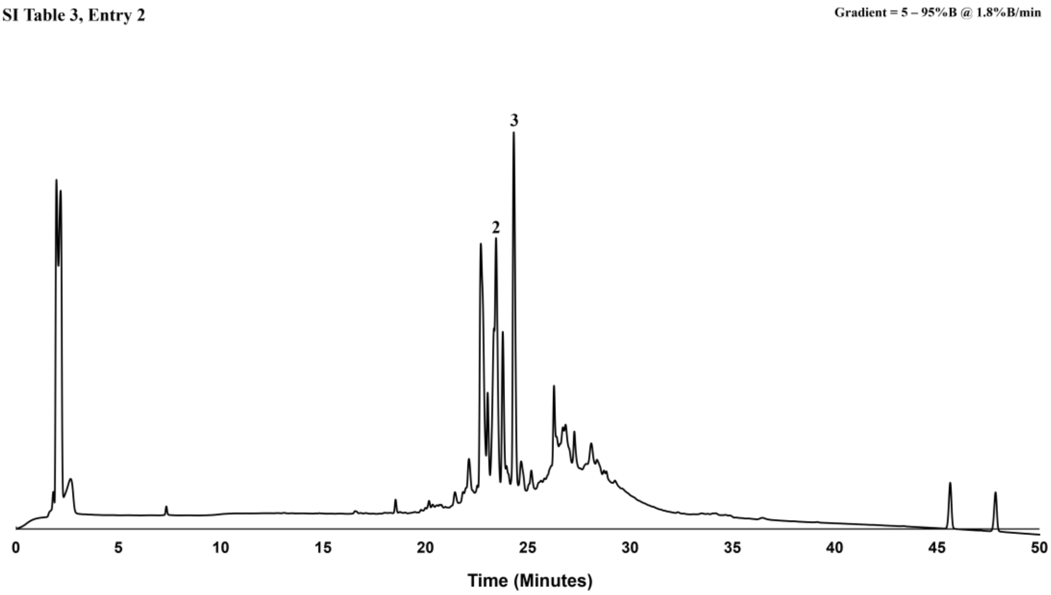

Supporting Information Figure S38. Analytical RP-HPLC chromatogram (214 nm) of crude peptide liberated from the peptidyl resin following treatment with conditions outlined in **Supporting Information Table S3, Entry 2**. Aeris Peptide XB-C18 (100 Å, 5 µm, 150 mm x 4.6 mm), linear gradient 5% – 95%B over 50 min (*ca.* 1.8%B/min) at 1 mL/min.

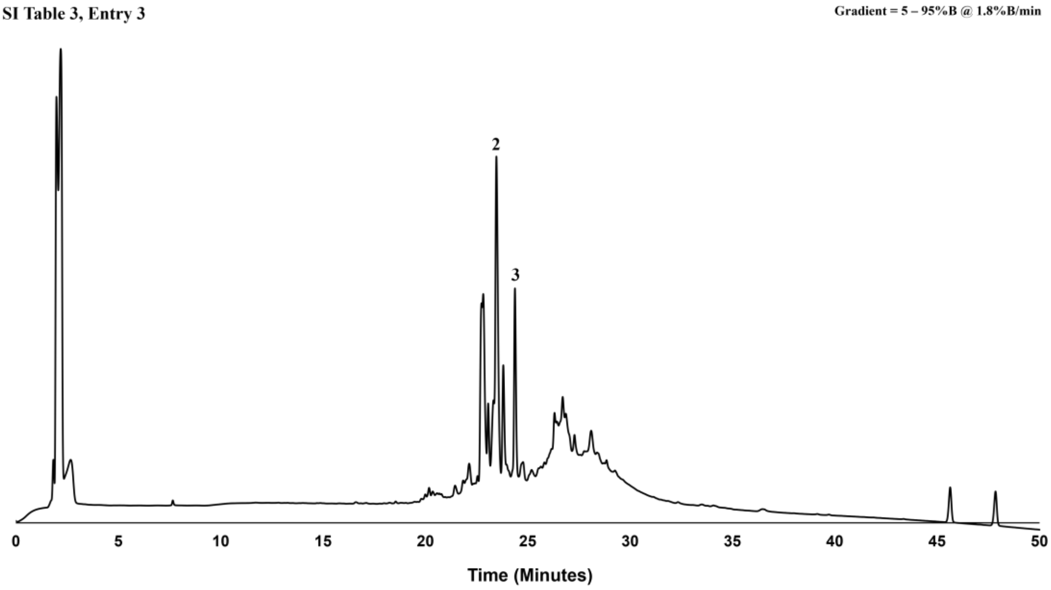

Supporting Information Figure S39. Analytical RP-HPLC chromatogram (214 nm) of crude peptide liberated from the peptidyl resin following treatment with conditions outlined in **Supporting Information Table S3, Entry 3**. Aeris Peptide XB-C18 (100 Å, 5 µm, 150 mm x 4.6 mm), linear gradient 5% – 95%B over 50 min (*ca.* 1.8%B/min) at 1 mL/min.

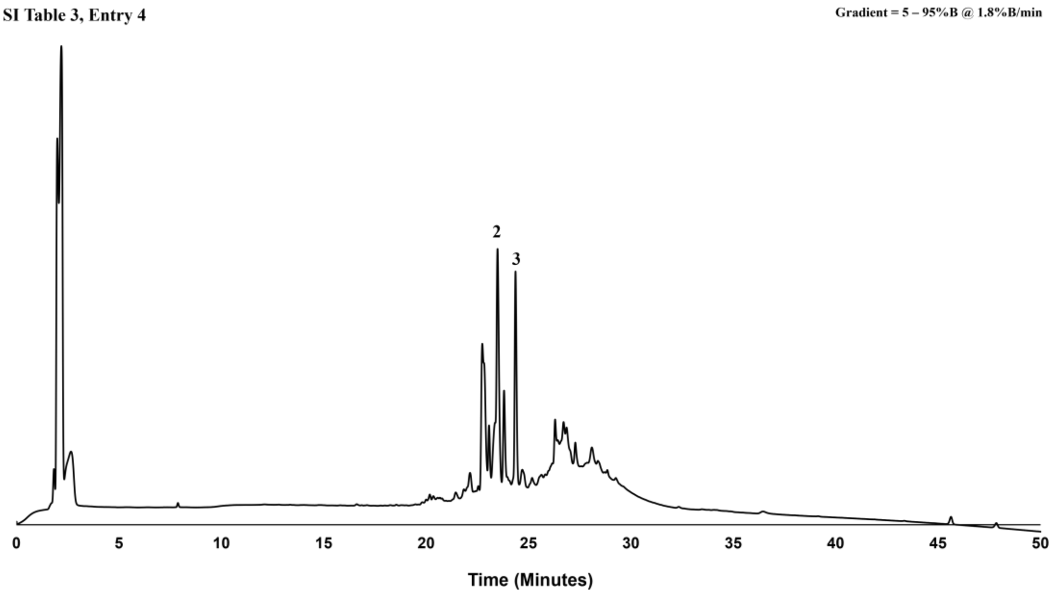

Supporting Information Figure S40. Analytical RP-HPLC chromatogram (214 nm) of crude peptide liberated from the peptidyl resin following treatment with conditions outlined in **Supporting Information Table S3, Entry 4**. Aeris Peptide XB-C18 (100 Å, 5 µm, 150 mm x 4.6 mm), linear gradient 5% – 95%B over 50 min (*ca.* 1.8%B/min) at 1 mL/min.

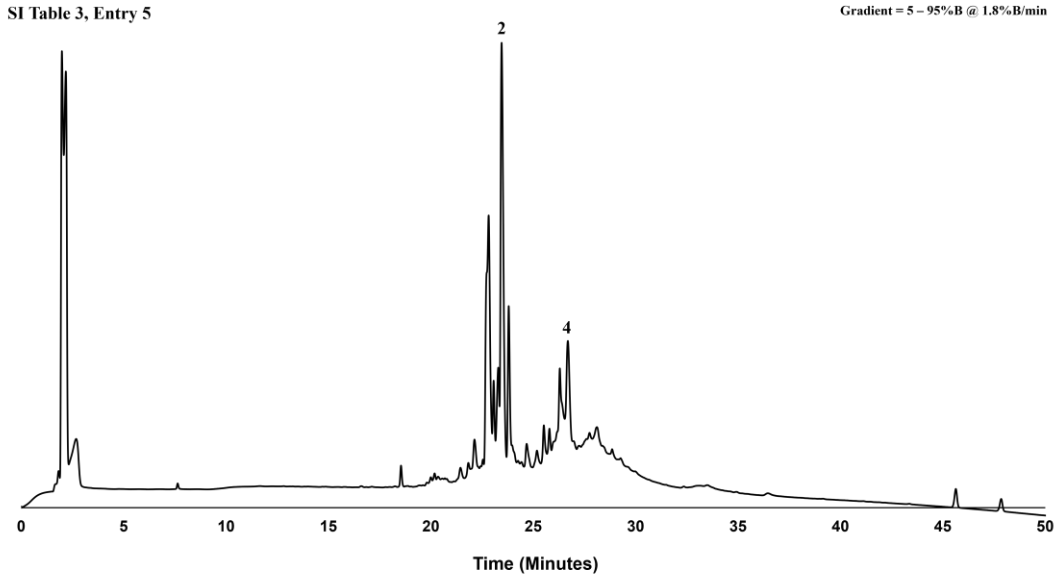

Supporting Information Figure S41. Analytical RP-HPLC chromatogram (214 nm) of crude peptide liberated from the peptidyl resin following treatment with conditions outlined in **Supporting Information Table S3, Entry 5**. Aeris Peptide XB-C18 (100 Å, 5 µm, 150 mm x 4.6 mm), linear gradient 5% – 95%B over 50 min (*ca.* 1.8%B/min) at 1 mL/min.

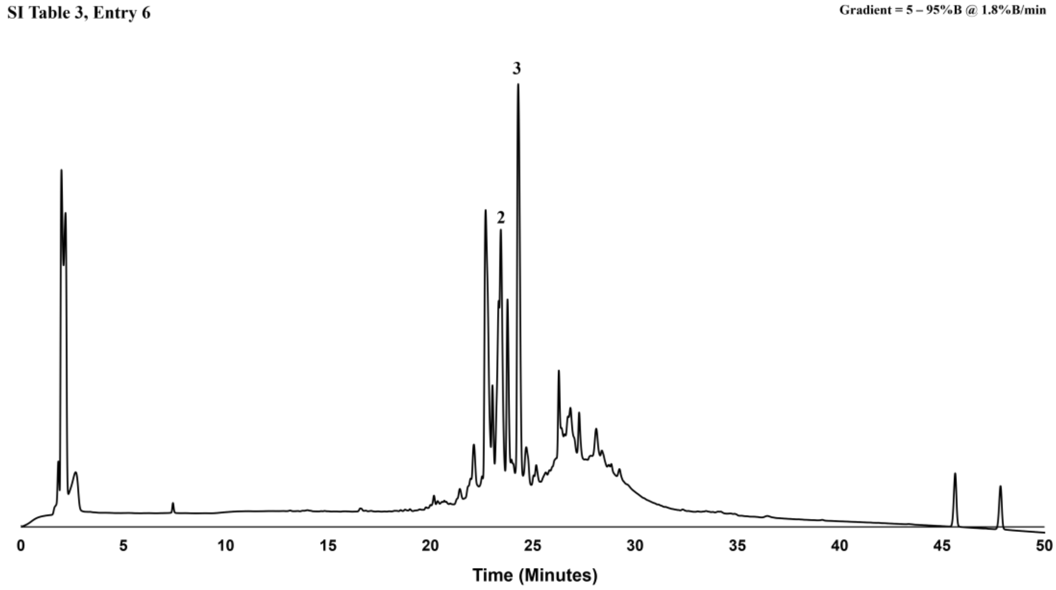

Supporting Information Figure S42. Analytical RP-HPLC chromatogram (214 nm) of crude peptide liberated from the peptidyl resin following treatment with conditions outlined in **Supporting Information Table S3, Entry 6**. Aeris Peptide XB-C18 (100 Å, 5 µm, 150 mm x 4.6 mm), linear gradient 5% – 95%B over 50 min (*ca.* 1.8%B/min) at 1 mL/min.

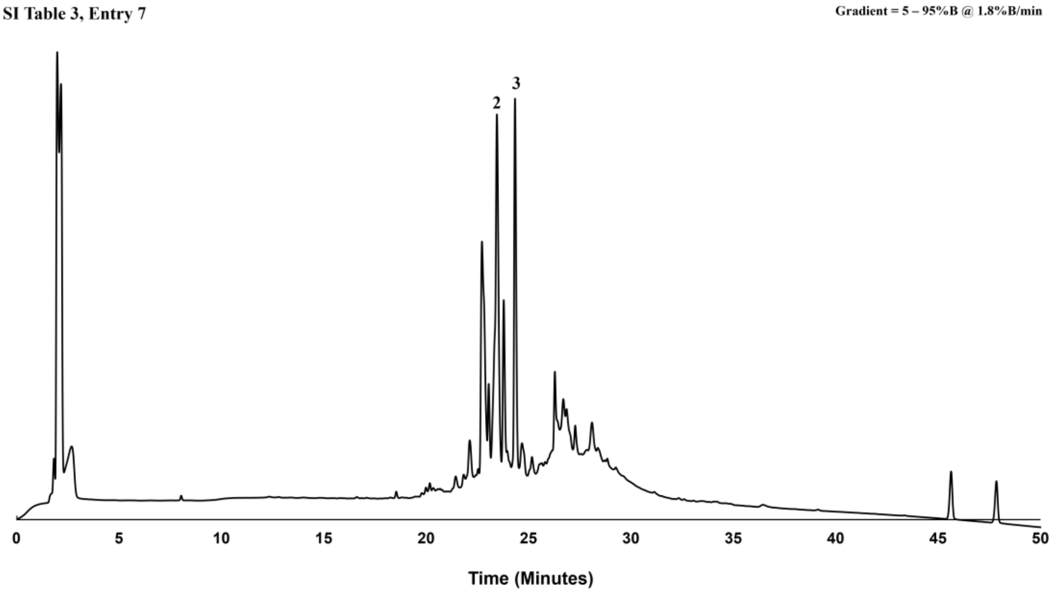

Supporting Information Figure S43. Analytical RP-HPLC chromatogram (214 nm) of crude peptide liberated from the peptidyl resin following treatment with conditions outlined in **Supporting Information Table S3, Entry 7**. Aeris Peptide XB-C18 (100 Å, 5 µm, 150 mm x 4.6 mm), linear gradient 5% – 95%B over 50 min (*ca.* 1.8%B/min) at 1 mL/min.

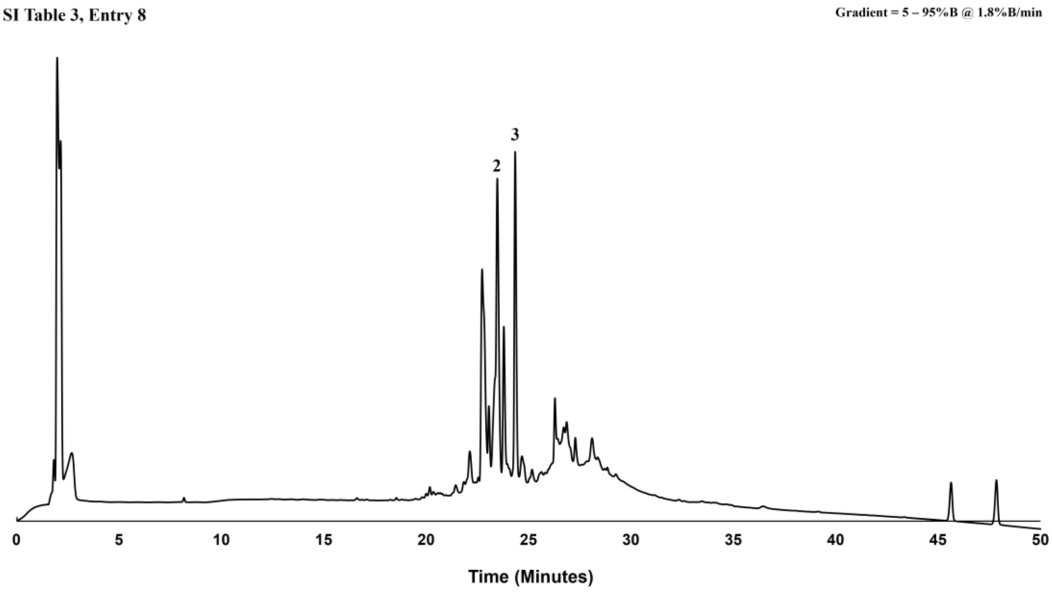

Supporting Information Figure S44. Analytical RP-HPLC chromatogram (214 nm) of crude peptide liberated from the peptidyl resin following treatment with conditions outlined in **Supporting Information Table S3, Entry 8**. Aeris Peptide XB-C18 (100 Å, 5 µm, 150 mm x 4.6 mm), linear gradient 5% – 95%B over 50 min (*ca.* 1.8%B/min) at 1 mL/min.

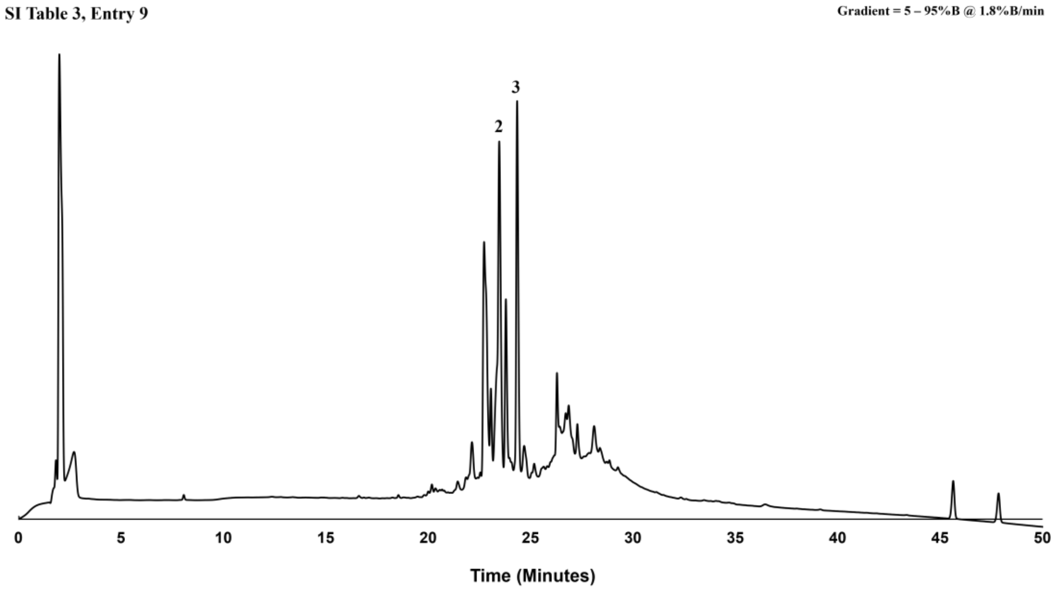

Supporting Information Figure S45. Analytical RP-HPLC chromatogram (214 nm) of crude peptide liberated from the peptidyl resin following treatment with conditions outlined in **Supporting Information Table S3, Entry 9**. Aeris Peptide XB-C18 (100 Å, 5 µm, 150 mm x 4.6 mm), linear gradient 5% – 95%B over 50 min (*ca.* 1.8%B/min) at 1 mL/min.

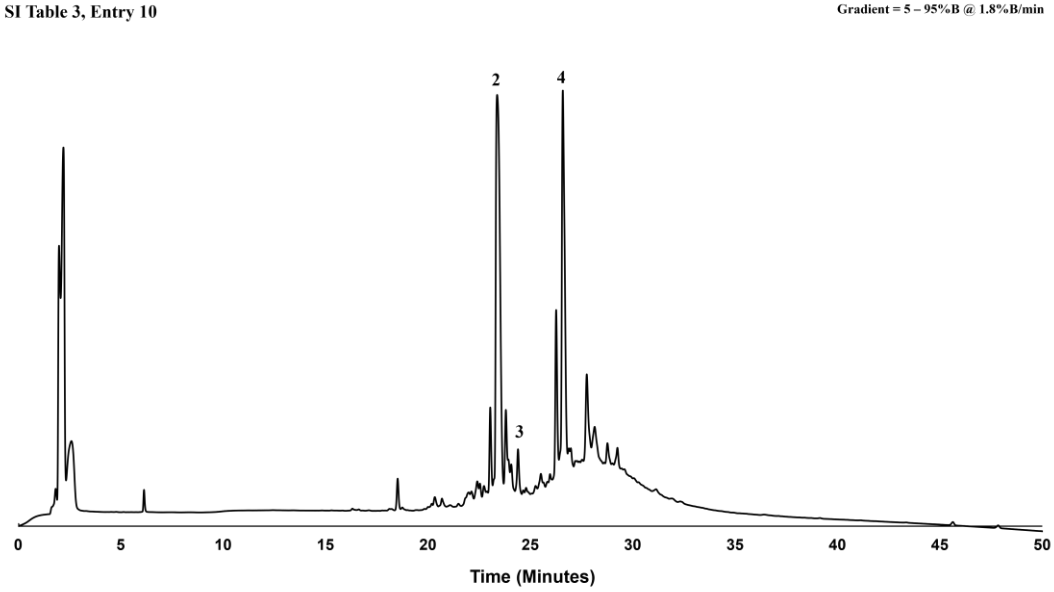

Supporting Information Figure S46. Analytical RP-HPLC chromatogram (214 nm) of crude peptide liberated from the peptidyl resin following treatment with conditions outlined in **Supporting Information Table S3, Entry 10**. Aeris Peptide XB-C18 (100 Å, 5 µm, 150 mm x 4.6 mm), linear gradient 5% – 95%B over 50 min (*ca.* 1.8%B/min) at 1 mL/min.

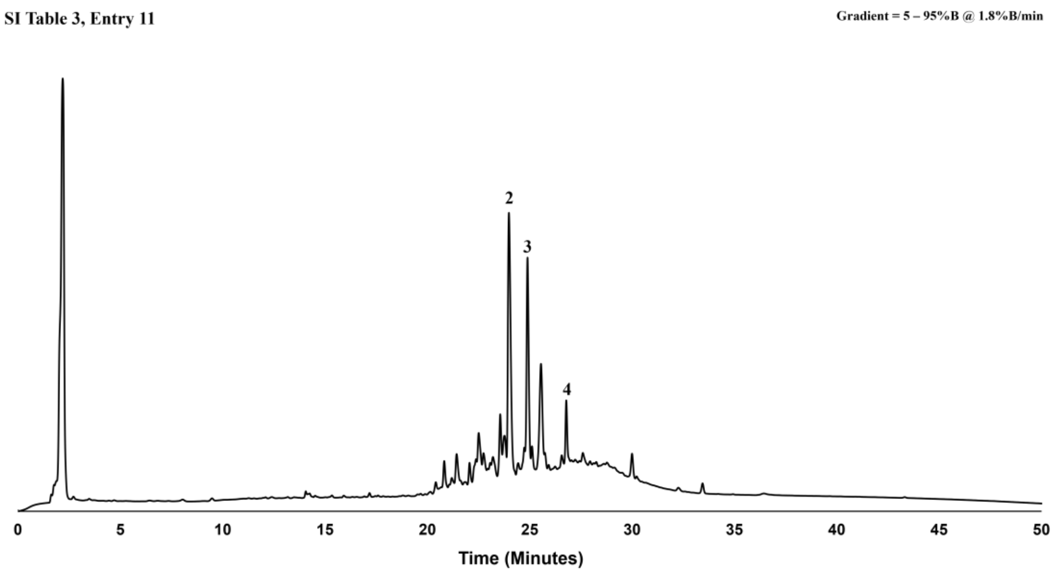

Supporting Information Figure S47. Analytical RP-HPLC chromatogram (214 nm) of crude peptide liberated from the peptidyl resin following treatment with conditions outlined in **Supporting Information Table S3, Entry 11**. Aeris Peptide XB-C18 (100 Å, 5 µm, 150 mm x 4.6 mm), linear gradient 5% – 95%B over 50 min (*ca.* 1.8%B/min) at 1 mL/min.

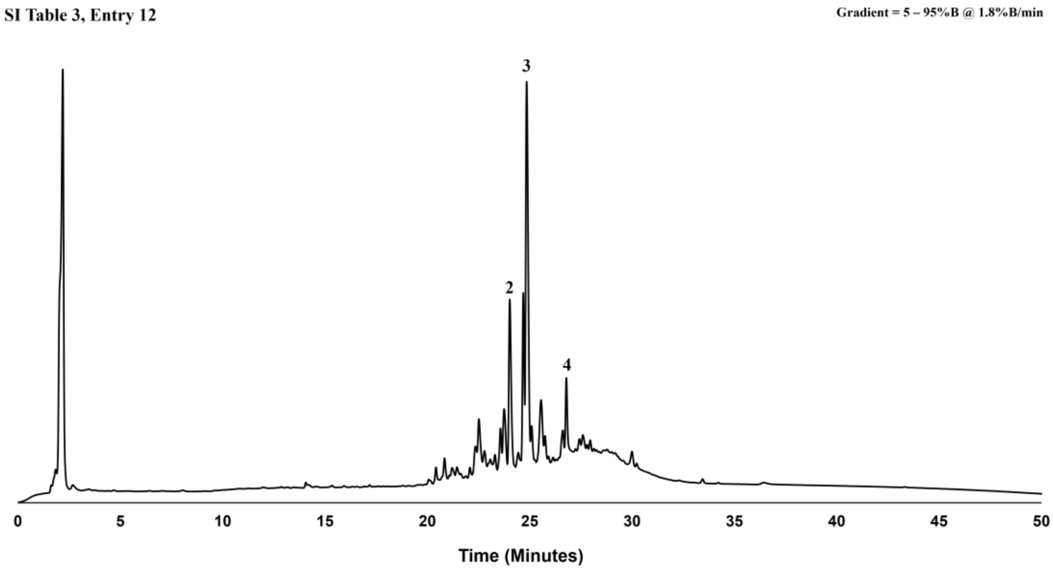

Supporting Information Figure S48. Analytical RP-HPLC chromatogram (214 nm) of crude peptide liberated from the peptidyl resin following treatment with conditions outlined in **Supporting Information Table S3, Entry 12**. Aeris Peptide XB-C18 (100 Å, 5 µm, 150 mm x 4.6 mm), linear gradient 5% – 95%B over 50 min (*ca.* 1.8%B/min) at 1 mL/min.

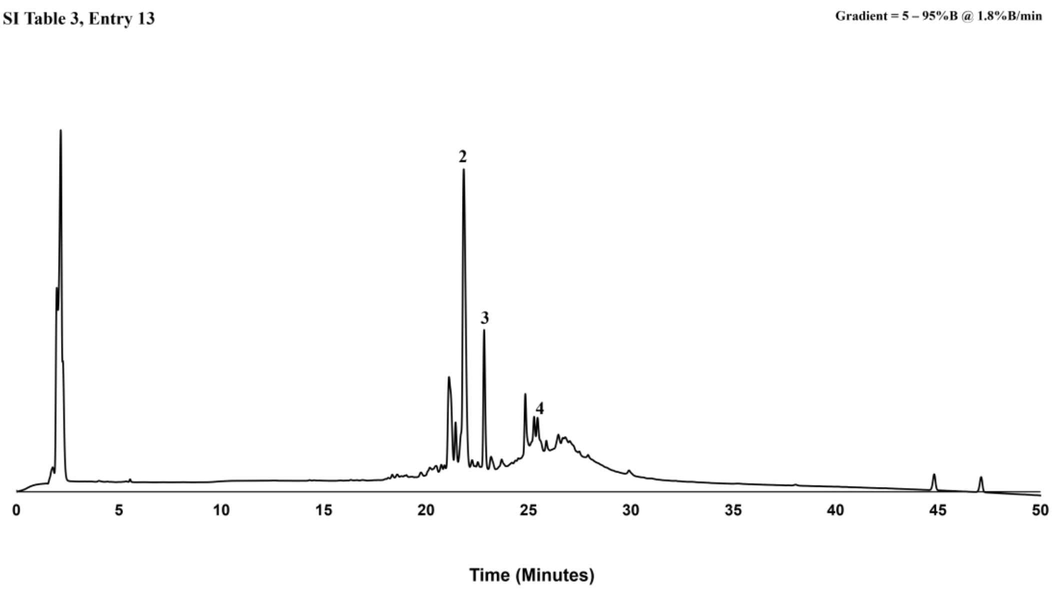

Supporting Information Figure S49. Analytical RP-HPLC chromatogram (214 nm) of crude peptide liberated from the peptidyl resin following treatment with conditions outlined in **Supporting Information Table S3, Entry 13**. Aeris Peptide XB-C18 (100 Å, 5 µm, 150 mm x 4.6 mm), linear gradient 5% – 95%B over 50 min (*ca.* 1.8%B/min) at 1 mL/min.

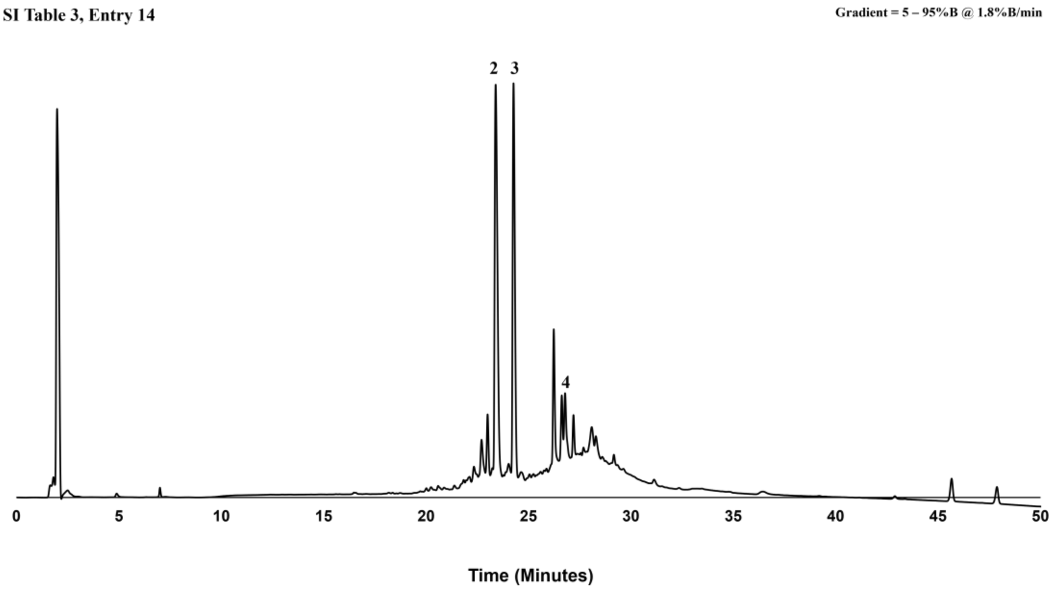

Supporting Information Figure S50. Analytical RP-HPLC chromatogram (214 nm) of crude peptide liberated from the peptidyl resin following treatment with conditions outlined in **Supporting Information Table S3, Entry 14**. Aeris Peptide XB-C18 (100 Å, 5 µm, 150 mm x 4.6 mm), linear gradient 5% – 95%B over 50 min (*ca.* 1.8%B/min) at 1 mL/min.

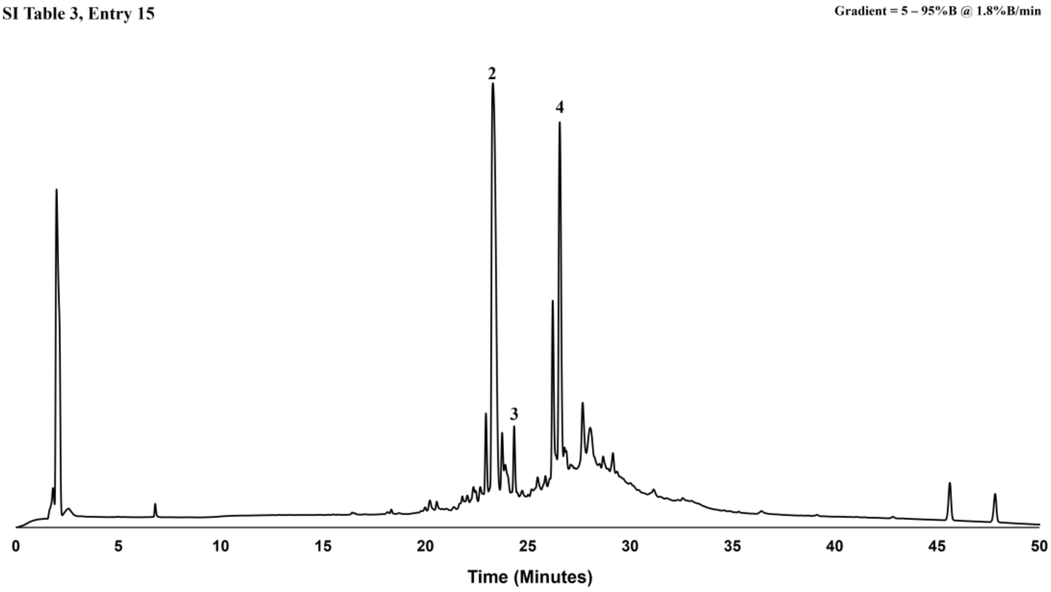

Supporting Information Figure S51. Analytical RP-HPLC chromatogram (214 nm) of crude peptide liberated from the peptidyl resin following treatment with conditions outlined in **Supporting Information Table S3, Entry 15**. Aeris Peptide XB-C18 (100 Å, 5 µm, 150 mm x 4.6 mm), linear gradient 5% – 95%B over 50 min (*ca.* 1.8%B/min) at 1 mL/min.

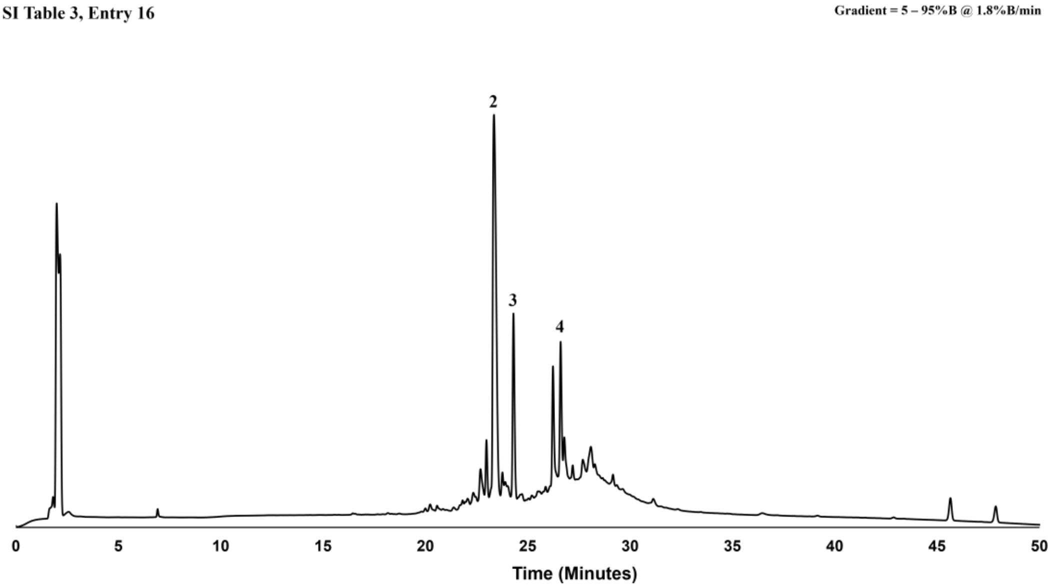

Supporting Information Figure S52. Analytical RP-HPLC chromatogram (214 nm) of crude peptide liberated from the peptidyl resin following treatment with conditions outlined in **Supporting Information Table S3, Entry 16**. Aeris Peptide XB-C18 (100 Å, 5 µm, 150 mm x 4.6 mm), linear gradient 5% – 95%B over 50 min (*ca.* 1.8%B/min) at 1 mL/min.

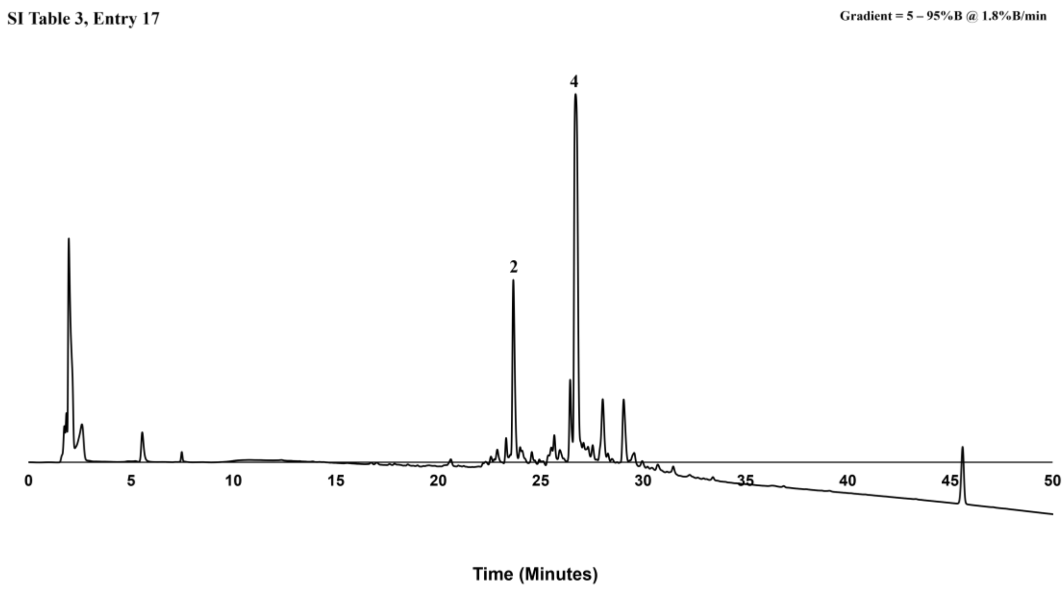

Supporting Information Figure S53. Analytical RP-HPLC chromatogram (214 nm) of crude peptide liberated from the peptidyl resin following treatment with conditions outlined in **Supporting Information Table S3, Entry 17**. Aeris Peptide XB-C18 (100 Å, 5 µm, 150 mm x 4.6 mm), linear gradient 5% – 95%B over 50 min (*ca.* 1.8%B/min) at 1 mL/min.

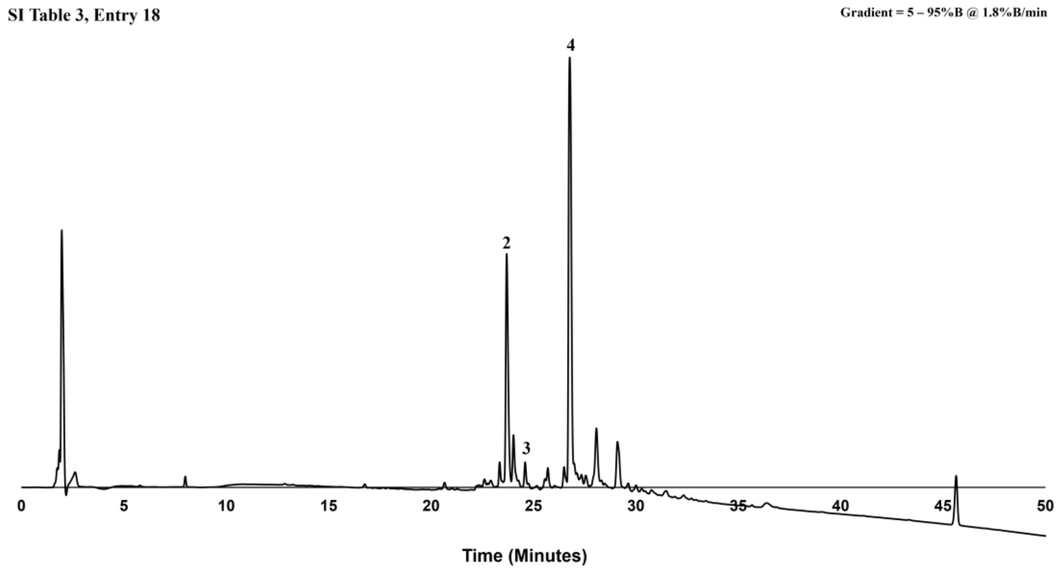

Supporting Information Figure S54. Analytical RP-HPLC chromatogram (214 nm) of crude peptide liberated from the peptidyl resin following treatment with conditions outlined in **Supporting Information Table S3, Entry 18**. Aeris Peptide XB-C18 (100 Å, 5 µm, 150 mm x 4.6 mm), linear gradient 5% – 95%B over 50 min (*ca.* 1.8%B/min) at 1 mL/min.

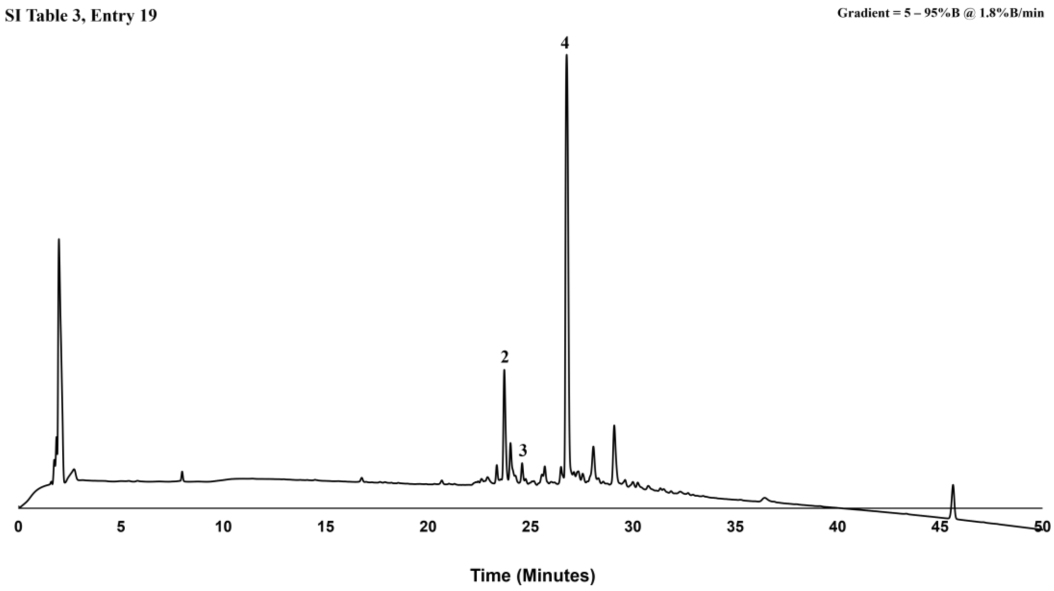

Supporting Information Figure S55. Analytical RP-HPLC chromatogram (214 nm) of crude peptide liberated from the peptidyl resin following treatment with conditions outlined in **Supporting Information Table S3, Entry 19**. Aeris Peptide XB-C18 (100 Å, 5 µm, 150 mm x 4.6 mm), linear gradient 5% – 95%B over 50 min (*ca.* 1.8%B/min) at 1 mL/min.

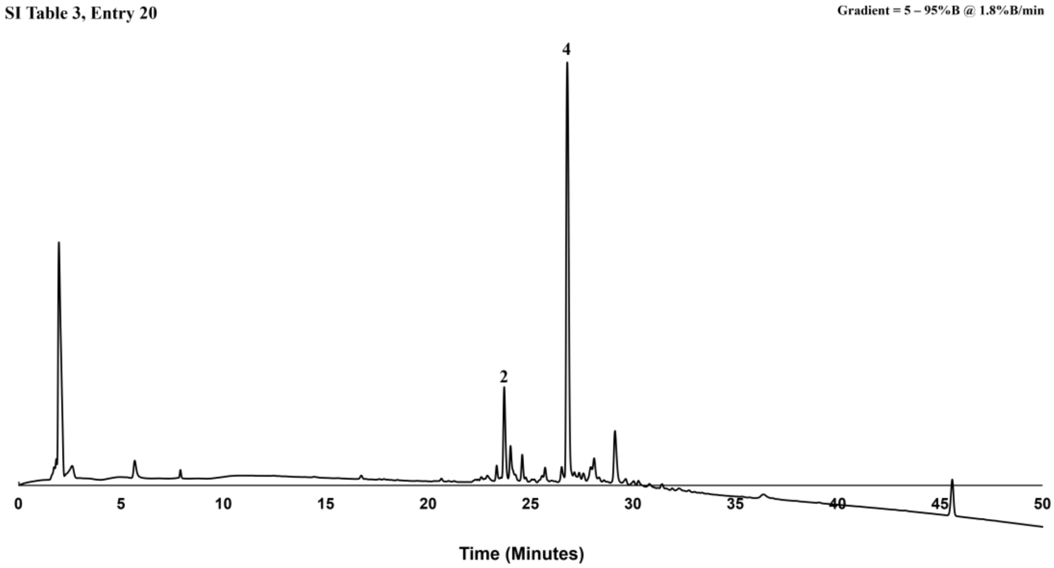

Supporting Information Figure S56. Analytical RP-HPLC chromatogram (214 nm) of crude peptide liberated from the peptidyl resin following treatment with conditions outlined in **Supporting Information Table S3, Entry 20**. Aeris Peptide XB-C18 (100 Å, 5 µm, 150 mm x 4.6 mm), linear gradient 5% – 95%B over 50 min (*ca.* 1.8%B/min) at 1 mL/min.

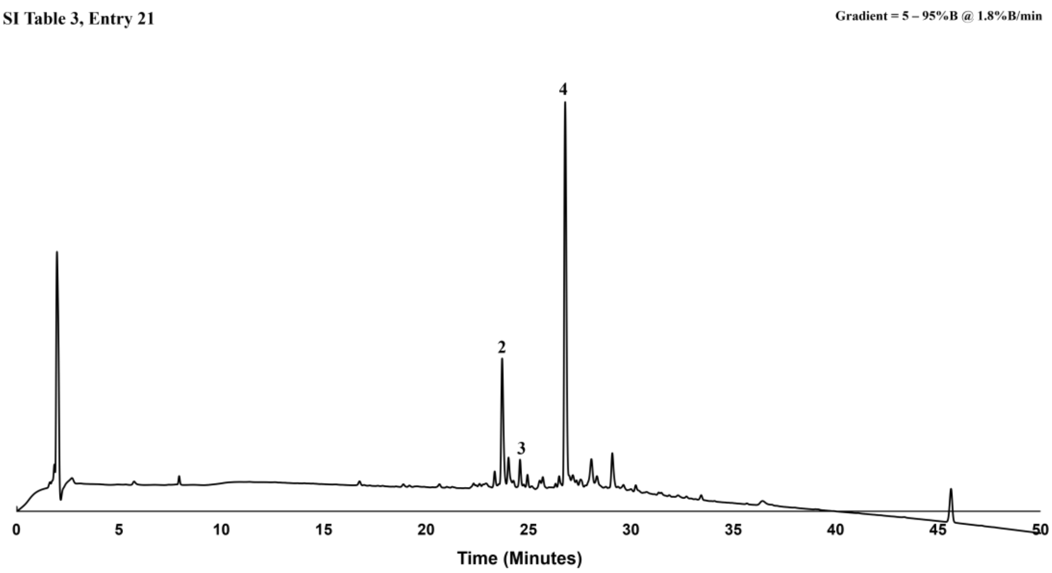

Supporting Information Figure S57. Analytical RP-HPLC chromatogram (214 nm) of crude peptide liberated from the peptidyl resin following treatment with conditions outlined in **Supporting Information Table S3, Entry 21**. Aeris Peptide XB-C18 (100 Å, 5 µm, 150 mm x 4.6 mm), linear gradient 5% – 95%B over 50 min (*ca.* 1.8%B/min) at 1 mL/min.

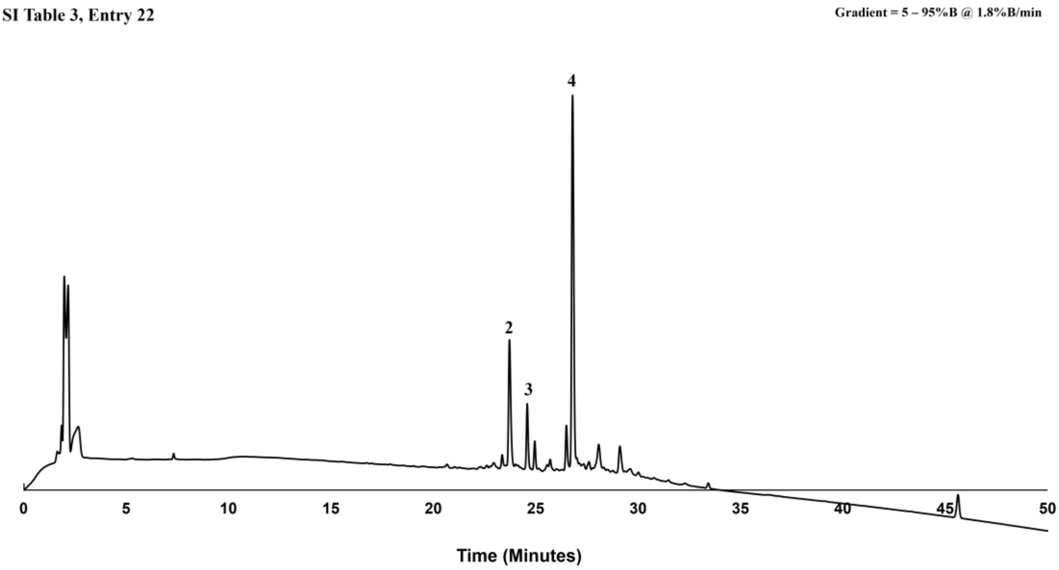

Supporting Information Figure S58. Analytical RP-HPLC chromatogram (214 nm) of crude peptide liberated from the peptidyl resin following treatment with conditions outlined in **Supporting Information Table S3, Entry 22**. Aeris Peptide XB-C18 (100 Å, 5 µm, 150 mm x 4.6 mm), linear gradient 5% – 95%B over 50 min (*ca.* 1.8%B/min) at 1 mL/min.

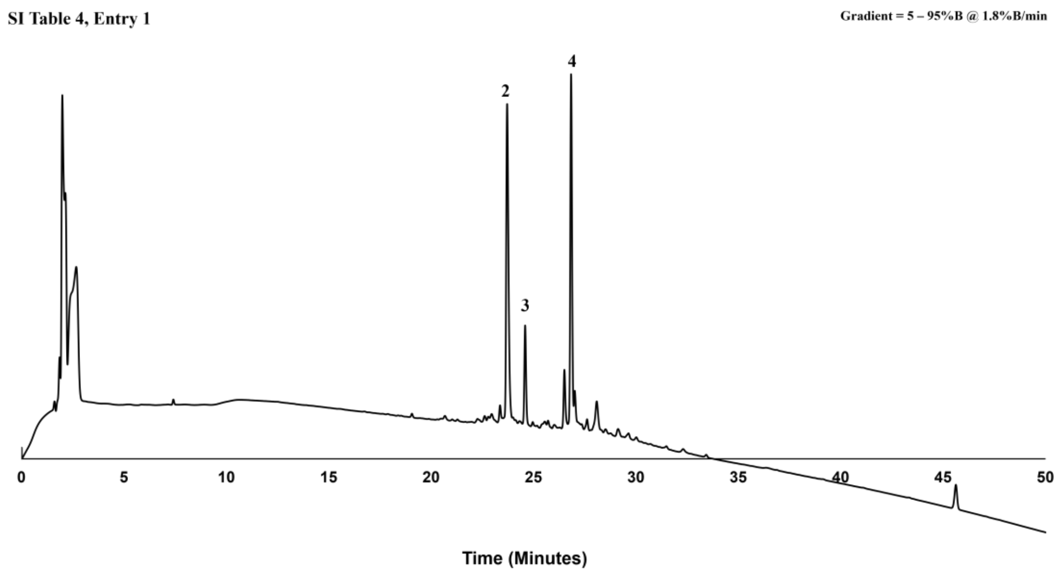

Supporting Information Figure S59. Analytical RP-HPLC chromatogram (214 nm) of crude peptide liberated from the peptidyl resin following treatment with conditions outlined in **Supporting Information Table S4, Entry 1**. Aeris Peptide XB-C18 (100 Å, 5 µm, 150 mm x 4.6 mm), linear gradient 5% – 95%B over 50 min (*ca.* 1.8%B/min) at 1 mL/min.

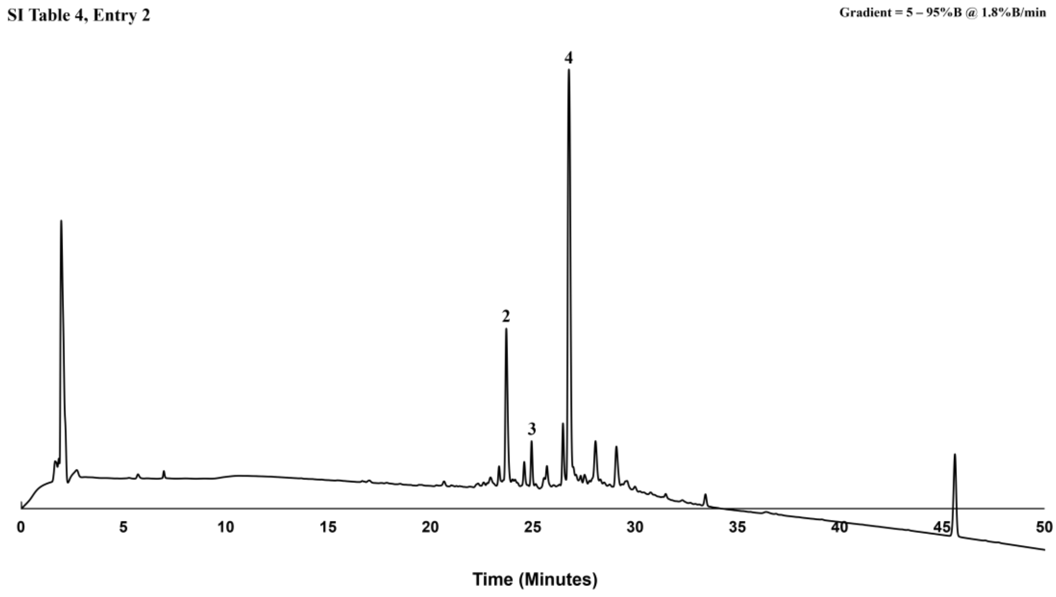

Supporting Information Figure S60. Analytical RP-HPLC chromatogram (214 nm) of crude peptide liberated from the peptidyl resin following treatment with conditions outlined in **Supporting Information Table S4, Entry 2**. Aeris Peptide XB-C18 (100 Å, 5 µm, 150 mm x 4.6 mm), linear gradient 5% – 95%B over 50 min (*ca.* 1.8%B/min) at 1 mL/min.

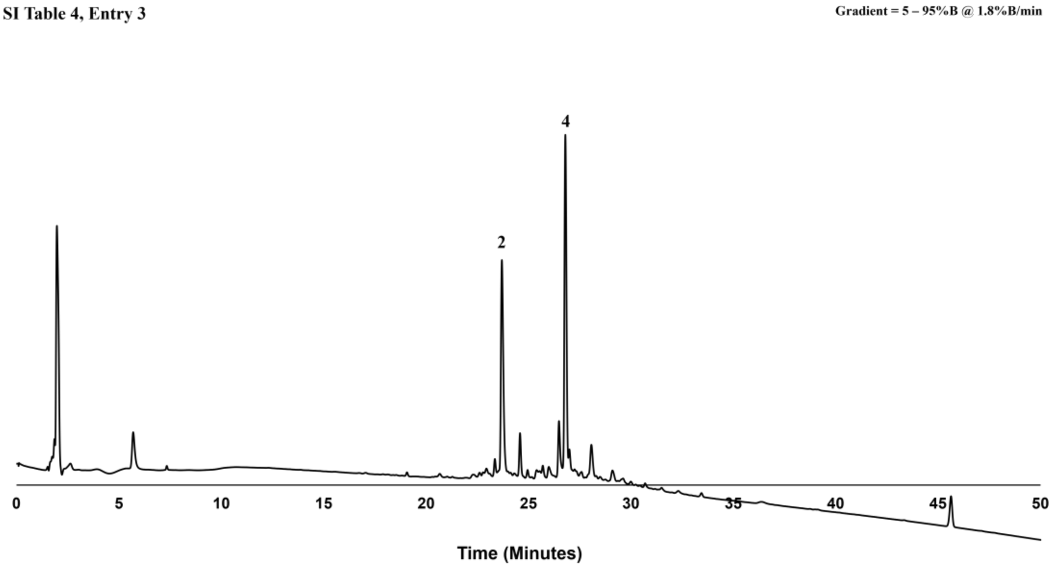

Supporting Information Figure S61. Analytical RP-HPLC chromatogram (214 nm) of crude peptide liberated from the peptidyl resin following treatment with conditions outlined in **Supporting Information Table S4, Entry 3**. Aeris Peptide XB-C18 (100 Å, 5 µm, 150 mm x 4.6 mm), linear gradient 5% – 95%B over 50 min (*ca.* 1.8%B/min) at 1 mL/min.

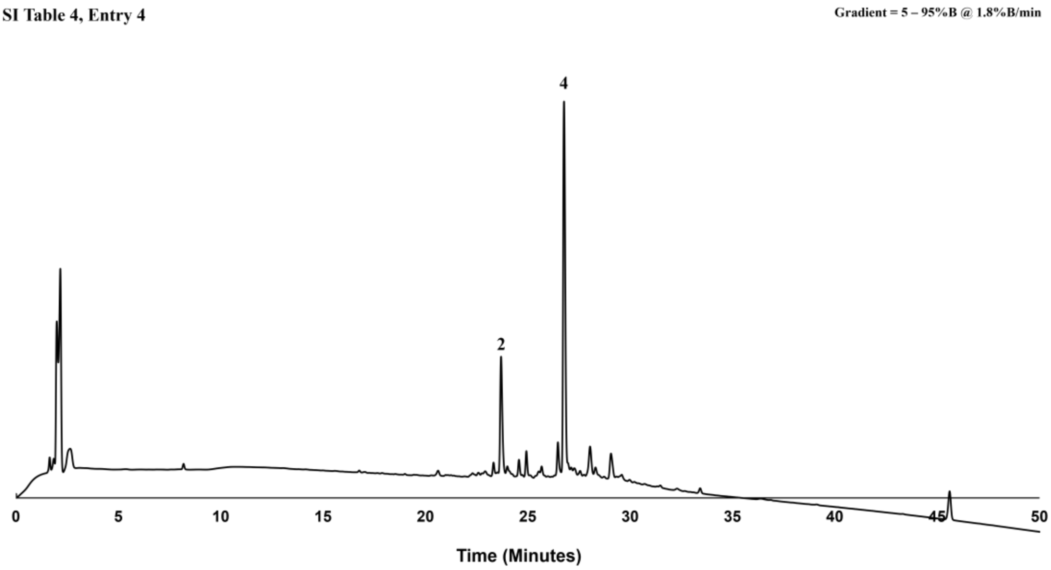

Supporting Information Figure S62. Analytical RP-HPLC chromatogram (214 nm) of crude peptide liberated from the peptidyl resin following treatment with conditions outlined in **Supporting Information Table S4, Entry 4**. Aeris Peptide XB-C18 (100 Å, 5 µm, 150 mm x 4.6 mm), linear gradient 5% – 95%B over 50 min (*ca.* 1.8%B/min) at 1 mL/min.

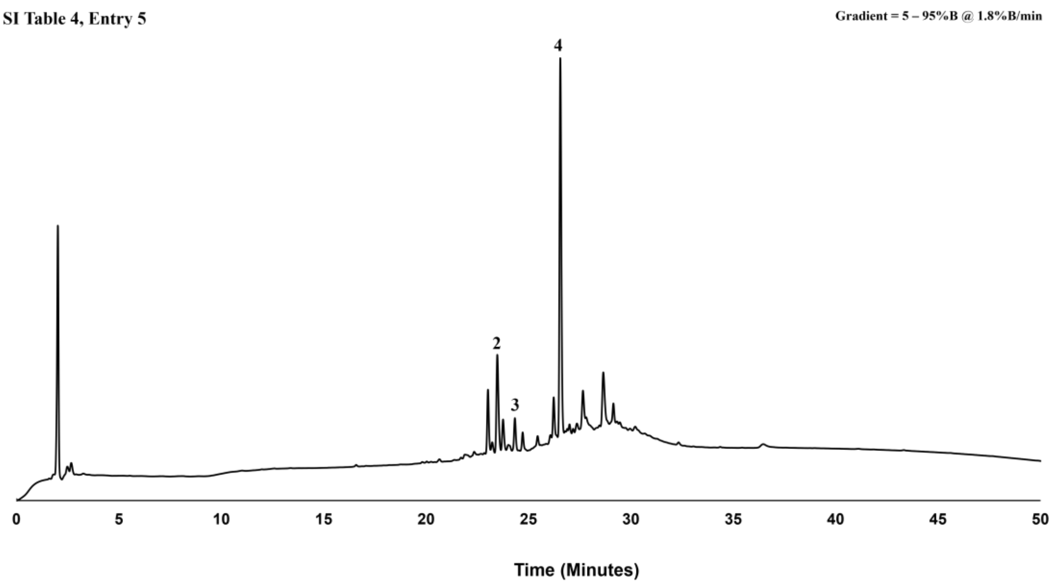

Supporting Information Figure S63. Analytical RP-HPLC chromatogram (214 nm) of crude peptide liberated from the peptidyl resin following treatment with conditions outlined in **Supporting Information Table S4, Entry 5**. Aeris Peptide XB-C18 (100 Å, 5 µm, 150 mm x 4.6 mm), linear gradient 5% – 95%B over 50 min (*ca.* 1.8%B/min) at 1 mL/min.

LCMS

Peaks identified from LCMS of peptidyl peaks found in analytical RP-HPLC traces of **Supporting Information Table S3, Entry 18**. Peak masses were consistent across all **Supporting Information Tables S3 and S4** entries at the respective retention times.

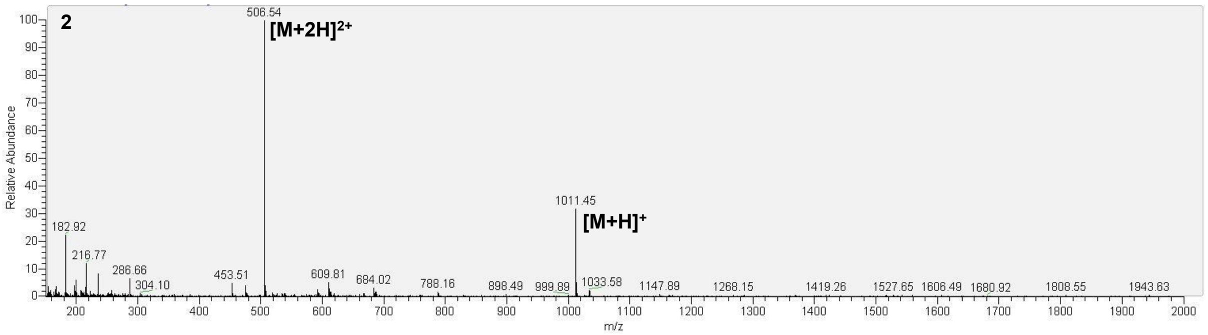

Supporting Information Figure S64. LCMS of peptidyl product at t_R = 23.6 min, following resin cleavage and global deprotection of peptidyl resin **4***, corresponding to undesired peptide product **2**. Mass calculated for [$C_{46}H_{70}N_{14}O_{12}$ + H] 1011.15; deconvoluted mass observed: 1010.77 ± 0.47. Charge states; 506.55 $[M+2H]^{2+}$, 1011.44 $[M+H]^{+}$.

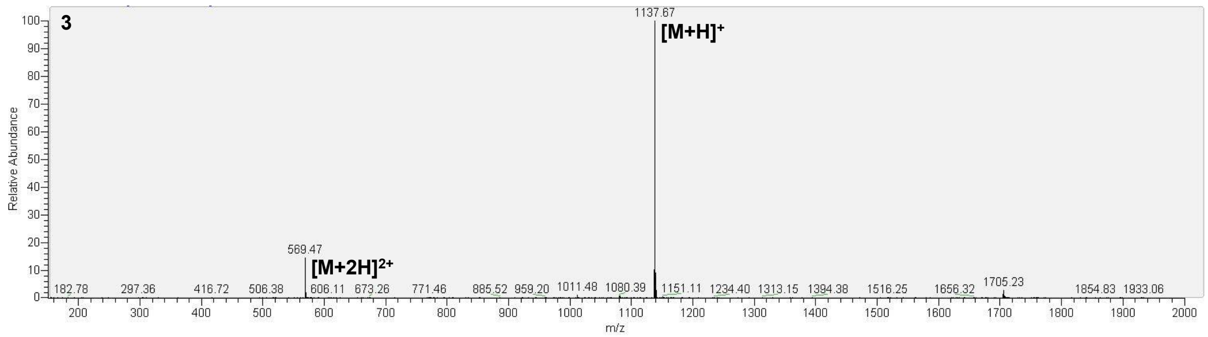

Supporting Information Figure S65. LCMS of peptidyl product at t_R = 24.3 min, following resin cleavage and global deprotection of peptidyl resin **4***, corresponding to undesired peptide product **3**. Mass calculated for [$C_{46}H_{69}IN_{14}O_{12}$ + H] 1137.05; deconvoluted mass observed: 1136.61 ± 0.45. Charge states; 569.46 $[M+2H]^{2+}$, 1137.29 $[M+H]^{+}$.

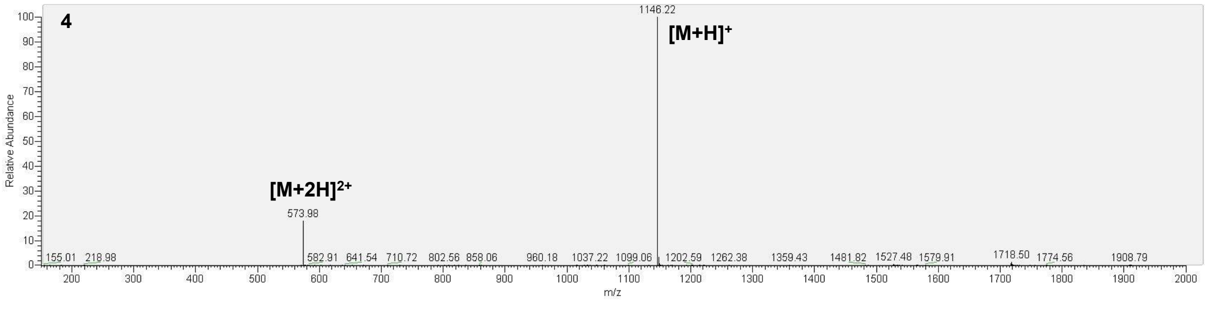

Supporting Information Figure S66. LCMS of peptidyl product at t_R = 26.7 min, following resin cleavage and global deprotection of peptidyl resin **4***, corresponding to desired peptide product **4**. Mass calculated for [$C_{54}H_{76}N_{14}O_{14}$ + H] 1145.29; deconvoluted mass observed: 1145.14 ± 0.31. Charge states; 573.68 $[M+2H]^{2+}$, 1145.92 $[M+H]^{+}$.

Diversification of Aromatic Functionalities

HPLC Traces

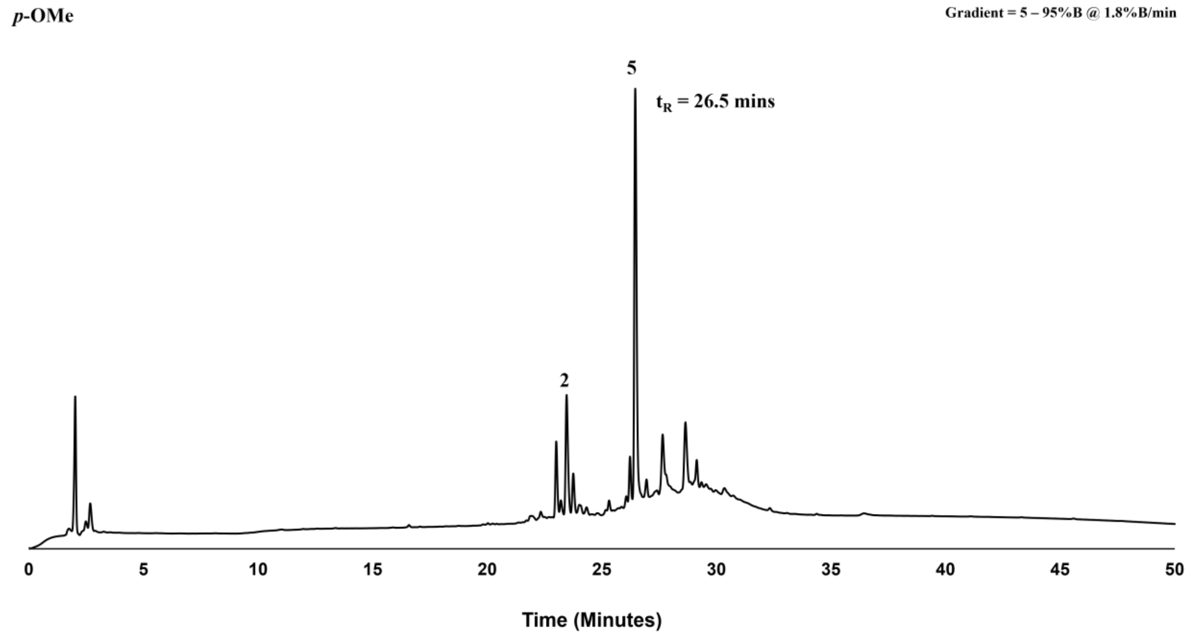

Supporting Information Figure S67. Analytical RP-HPLC chromatogram (214 nm) of crude peptide **5** following liberation from the peptidyl resin **5***. Aeris Peptide XB-C18 (100 Å, 5 µm, 150 mm x 4.6 mm), linear gradient 5% – 95%B over 50 min (*ca.* 1.8%B/min) at 1 mL/min. t_R = 26.5 min.

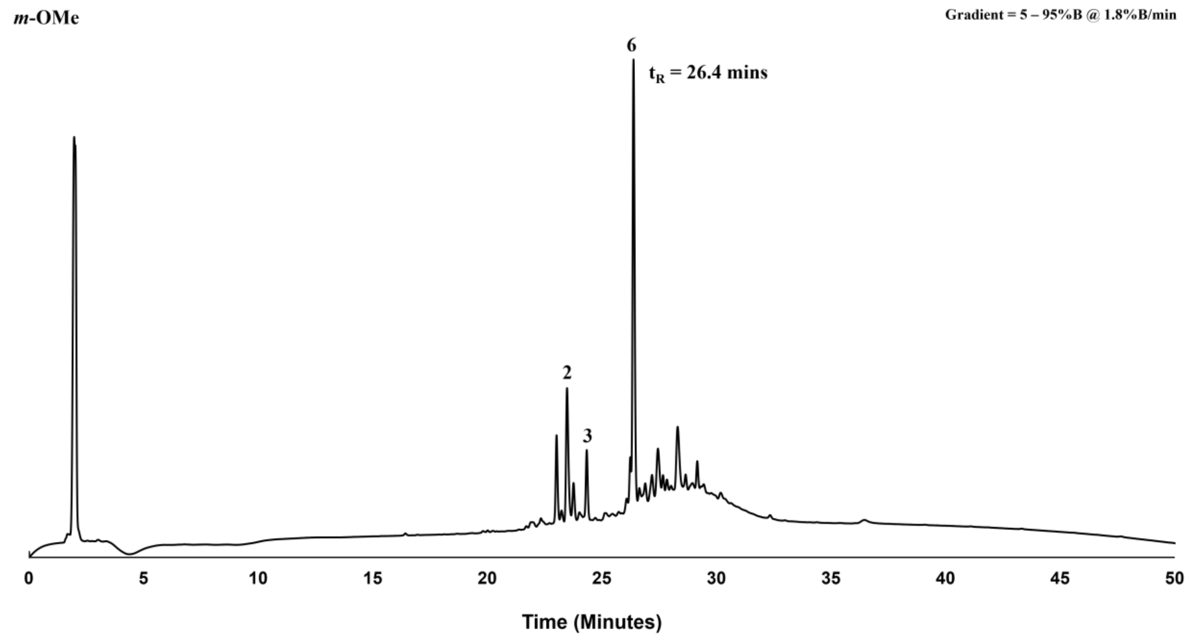

Supporting Information Figure S68. Analytical RP-HPLC chromatogram (214 nm) of crude peptide **6** following liberation from the peptidyl resin **6***. Aeris Peptide XB-C18 (100 Å, 5 µm, 150 mm x 4.6 mm), linear gradient 5% – 95%B over 50 min (*ca.* 1.8%B/min) at 1 mL/min. t_R = 26.4 min.

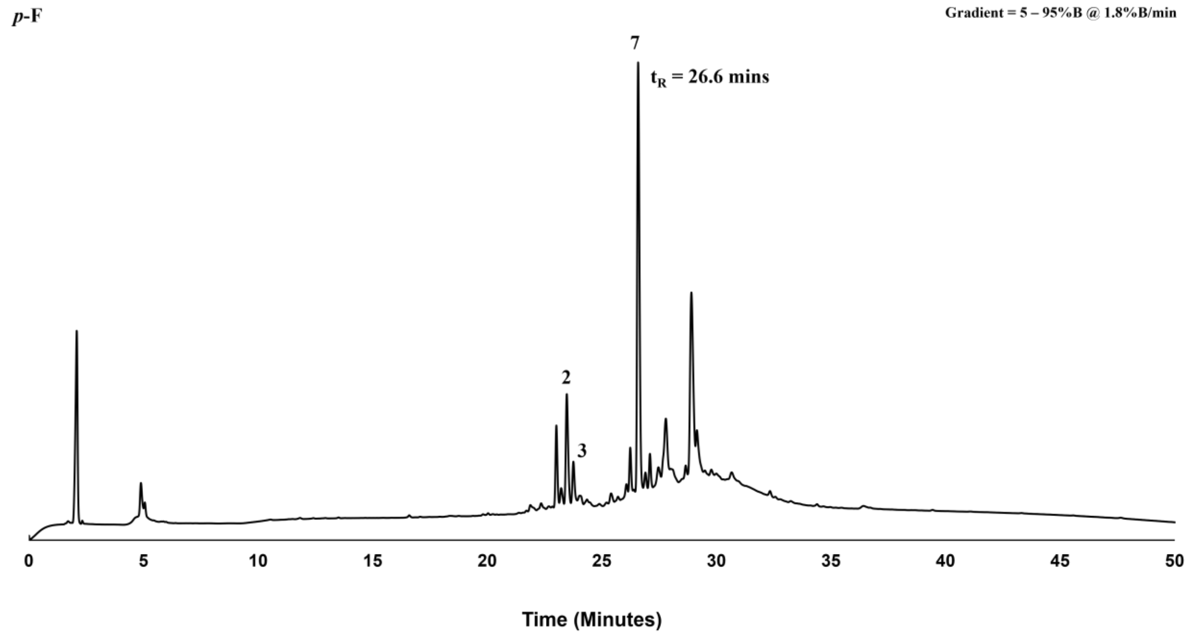

Supporting Information Figure S69. Analytical RP-HPLC chromatogram (214 nm) of crude peptide **7** following liberation from the peptidyl resin **7***. Aeris Peptide XB-C18 (100 Å, 5 µm, 150 mm x 4.6 mm), linear gradient 5% – 95%B over 50 min (*ca.* 1.8%B/min) at 1 mL/min. t_R = 26.6 min.

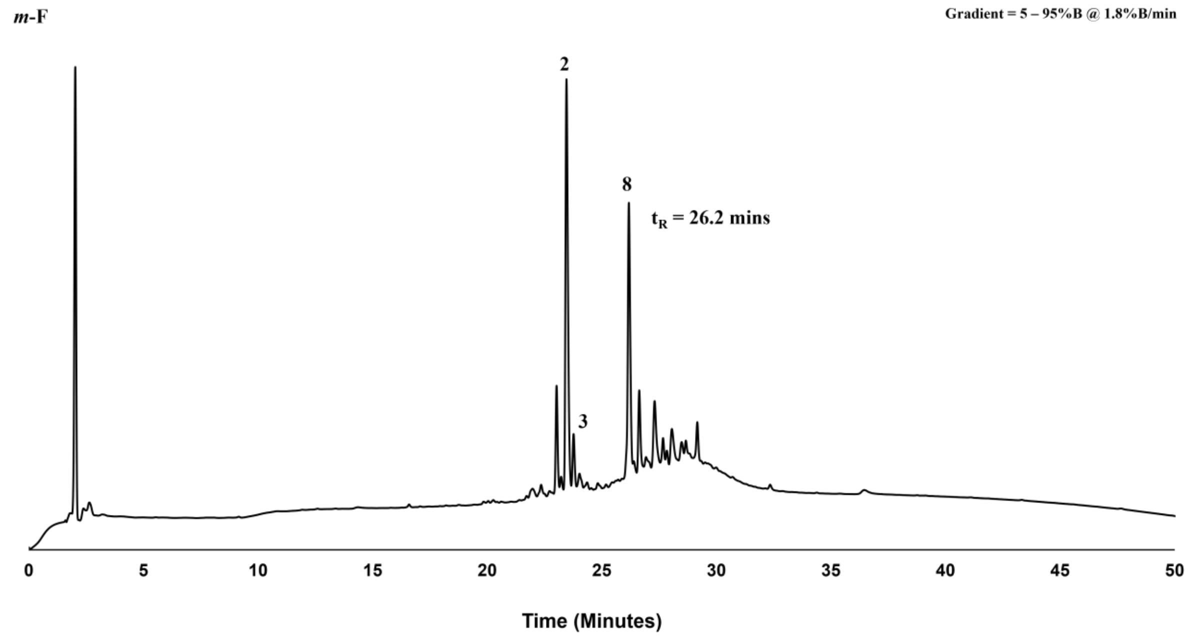

Supporting Information Figure S70. Analytical RP-HPLC chromatogram (214 nm) of crude peptide **8** following liberation from the peptidyl resin **8***. Aeris Peptide XB-C18 (100 Å, 5 µm, 150 mm x 4.6 mm), linear gradient 5% – 95%B over 50 min (*ca.* 1.8%B/min) at 1 mL/min. t_R = 26.2 min.

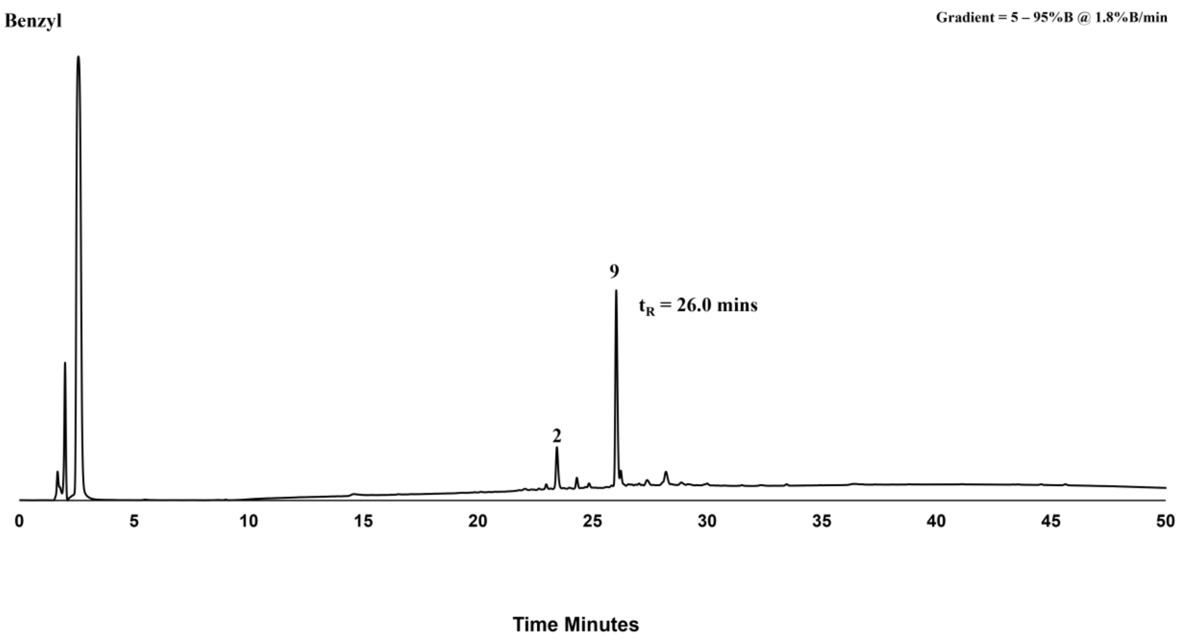

Supporting Information Figure S71. Analytical RP-HPLC chromatogram (214 nm) of crude peptide **9** following liberation from the peptidyl resin **9***. Aeris Peptide XB-C18 (100 Å, 5 µm, 150 mm x 4.6 mm), linear gradient 5% – 95%B over 50 min (*ca.* 1.8%B/min) at 1 mL/min. t_R = 26.0 min.

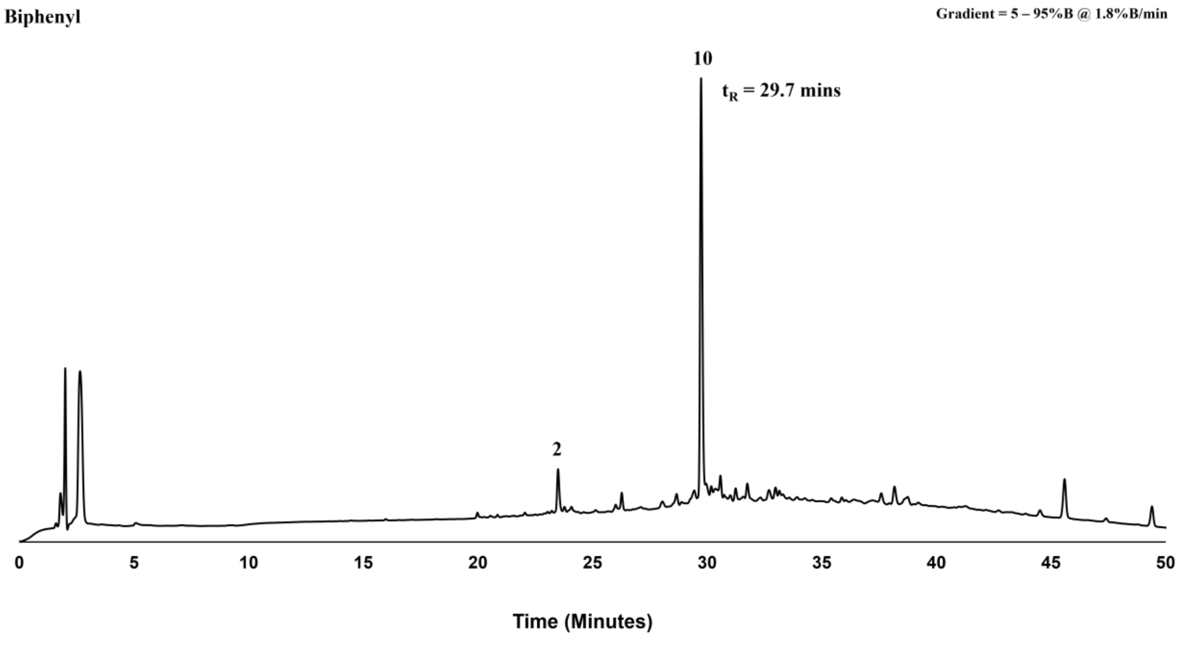

Supporting Information Figure S72. Analytical RP-HPLC chromatogram (214 nm) of crude peptide **10** following liberation from the peptidyl resin **10***. Aeris Peptide XB-C18 (100 Å, 5 µm, 150 mm x 4.6 mm), linear gradient 5% – 95%B over 50 min (*ca.* 1.8%B/min) at 1 mL/min. t_R = 29.7 min.

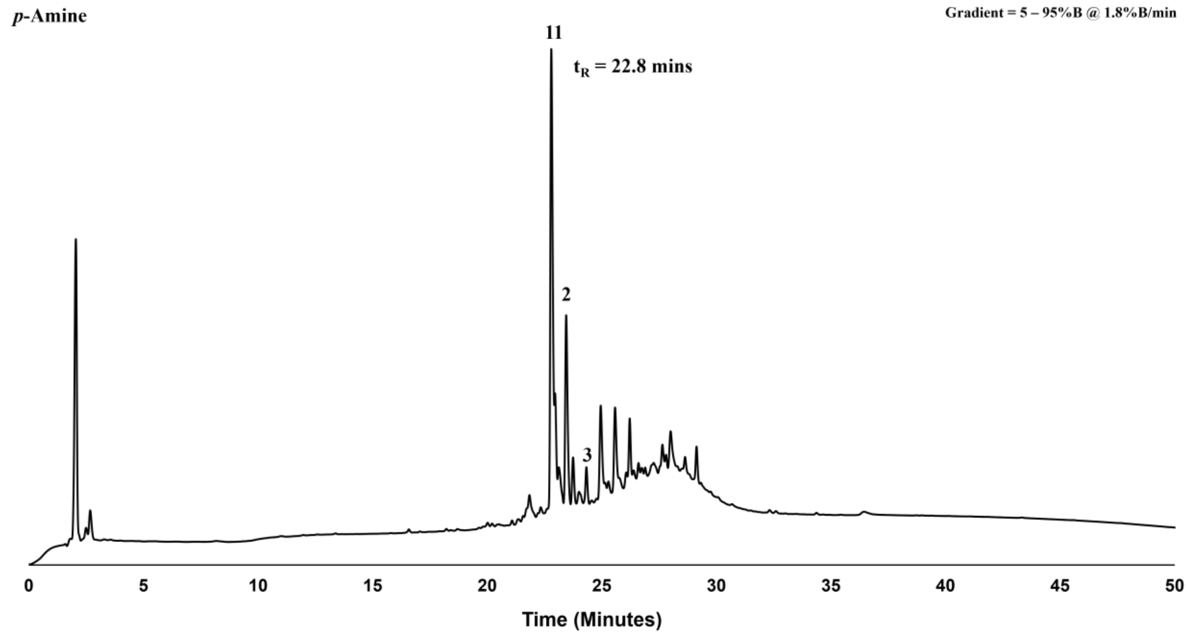

Supporting Information Figure S73. Analytical RP-HPLC chromatogram (214 nm) of crude peptide **11** following liberation from the peptidyl resin **11***. Aeris Peptide XB-C18 (100 Å, 5 µm, 150 mm x 4.6 mm), linear gradient 5% – 95%B over 50 min (*ca.* 1.8%B/min) at 1 mL/min. t_R = 22.8 min.

LCMS

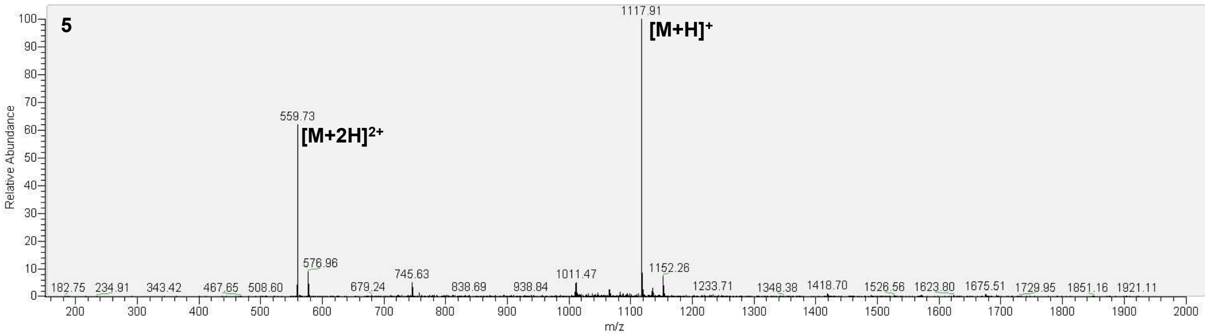

Supporting Information Figure S74. LCMS of major peptidyl product at t_R = 26.5 min, following resin cleavage and global deprotection of peptidyl resin **5***, corresponding to desired peptide **5**. Mass calculated for [$C_{53}H_{76}N_{14}O_{13}$ + H] 1117.28; deconvoluted mass observed: 1117.19 ± 0.39. Charge states; 559.73 $[M+2H]^{2+}$, 1117.91 $[M+H]^{+}$.

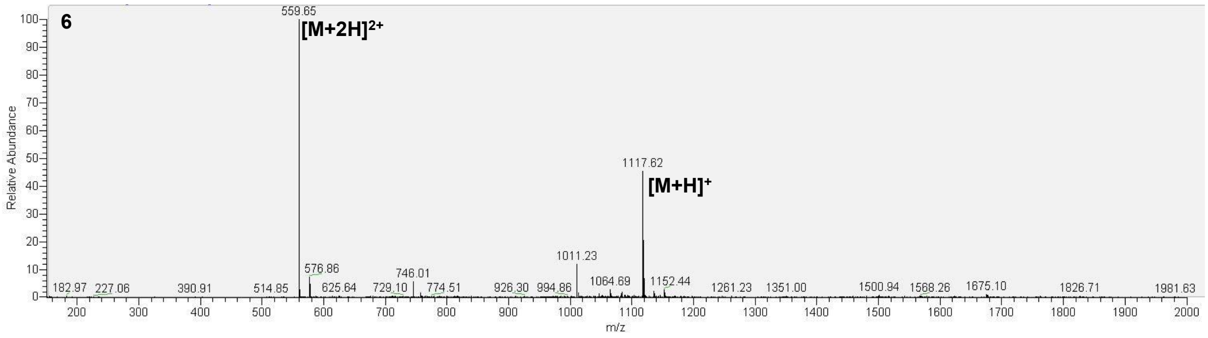

Supporting Information Figure S75. LCMS of major peptidyl product at t_R = 26.4 min, following resin cleavage and global deprotection of peptidyl resin **6***, corresponding to desired peptide **6**. Mass calculated for [$C_{53}H_{76}N_{14}O_{13}$ + H] 1117.28; deconvoluted mass observed: 1116.96 ± 0.48. Charge states; 559.65 $[M+2H]^{2+}$, 1117.62 $[M+H]^{+}$.

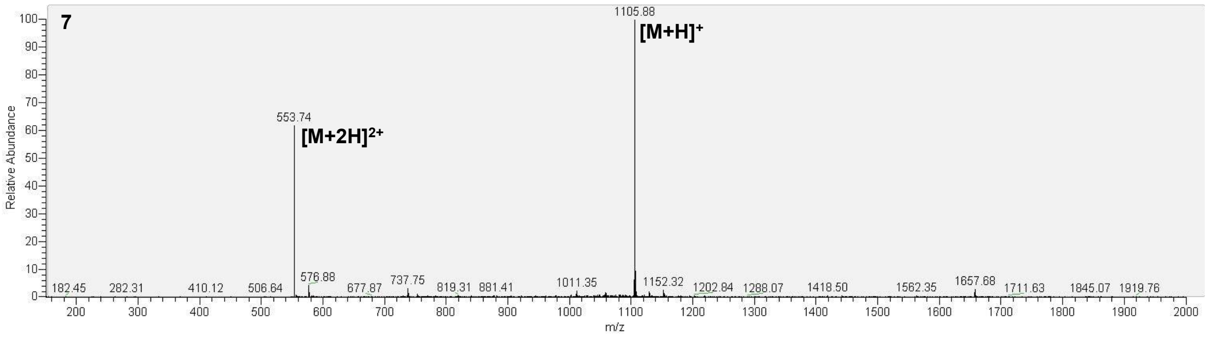

Supporting Information Figure S76. LCMS of major peptidyl product at t_R = 26.6 min, following resin cleavage and global deprotection of peptidyl resin **7***, corresponding to desired peptide **7**. Mass calculated for [$C_{52}H_{73}FN_{14}O_{12}$ + H] 1105.24; deconvoluted mass observed: 1105.18 ± 0.42. Charge states; 553.74 $[M+2H]^{2+}$, 1105.88 $[M+H]^{+}$.

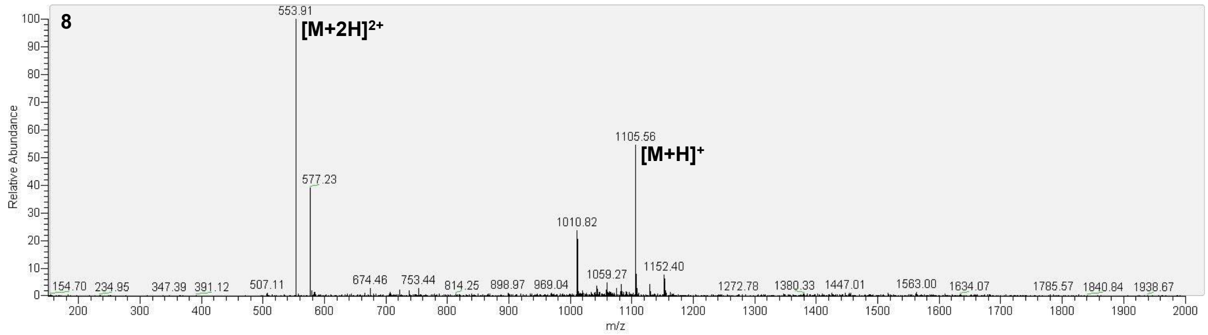

Supporting Information Figure S77. LCMS of major peptidyl product at t_R = 26.2 min, following resin cleavage and global deprotection of peptidyl resin **8***, corresponding to desired peptide **8**. Mass calculated for [$C_{52}H_{73}FN_{14}O_{12}$ + H] 1105.24; deconvoluted mass observed: 1105.19 ± 0.89. Charge states; 553.91 $[M+2H]^{2+}$, 1105.56 $[M+H]^{+}$.

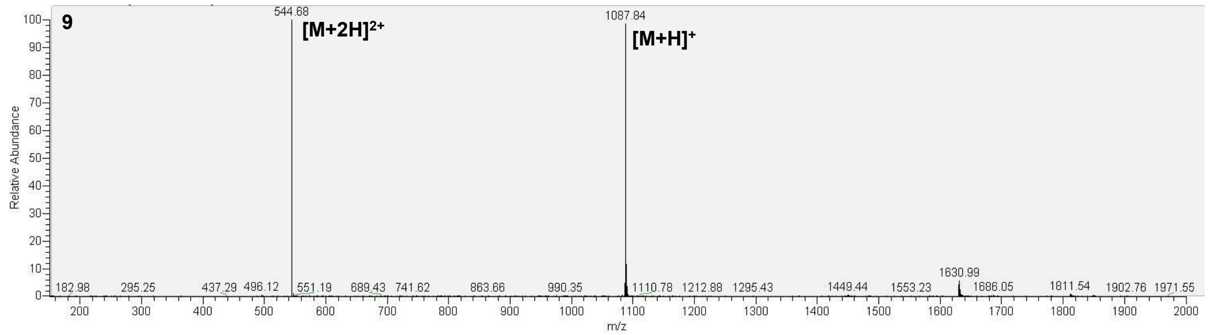

Supporting Information Figure S78. LCMS of major peptidyl product at t_R = 26.0 min, following resin cleavage and global deprotection of peptidyl resin **9***, corresponding to desired peptide **9**. Mass calculated for [$C_{52}H_{74}N_{14}O_{12}$ + H] 1087.25; deconvoluted mass observed: 1087.10 ± 0.37. Charge states; 544.68 $[M+2H]^{2+}$, 1087.84 $[M+H]^{+}$.

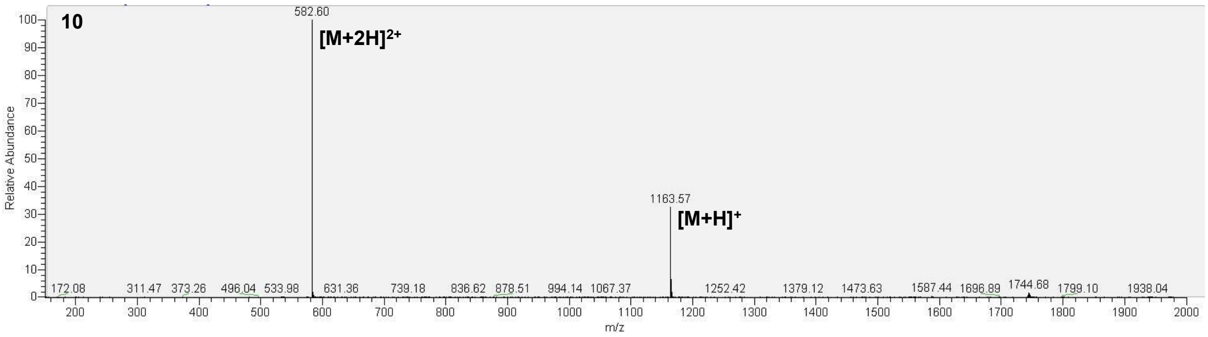

Supporting Information Figure S79. LCMS of major peptidyl product at t_R = 29.7 min, following resin cleavage and global deprotection of peptidyl resin **10***, corresponding to desired peptide **10**. Mass calculated for [$C_{58}H_{78}N_{14}O_{12}$ + H] 1163.35; deconvoluted mass observed: 1162.89 ± 0.45. Charge states; 582.60 $[M+2H]^{2+}$, 1163.57 $[M+H]^{+}$.

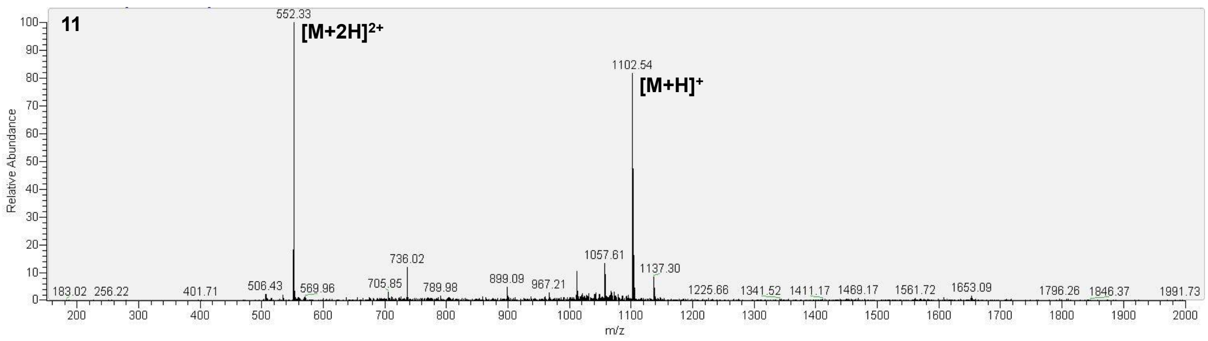

Supporting Information Figure S80. LCMS of major peptidyl product at t_R = 22.8 min, following resin cleavage and global deprotection of peptidyl resin **11***, corresponding to desired peptide **11**. Mass calculated for [$C_{52}H_{75}N_{15}O_{12}$ + H] 1102.27; deconvoluted mass observed: 1102.10 ± 0.79. Charge states; 552.33 $[M+2H]^{2+}$, 1102.54 $[M+H]^{+}$.

References

(1) Tornøe, C. W.; Christensen, C.; Meldal, M. Peptidotriazoles on Solid Phase: [1,2,3]-Triazoles by Regiospecific Copper(I)-Catalyzed 1,3-Dipolar Cycloadditions of Terminal Alkynes to Azides. *J. Org. Chem.* **2002**, *67* (9), 3057–3064. https://doi.org/10.1021/jo011148j.

(2) Shepperson, O. A.; Hanna, C. C.; Brimble, M. A.; Harris, P. W. R.; Cameron, A. J. Total Synthesis of Novel Antimicrobial β-Hairpin Capitellacin Via Rapid Flow-Based SPPS Assembly and Regioselective On-Resin Disulfide Cyclisation. *Int. J. Pept. Res. Ther.* **2021**, *28* (1), 32–42. https://doi.org/10.1007/s10989-021-10335-4.

(3) Li, L.; Zhang, G.; Zhu, A.; Zhang, L. A Convenient Preparation of 5-Iodo-1,4-Disubstituted-1,2,3-Triazole: Multicomponent One-Pot Reaction of Azide and Alkyne Mediated by CuI−NBS. *J. Org. Chem.* **2008**, *73* (9), 3630–3633. https://doi.org/10.1021/jo800035v.
